# Identification of RSK and TTK as Modulators of Blood Vessel Morphogenesis Using an Embryonic Stem Cell-Based Vascular Differentiation Assay

**DOI:** 10.1016/j.stemcr.2016.08.004

**Published:** 2016-09-08

**Authors:** Lamis Hammoud, Jessica R. Adams, Amanda J. Loch, Richard C. Marcellus, David E. Uehling, Ahmed Aman, Christopher Fladd, Trevor D. McKee, Christine E.B. Jo, Rima Al-Awar, Sean E. Egan, Janet Rossant

**Affiliations:** 1 Program in Developmental and Stem Cell Biology, Peter Gilgan Centre for Research and Learning, The Hospital for Sick Children, 686 Bay Street, Toronto, ON M5G 0A4, Canada; 2 Department of Molecular Genetics, University of Toronto, Toronto, ON M5S 1A8, Canada; 3 Drug Discovery Department, Ontario Institute for Cancer Research, Toronto, ON M5G 0A3, Canada; 4 SPARC BioCentre, The Hospital for Sick Children, Toronto, ON M5G 0A4, Canada; 5 Radiation Medicine Program, STTARR Innovation Centre, Princess Margaret Cancer Centre, Toronto, ON M5G 1L7, Canada

## Abstract

Blood vessels are formed through vasculogenesis, followed by remodeling of the endothelial network through angiogenesis. Many events that occur during embryonic vascular development are recapitulated during adult neoangiogenesis, which is critical to tumor growth and metastasis. Current antiangiogenic tumor therapies, based largely on targeting the vascular endothelial growth factor pathway, show limited clinical benefits, thus necessitating the discovery of alternative targets. Here we report the development of a robust embryonic stem cell-based vascular differentiation assay amenable to small-molecule screens to identify novel modulators of angiogenesis. In this context, RSK and TTK were identified as angiogenic modulators. Inhibition of these pathways inhibited angiogenesis in embryoid bodies and human umbilical vein endothelial cells. Furthermore, inhibition of RSK and TTK reduced tumor growth, vascular density, and improved survival in an in vivo Lewis lung carcinoma mouse model. Our study suggests that RSK and TTK are potential targets for antiangiogenic therapy, and provides an assay system for further pathway screens.

## Highlights

• Development of ESC-based vascular differentiation assay amenable to drug screening
• Screening a kinase library identified RSK and TTK as angiogenic modulators
• RSK and TTK inhibition disrupted angiogenesis in vitro
• RSK and TTK inhibition inhibited Lewis lung tumor growth and angiogenesis in vivo

## Teaser

Rossant and colleagues developed a robust embryonic stem cell-based vascular differentiation assay, which was used to screen a small-molecule kinase library. They identified RSK and TTK as angiogenic modulators. Inhibition of these pathways inhibited angiogenesis in embryoid bodies and HUVECs in vitro, and reduced tumor growth and vascular density in an in vivo Lewis lung carcinoma mouse model.

## Introduction

Pluripotent embryonic stem cells (ESCs) provide essential tools for understanding mammalian developmental processes, as they can differentiate in vitro into many tissues in a normal developmental manner (Keller, 2005, Solter, 2006). These cells are amenable to high-throughput screens using RNAi or small-molecule libraries to dissect molecular pathways (Ding and Buchholz, 2006, Xu et al., 2008). Early vascular and hematopoietic differentiation of ESCs has been extensively studied (Keller, 2005), making these pathways particularly attractive for large-scale screens.

Blood vessels are first formed through vasculogenesis, whereby angioblasts (endothelial precursors) aggregate in the developing embryo to form a primitive network of endothelial tubes. This network is later remodeled through a complex process termed angiogenesis, which includes sprouting of new blood vessels, to form the mature circulatory network (Rossant and Howard, 2002). Major breakthroughs in our understanding of vascular development and remodeling have arisen from characterization of vascular mutant phenotypes in mice. Vascular endothelial growth factor (VEGF), acting through the FLK-1/VEGF receptor 2 (VEGFR2), is crucial for blood vessel formation and development (Carmeliet et al., 1996, Shalaby et al., 1995). NOTCH/DLL4 signaling plays a critical role in branching/sprouting morphogenesis, whereby loss of NOTCH signaling leads to excess tip cell formation and non-productive vessel development (Hellstrom et al., 2007). Impaired vascular development was also reported for mutations in ANG/TIE, platelet-derived growth factor (PDGF), transforming growth factor β (TGF-β), EFN, HH, and PLXN/SEMA signaling pathways (reviewed by Rossant and Howard, 2002).

Many signaling pathways required during embryonic vascular development are also essential during adult neoangiogenesis (Carmeliet, 2003). Adult neovascularization occurs in many physiological and pathological settings, such as wound healing (Ruiter et al., 1993), recovery from myocardial infarction (Chung et al., 2002), tumor growth, and metastasis (Ruiter et al., 1993). There is increasing interest in using modulators of angiogenesis to treat cancer (Ferrara, 2004). Currently antiangiogenic therapy has two opposing target pathways, the VEGF/FLK-1 and DLL4/NOTCH pathways (Kuhnert et al., 2011). The new generation of antiangiogenic drugs that have arisen from an understanding of vascular developmental biology, such as bevacizumab (anti-VEGF) (Ferrara et al., 2005), have demonstrated some efficacy in cancer patients, but cause serious side effects and frequent relapses (Kerbel, 2008). Similar results have been obtained from inhibition of the NOTCH/DLL4 pathway (Andersson and Lendahl, 2014), thus necessitating the discovery of alternative therapeutic targets.

To this end we have developed a robust, highly reproducible, mouse ESC-based vascular differentiation assay that is sensitive to both inhibition and promotion of vascular sprouting as well as to changes in vessel morphology. Using our embryoid body (EB)-based assay, we undertook a kinase inhibitor screen to identify small molecules that could block or enhance blood vessel sprouting morphogenesis. The screen yielded numerous hits, which we validated in vitro and subsequently tested for in vivo antiangiogenic activity in a Lewis lung (LL/2) carcinoma model. We have identified RSK and TTK as potential targets for antiangiogenic tumor therapy, and provide an assay system for further pathway screens.

## Results

### Development of a Robust, and Reproducible Vascular Differentiation Assay Using ESCs

**Figure 1:**
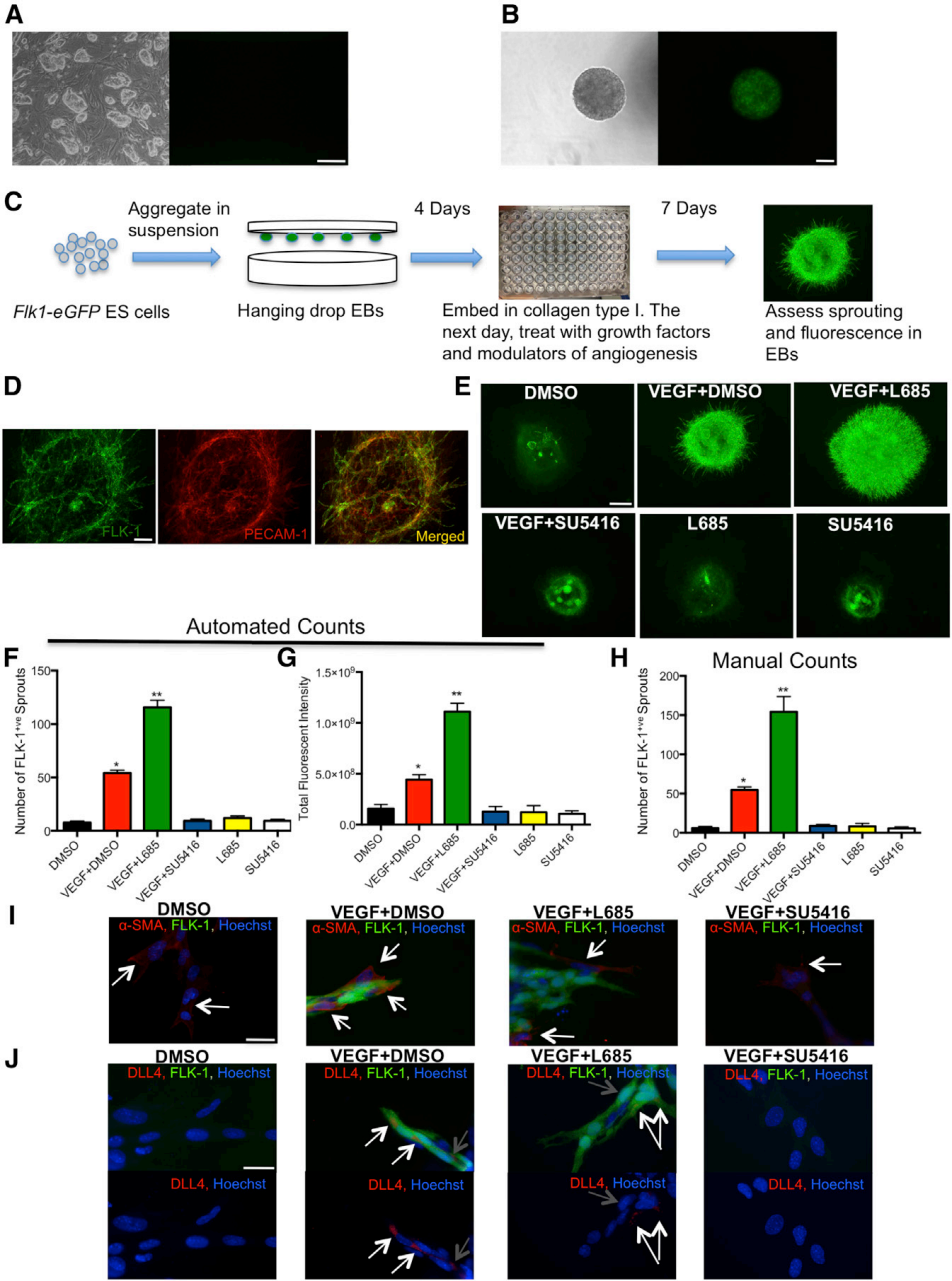
Development and Validation of ESC-Based Vascular Differentiation Assay (A) Undifferentiated *Flk1-eGFP* ESCs under phase contrast (left panel) and GFP filter (right panel). Scale bar, 100 μm. (B) *Flk1-eGFP* ESCs were aggregated in suspension in hanging drops for 4 days and observed under phase contrast (left panel) and GFP filter (right panel). Scale bar, 100 μm. (C) Schematic representation of vascular differentiation assay method. (D) PECAM-1 staining of *Flk1-eGFP*-derived EBs embedded in collagen type I gel and treated with VEGF (50 ng/mL), bFGF (100 ng/mL), IL-6 (10 ng/mL), and EPO (2 U/mL). Scale bar, 500 μm. (E) Validation of the assay using two small-molecule inhibitors, a NOTCH inhibitor (L685, 5 μM), and an FLK-1 inhibitor (SU5416, 4 μM). Scale bar, 500 μm. (F and G) Cellomics was used to quantify FLK-1^+^ sprouts (F) and FLK-1 expression (measured as total fluorescent intensity) (G). (H) Quantification of FLK-1^+^ sprouts obtained by manual counts. Data are mean ± SEM, n ≥ 3. ^∗^p < 0.0001, statistically significant compared with DMSO control; ^∗∗^p < 0.0001, statistically significant compared with DMSO and VEGF + DMSO controls. (I and J) Characterization of vascular sprouts of *Flk1-eGFP*-derived EBs treated as indicated. (I) Anti-α-SMA staining (white arrows point to mural cells). Scale bar, 25 μm. (J) Anti-Dll4 staining (white arrows point to tip cells, gray arrows point to stalk cells). Scale bar, 25 μm. See also Figure S1.

We have previously reported the generation of ESCs whereby EGFP was inserted into the *Flk-1* locus, and showed that this reporter faithfully recapitulates all areas of FLK-1 expression (Ema et al., 2006). As predicted, no EGFP was observed in the undifferentiated ESCs (Figure 1A), and high levels of EGFP were observed when ESCs were differentiated into EBs (Figure 1B). To optimize the vascular differentiation assay (Figure 1C), we aggregated *Flk1-eGFP* ESCs in suspension as hanging drops to form EBs. Different cell concentrations, types of matrices, and different days for embedding of EBs were tested (see Supplemental Experimental Procedures). We determined that EBs generated from 200 cells and embedded in collagen type I gels at day 4 gave the most consistent and reproducible results. There was no significant difference in the number of FLK-1 positive (FLK-1^+^) sprouts between EBs treated with VEGF only and EBs treated with VEGF in the presence of one or more of the previously established angiogenic growth factors (basic fibroblast growth factor [bFGF], interleukin-6 [IL-6], and erythropoietin [EPO]) (Feraud et al., 2001) (Figure S1A), suggesting that VEGF alone accounts for the majority of the angiogenic response and is the only factor required in our assay. PECAM-1 staining showed nearly complete overlap with the *Flk1-eGFP* reporter both in the primary vascular plexus formed in the EBs and the angiogenic sprouts extending from the EBs (Figure 1D). We also determined that the optimal point for quantification of FLK-1^+^ sprouts was day 7 after embedding, as abundant sprouting was observed and both increases and decreases in angiogenesis would be readily measurable (Figures S1B, S1C, and 1E–1H). Using the Cellomics ArrayScan platform, we optimized the neuronal profiling algorithm to objectively quantify the number of FLK-1^+^ sprouts and the total expression of FLK-1 (measured as total fluorescent intensity). This algorithm demonstrated that our assay can detect both increases and decreases in angiogenesis in response to signaling pathway inhibitors (Figures 1F, 1G, and S1C). Visual inspection confirmed automated counts (Figure 1H). The reproducibility of VEGF + DMSO control, as measured by FLK-1^+^ sprout quantification, is shown in Figures S1D and S1E. Treatment with γ-secretase inhibitor (L685,458; referred to hereafter as L685) in the presence of VEGF significantly increased angiogenic sprouting by ≥113.4% (Figures 1E, 1F, and 1H) and total FLK-1 expression by 150.6% compared with VEGF + DMSO control (Figures 1E and 1G), as expected for a NOTCH pathway inhibitor. Treatment with an FLK-1 inhibitor (SU5416) significantly decreased VEGF-induced angiogenic sprouting by ≥82.9% (Figures 1E, 1F, and 1H) and FLK-1 expression by 71% (Figures 1E and 1G). Treatment with L685 or SU5416 in the absence of VEGF resulted in a similar number of FLK-1^+^ sprouts and expression as DMSO controls (p not significant), suggesting that very little angiogenesis occurs in the absence of VEGF (Figures 1E–1H). Given these findings, all inhibitors were added in the presence of VEGF. To characterize the sprouts, we stained them with α-smooth muscle actin (α-SMA) or DLL4, markers for mural cells and tip cells, respectively. VEGF + DMSO-treated EBs showed mural cells surrounding the sprouts (Figure 1I) and high levels of DLL4 in tip cells, whereas DLL4 staining was reduced or absent in the stalk cells (Figure 1J). VEGF + L685-treated EBs showed reduced mural cells (Figure 1I) and decreased DLL4 staining in tip cells (Figure 1J), consistent with previous studies on the effect of NOTCH inhibition on mural cell differentiation and DLL4 expression (Arima et al., 2011). Some mural cells were observed in DMSO- and VEGF + SU5416-treated EBs (Figure 1I), but DLL4 was absent (Figure 1J).

We also demonstrate that our assay, in addition to being sensitive to increases and decreases in vessel sprouting, can also detect morphological changes in vessel shape (Figures S1F and S1G).

### Small-Molecule Kinome Screen for Modulators of Angiogenesis

**Figure 2:**
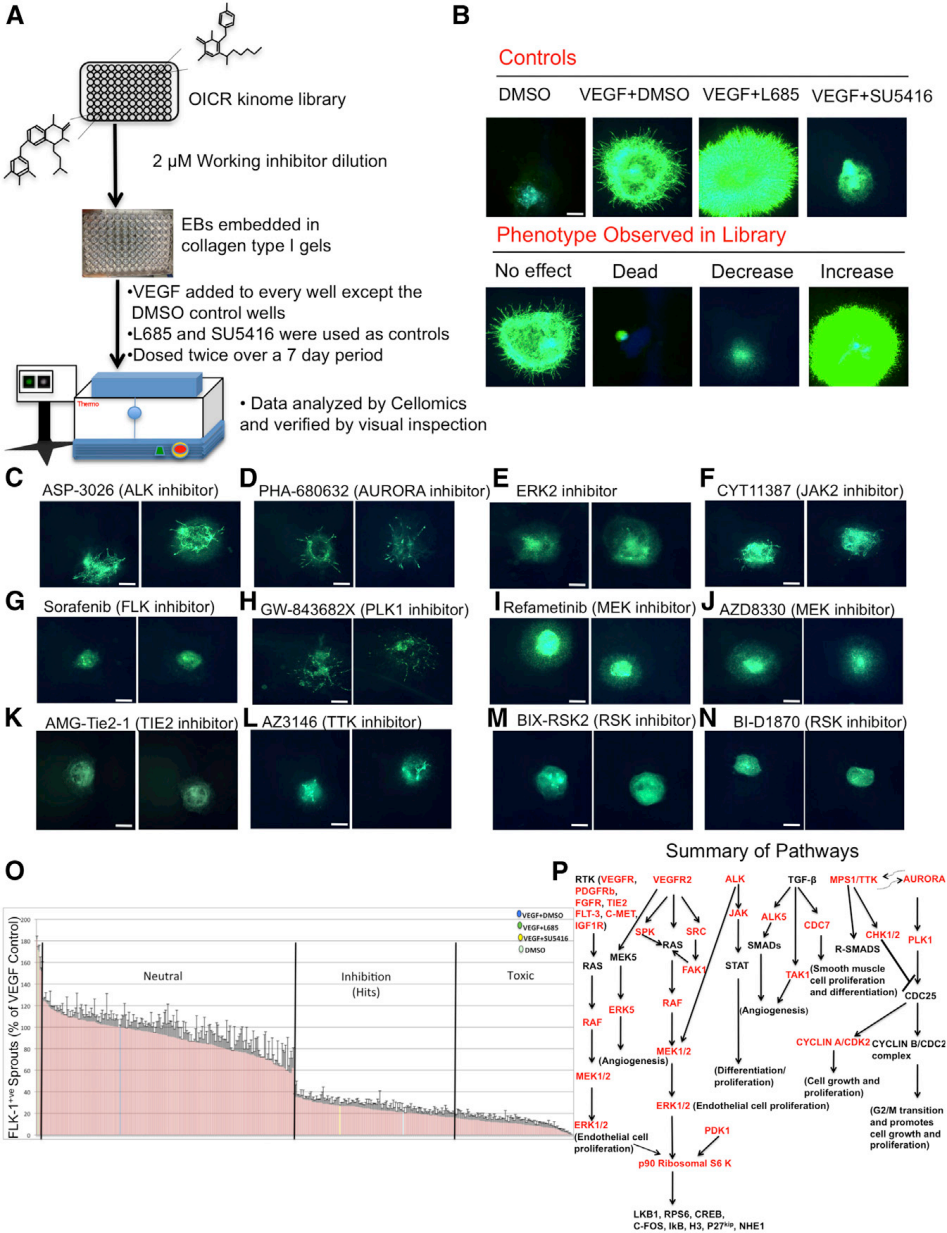
Kinome Small-Molecule Library Screen (A) Schematic of the methodology for screening the small-molecule library. The screen was done blinded. (B) Controls and phenotypes observed in library. Scale bar, 300 μm. (C–N) Representative examples of hits and reproducibility of the phenotype between replicates. Scale bar, 300 μm. (O) Graphical summary of hits. n = 4 technical replicates; data are normalized to VEGF + DMSO controls and expressed as mean ± SEM. (P) All hits in the screen fall into one of the six outlined pathways. Red indicates targets identified in the screen. See also Figures S2 and S3; Tables S1 and S3.

To identify novel modulators of angiogenesis, we used our assay to screen a kinase small-molecule inhibitor library (Figures 2A and 2B) consisting of 480 compounds. Hits were registered as quantitative deviations from VEGF control cultures (Figure S2), since VEGF was also added to every well containing the inhibitors. Automated quantification successfully distinguished neutral events and increases, although distinction between inhibitory and toxic hits had to be confirmed by visual inspection. Representative images of the hits and reproducibility of the phenotypes between replicates are shown in Figures 2C–2N. Graphical representation of the hits is shown in Figure 2O. A hit was considered real/specific if the majority of compounds that were known to inhibit that particular target in the library showed activity. Table 1 lists hits that met these criteria. A few compounds resulted in a NOTCH loss-of-function-like phenotype. For example, a PKC inhibitor resulted in excessive sprouting; however, since the majority of the library compounds targeting PKC showed no effect on angiogenesis, the phenotype was deemed to be an off-target effect and not pursued further (Table S1). Our library screen detected 40 of the 44 compounds that target FLK-1, thus further validating our vascular assay and screening methodology. The majority of hits were validated with dose curves (Figure S3). Our screen identified many kinases with well-established roles in angiogenesis such as FLK-1(Shalaby et al., 1995), TIE2 (Partanen et al., 1996), PDGFRβ (PDGF receptor β) (Rossant and Howard, 2002, Zhang et al., 2009), ALK (anaplastic lymphoma kinase) (Di Paolo et al., 2011), ALK5 (TGFBR1) (Rossant and Howard, 2002), BMK1 (ERK5) (Hayashi et al., 2005, Pi et al., 2005), FGFR (FGF receptor) (Bono et al., 2013), IGFR (insulin-like growth factor receptor) (Bid et al., 2012), MEK1/2 (Giroux et al., 1999), and ERK1/2 (Srinivasan et al., 2009), among others (Table 1). Importantly, we also identified RSK and TTK in the screen; kinases that have not been previously shown to regulate angiogenesis. Our inhibitory hits all fall into one of six signaling pathways (Figure 2P). Criteria for pursuing hits are summarized in Figure S2.

### Compound and Target Hit Validation

**Figure 3:**
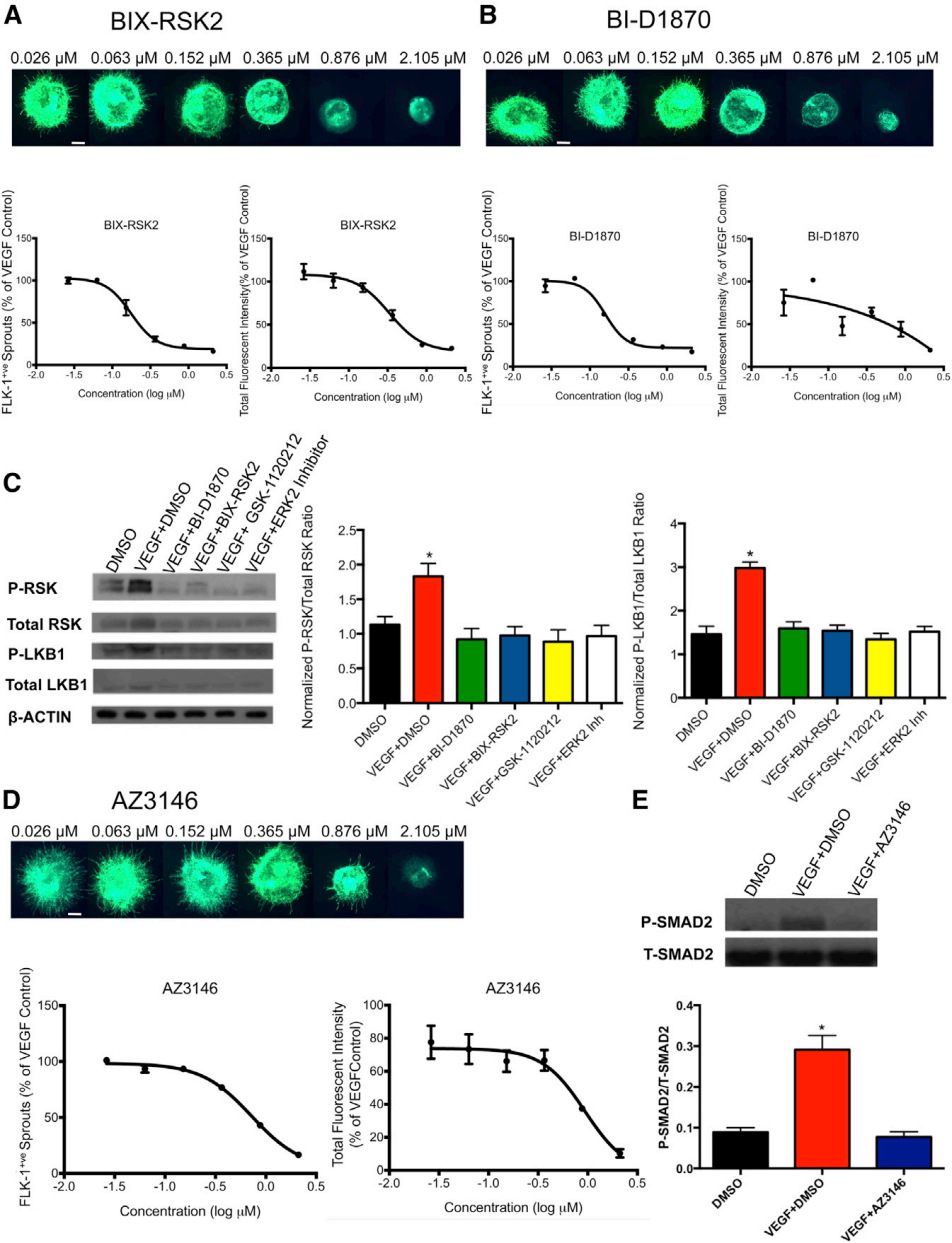
Validation of RSK and TTK Inhibitors in EBs EBs embedded in collagen type I were treated with DMSO, VEGF (50 ng/mL) + DMSO, or VEGF (50 ng/mL) in the presence of inhibitors and dosed twice over a 7-day period. On day 7, EBs were either fixed, imaged and quantified for dose-curve analysis, or lysed in RIPA buffer for western blot analysis. Asterisk denotes statistical significance compared with DMSO control. (A) Dose-curve analysis of BIX-RSK2. (B) Dose-curve analysis of BI-D1870. (C) Western blot analysis showing the effect of 2 μM of various inhibitors on phosphorylated and total levels of LKB1 and RSK. Data were normalized to β-ACTIN. Data are mean ± SEM, n = 5. ^∗^p < 0.01 for normalized P-RSK/total RSK, and ^∗^p < 0.0001 for normalized P-LKB1/total LKB1. (D) Dose-curve analysis of AZ3146. (E) Western blot analysis showing the effect of 2 μM AZ3146 on phosphorylated and total levels of SMAD2. Data are mean ± SEM, n = 6. ^∗^p < 0.0001. For dose-curve analysis (A, B, and D), values were normalized to VEGF + DMSO controls. Drug doses were log transformed. Data are mean ± SEM, n = 4 technical replicates. Scale bars, 300 μm. See also Table S2.

To validate RSK as a hit, we performed a dose-response curve using the inhibitors identified by our screen, BIX-RSK2 (referred to as compound 15 in the study by Fryer et al., 2012) (Figure 3A) and BI-D1870 (Figure 3B). These inhibitors have previously been shown to be selective (Fryer et al., 2012, Sapkota et al., 2007). It is important to note that BI-D1870 at high concentrations (10 μM or above) can have off-target effects, although this inhibitor was shown to be selective when used at 2.5 μM and lower (Roffe et al., 2015, Sapkota et al., 2007). RSKs (RSK1–4) are a family of serine/threonine kinases that share 75%–80% amino acid identity and are activated by the MAPK pathway through a series of phosphorylation events (Anjum and Blenis, 2008). Interestingly, RSK protein levels are elevated in several tumor types (Clark et al., 2005, Smith et al., 2005). Downstream substrates of RSKs include CREB, c-FOS, IκB, LKB1, and RPS6 (Anjum and Blenis, 2008, Romeo et al., 2012). Notably, LKB1 is required for vascular development (Londesborough et al., 2008). We confirmed, through western blot analysis, that BI-D1870 and BIX-RSK2 were targeting the RSK/LKB1 pathway, which is downstream of MEK/ERK. Treatment with VEGF in the presence of 2 μM of either the MEK1/2 inhibitor (GSK-1120212), ERK1/2 inhibitor (ERK2), or RSK inhibitors (BI-D1870, BIX-RSK2) resulted in a significant decrease in p-RSK levels (≥46.6% decrease, p < 0.01) and p-LKB1 (≥46.4% decrease, p < 0.0001) compared with the VEGF + DMSO control (Figure 3C). A schematic of the pathway is depicted in Figure 2P. VEGF + DMSO-treated EBs also showed slightly elevated total RSK and total LKB1 levels (Figure 3C), perhaps due to stabilization of these proteins in response to phosphorylation/activation.

To validate TTK (MPS1) as a bona fide hit, we performed dose-response analysis using AZ3146 (Figure 3D), a TTK inhibitor that was identified in our screen and has been shown to be selective (Hewitt et al., 2010). TTK, a dual-specificity kinase that phosphorylates serine, threonine, and tyrosine residues, is an essential component of the spindle assembly checkpoint and is required for chromosomal alignment during mitosis (Liu and Winey, 2012). TTK expression is elevated in multiple cancers (breast, lung, and gastric cancer) (Liu and Winey, 2012). Downstream targets of TTK include CHK2 (Liu and Winey, 2012) and SMAD2/3 (Zhu et al., 2007). Notably, SMAD2 has been implicated in angiogenesis (Assis et al., 2015, Pen et al., 2008). Our data showed that treatment with VEGF + AZ3146 resulted in a 73.5% decrease (p < 0.0001) in SMAD2 phosphorylation compared with VEGF + DMSO control (Figure 3E).

### The Effect of RSK and TTK Inhibitors on Disruption of Angiogenic Sprouts

**Figure 4:**
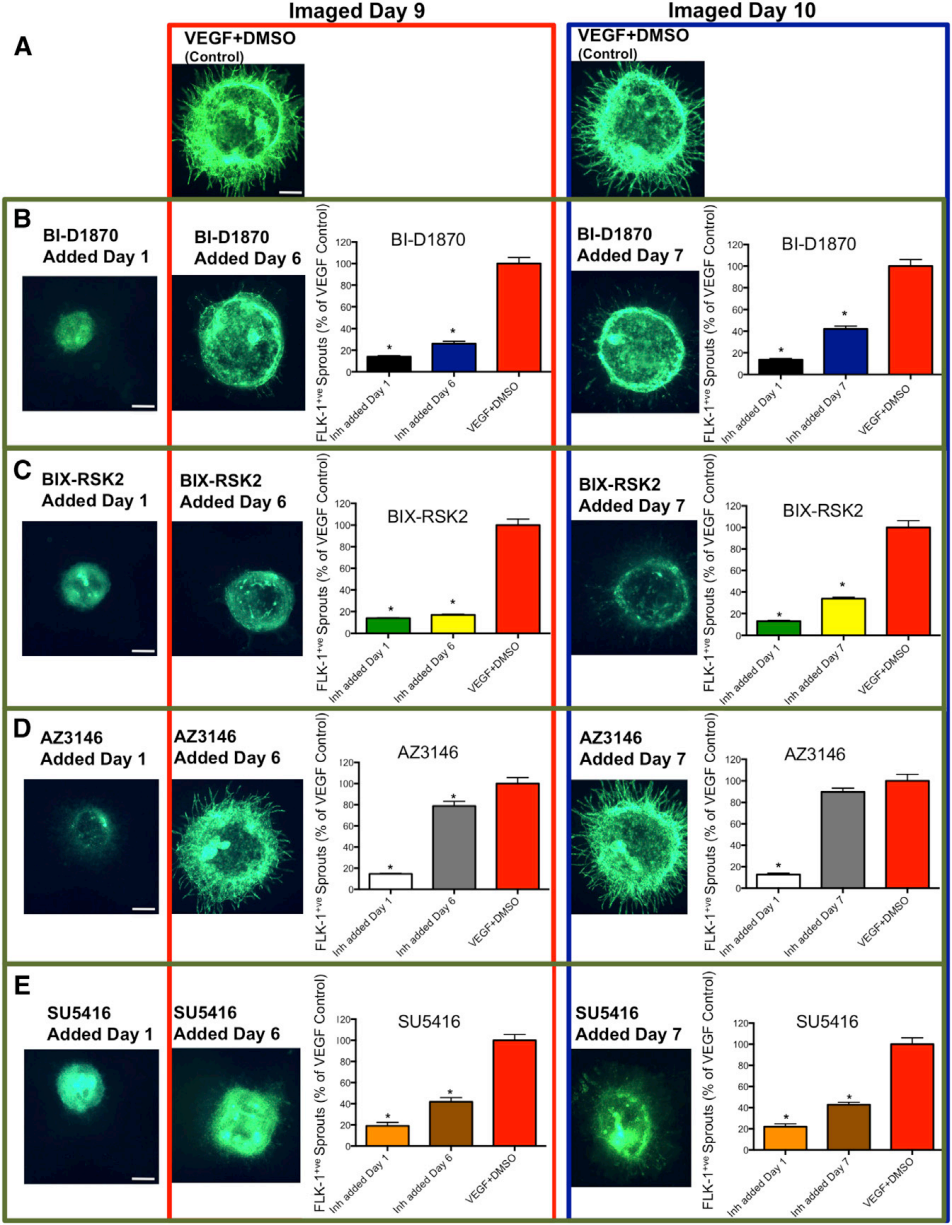
Effect of RSK and TTK Inhibitors on Preformed EB Angiogenic Sprouts RSK and TTK inhibitors (2 μM) or SU5416 (4 μM) were added either at day 1 post embedding, in the presence of VEGF (50 ng/mL), or added on days 6 or 7 post VEGF treatment, also in the presence of 50 ng/mL VEGF, and imaged on days 9 or 10 post embedding, respectively, and the number of FLK-1^+^ sprouts quantified. (A) VEGF + DMSO controls. (B) Effect of BI-D1870. (C) Effect of BIX-RSK2. (D) Effect of AZ3146. (E) Effect of SU5416. Inh, Inhibitors. Data were normalized to VEGF + DMSO controls. Data are mean ± SEM, n ≥ 3. ^∗^p < 0.0001 compared with VEGF + DMSO controls. Scale bars, 300 μm. See also Figure S1B.

To determine whether BI-D1870, BIX-RSK2, and AZ3146 can disrupt preformed angiogenic sprouts, we treated EBs with VEGF for 6 or 7 days before addition of inhibitors, as abundant angiogenesis was observed at these time points (Figures S1B and 1E–1H). BI-D1870, BIX-RSK2, AZ3146, or the known FLK-1 inhibitor SU5416 (Fong et al., 1999) were then added in the presence of VEGF on days 6 or 7. Cultures were maintained for three additional days and fixed. BI-D1870 (Figure 4B), BIX-RSK2 (Figure 4C), and SU5416 (Figure 4E) resulted in significantly reduced FLK-1^+^ sprouts (≥57.2%, p < 0.0001) compared with the VEGF + DMSO controls (Figure 4A). AZ3146 resulted in a significant decrease (21.3%) in FLK-1^+^ sprouts when added on day 6 but not on day 7 (Figure 4D). All four drugs significantly decreased angiogenesis by ≥78.1% (p < 0.0001) when added in the presence of VEGF on day 1 post embedding (Figures 4B–4E).

### The Effect of RSK and TTK Inhibitors on HUVEC Tube Formation and Disruption of Preformed Tubes

**Figure 5:**
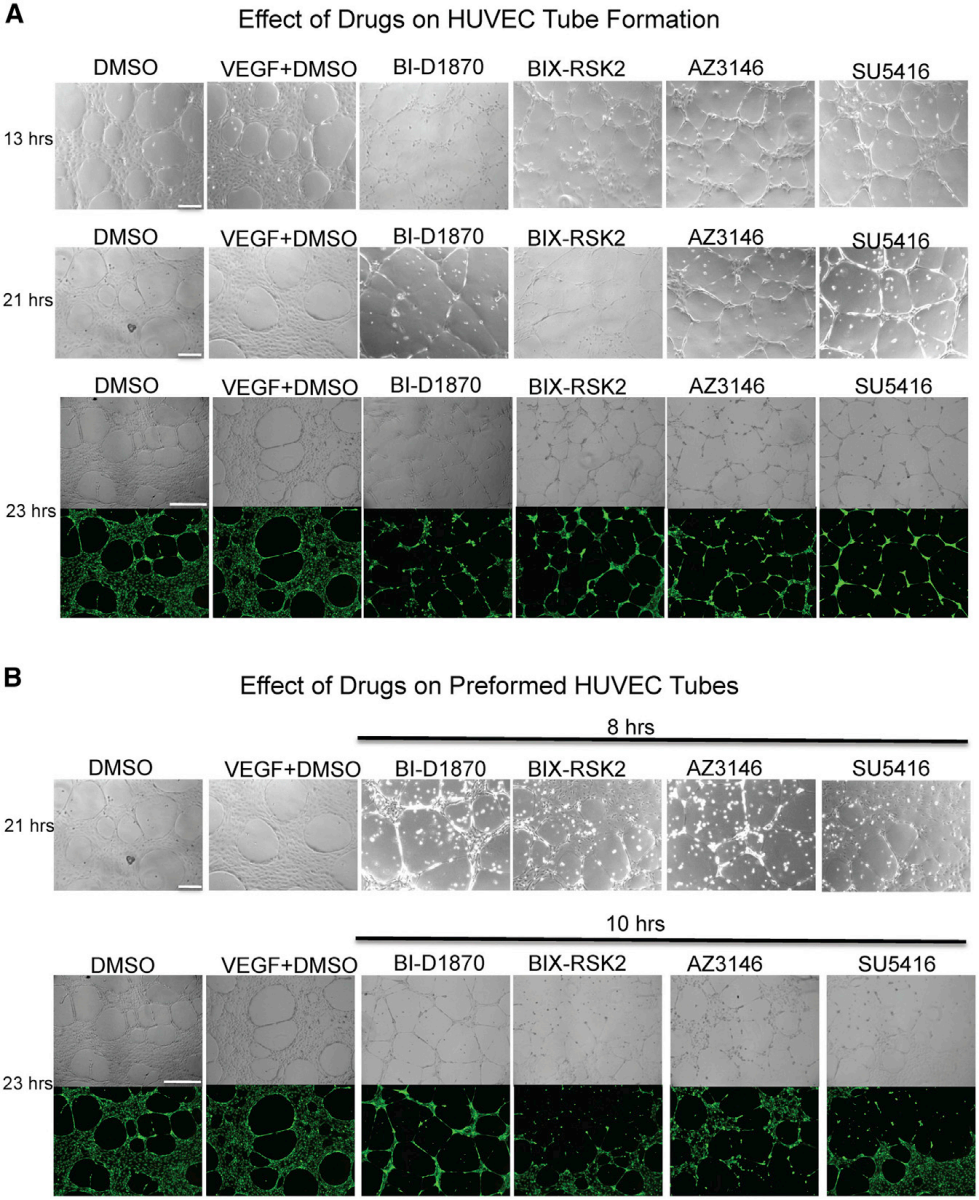
Effect of RSK and TTK Inhibitors on HUVEC Tube Formation and on Preformed HUVEC Tube Networks (A and B) represent two parts of the same experiment and share DMSO and VEGF + DMSO controls. HUVECs were plated on Geltrex, and either (A) immediately treated with complete HUVEC medium containing DMSO, or VEGF (30 ng/mL) + DMSO, or 2 μM BI-D1870, BIX-RSK2, or AZ3146, or 4 μM SU5416, and imaged at 13 hr (scale bar, 200 μm), 21 hr (scale bar, 200 μm) and 23 hr (scale bar, 500 μm) post seeding; or (B) treated 13 hr post plating with 2 μM BI-D1870, BIX-RSK2, or AZ3146, or 4 μM SU5416, and imaged at 21 hr (8 hr drug treatment; scale bar, 200 μm) and 23 hr (10 hr drug treatment; scale bar, 500 μm) post seeding. Calcein-AM was added at the end of the experiment and is shown as green fluorescence in the bottom panels of (A) and (B).

We sought to validate our top hits in a secondary human-relevant cell-based assay, using human umbilical vein endothelial cells (HUVECs). This also allowed us to determine whether inhibition of RSK and TTK, which are expressed in multiple cell types, had a direct effect on endothelial cells. No difference was observed in network morphology between DMSO and VEGF controls, suggesting that complete medium which was not supplemented with VEGF but contained bFGF was sufficient to promote network formation (Figures 5A and 5B). BI-D1870, BIX-RSK2, AZ3146, and SU5416 resulted in disruption of the networks compared with the DMSO or VEGF controls (Figure 5A). Furthermore, when these drugs were added 13 hr post plating, after the networks were already established, they were also able to disrupt the preformed HUVEC tubes (Figure 5B).

### The Effect of RSK and TTK Inhibitors on LL/2 Cells In Vitro and Analysis of In Vivo Exposure to These Drugs

The LL/2 model was chosen to examine the effect of our hits on tumor angiogenesis, as it is a widely used model for studying angiogenesis (Eklund et al., 2013) and is effective in predicting clinical benefit (Chow and Eckhardt, 2007). As BIX-RSK2 is not commercially available, we focused on BI-D1870 and AZ3146 for the remainder of our study. BI-D1870 and AZ3146 had no cytotoxic effects on the LL/2 cells in vitro except at very high doses (Figure S4A). The IC_50_ values showed that BI-D1870 displayed 33.25-fold selectivity, and AZ3146 showed 11.55-fold selectivity for inhibition of EB angiogenic sprouting over LL/2 cell growth inhibition (Table S2). In vivo exposure analysis showed that a dose of 50 mg/kg for BI-D1870 and AZ3146 via intraperitoneal injection resulted in plasma concentrations well above the IC_50_ for inhibiting angiogenesis of the EBs and well below the IC_50_ for having any effect on the LL/2 cells (Table S2). Furthermore, these doses were well tolerated by the mice, with no significant changes in weight or behavior observed (Figure S4B).

### The Effect of the RSK and TTK Inhibitors on Survival, Tumor Growth, and Angiogenesis In Vivo

**Figure 6:**
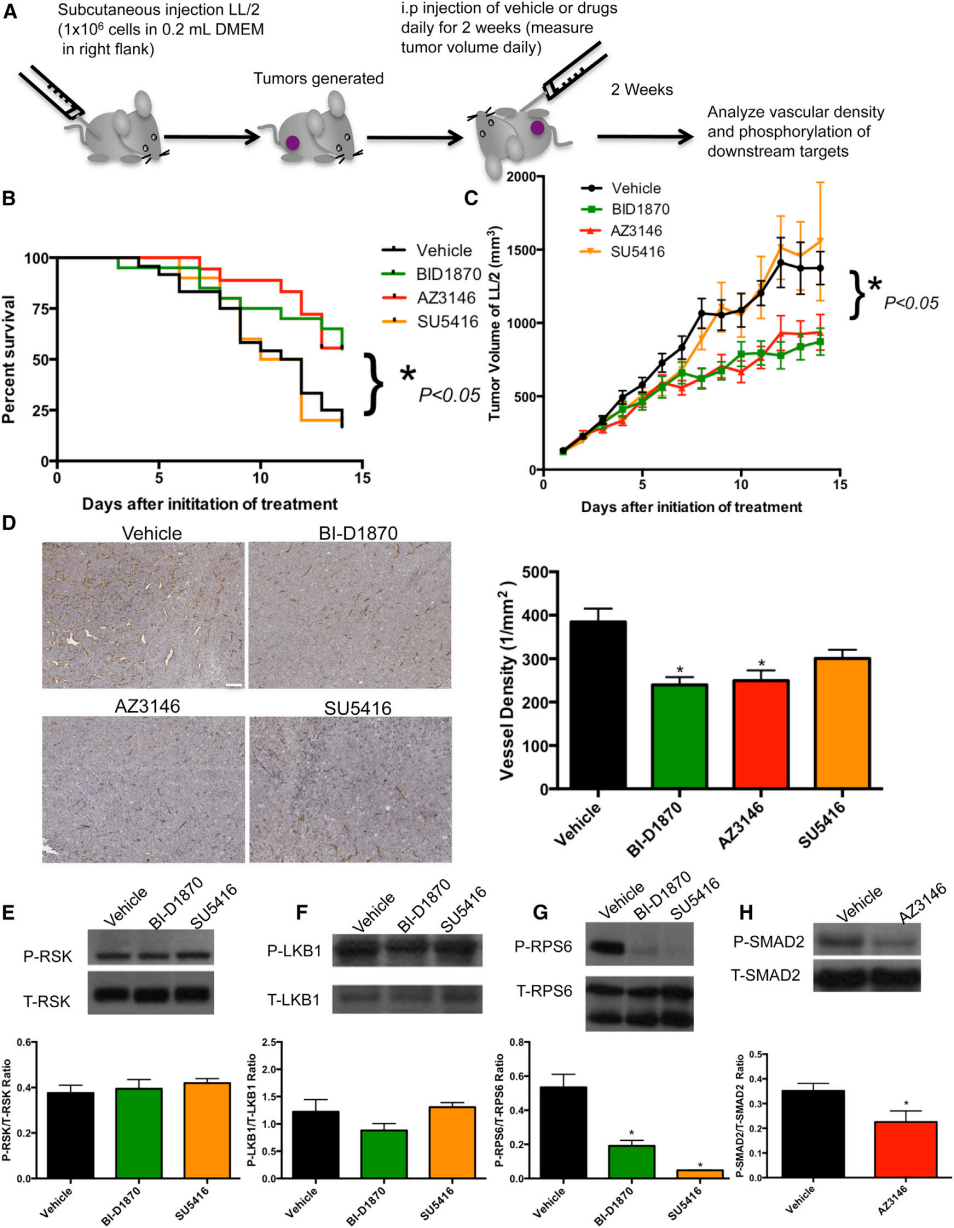
Determination of the Efficacy of BI-D1870 and AZ3146 on a Lewis Lung Tumor Model In Vivo Lewis lung cells (1 × 10^6^) were injected subcutaneously in the right flank of ∼6- to 9-week-old C57Bl/6Crl mice. Mice were randomized into one of four treatment groups: vehicle (n = 24), BI-D1870 (n = 20), AZ3146 (n = 18), and SU5416 (n = 10). Treatments were administered via intraperitoneal (i.p) injection daily for 14 days. Asterisk denotes statistical significance compared with vehicle-treated controls. (A) Schematic of the methodology for the in vivo study. (B) Survival was evaluated using the Kaplan-Meier method. ^∗^p < 0.05. (C) Tumor volume was measured over 14 days. AZ3146 and BI-D1870 resulted in a significant decrease in tumor volume from day 7 and day 8 onward, respectively, compared with vehicle-treated controls. Data are mean ± SEM. ^∗^p < 0.05. (D) Tumors were excised on day 14, fixed, embedded, sectioned, and stained with PECAM-1. Representative images are shown in the left panel (scale bar, 100 μm). Vessel quantification was performed on the majority and in some cases all of the mice/group that survived to the end of the study (day 14 post treatment) and is shown in the graph on the right. Data are mean ± SEM, n = 6 (vehicle), n = 10 (BI-D1870), n = 8 (AZ3146), n = 2 (SU5416). ^∗^p < 0.01. (E–H) Western blot analysis was performed on tumors harvested on day 14 post treatment. (E) Levels of phosphorylated and total RSK. Data are mean ± SEM, p not significant. (F) Levels of phosphorylated and total LKB1. Data are mean ± SEM, p not significant. (G) Levels of phosphorylated and total RPS6. Data are mean ± SEM, ^∗^p < 0.001. (H) Levels of phosphorylated and total SMAD2. Data are mean ± SEM, ^∗^p < 0.05. See also Figure S4 and Table S2.

To test for the efficacy of these compounds on tumor growth and angiogenesis in vivo, we treated LL/2 tumor-bearing mice with vehicle, BI-D1870, AZ3146, or SU5416 via intraperitoneal injections daily for 14 days (Figure 6A). BI-D1870 and AZ3146 significantly improved survival (Figure 6B) and significantly decreased tumor volume from days 8 and 7 onward, respectively, with a ∼37% and ∼32% decrease in tumor volume by day 14 post treatment (Figure 6C, p < 0.05). PECAM-1 staining of tumors excised at day 14 post treatment showed that BI-D1870 and AZ3146 significantly decreased vessel density (∼38% and ∼35%, respectively, p < 0.01) (Figure 6D). Conversely, the well-established antiangiogenic FLK-1 inhibitor SU5416 had no effect on survival (Figure 6B) or tumor volume (Figure 6C), and did not significantly decrease vessel density (Figure 6D). The dose of SU5416 we used has been reported to be the maximum effective tolerated dose for that compound (Fong et al., 1999). Quantification of vessel density in normal host tissue showed no significant difference between vehicle-treated and drug-treated groups (Figure S4C).

To determine the effect of these compounds on signaling, we performed western blot analysis on tumors. BI-D1870 and SU5416 had no effect on RSK (Figure 6E) or LKB1 phosphorylation (Figure 6F) but resulted in a significant decrease (≥64.2%, p < 0.001) in phosphorylation of RPS6 (Figure 6G), a target of RSK (Anjum and Blenis, 2008, Romeo et al., 2012) and the VEGF pathway (Jeong et al., 2014). AZ3146 significantly decreased (35.8%, p < 0.05) phosphorylation of SMAD2, a target of TTK (Zhu et al., 2007) (Figure 6H). Both RPS6 (Hayashi et al., 2005) and SMAD2 (Assis et al., 2015, Pen et al., 2008) have previously been associated with angiogenesis.

## Discussion

We have developed an unbiased, robust, and reproducible three-dimensional EB-based vascular differentiation assay that is amenable to screening for modulators of angiogenesis. The EB-based vascular differentiation assay in collagen matrix offers advantages over the widely used HUVEC/Matrigel assay as well other in vitro angiogenic models in that it uniquely allows the study of both vasculogenesis and angiogenesis (Feraud et al., 2001). The EB assay, unlike the HUVEC assay, models the complex in vivo interactions between endothelial cells and their support cells, which is essential for recapitulating normal vessel formation (Feraud et al., 2001). The assay is sensitive to both increases and decreases in vessel sprouting as well as reading out morphological changes in vessel shape, as exemplified by an additional screen that showed treatment with all-*trans* retinoic acid resulted in the ballooning of vascular sprouts (Figures S1F and S1G).

Previous reports have described ESC-based differentiation in collagen gels to study the developmental events of vasculogenesis and angiogenesis (Feraud et al., 2001, Hermant et al., 2007). However, these were not optimized for the assessment of more fundamental aspects of vessel induction, patterning, and remodeling, nor were these assays standardized into a 96-well plate format suitable for screening. In some previous reports, EB size was not controlled (Feraud et al., 2001, Hermant et al., 2007), which is essential for obtaining the consistency and reproducibility required in a screen. Additionally in these assays, multiple growth factors for vascular induction were used (Feraud et al., 2001, Hermant et al., 2007), or high concentrations of cells were used for EB formation (Jakobsson et al., 2006), which increases variability. Building on the foundation of these studies, we have standardized and simplified the culture system and employed a fluorescent reporter to allow easy monitoring of morphogenesis, thus producing a more robust assay suitable for drug screens in the mouse system. Future studies using human pluripotent cell lines, aided by the advances in genome-editing technologies, will allow the use of more robust reporter lines for endothelial differentiation in the human system.

Our assay was validated using NOTCH and FLK-1 inhibitors, since disruption of these pathways results in visible alterations in angiogenesis (Hellstrom et al., 2007, Shalaby et al., 1995). By screening a small-molecule kinome library we expected a large number of hits, given that the vasculature is very sensitive to signaling pathway disruption. We identified many kinase targets with well-established roles in angiogenesis, including RTKs (VEGFR, PDGFR, FGFR, TIE2, FLT-3, c-MET, and IGF1R) as well as their downstream effectors including RAF, MEK, and ERK, further validating our screen. JAK, ALK, ALK5, and AURORA were also hits and have well-established roles in regulating angiogenesis. It is important to note that despite the fact that our screen is designed to detect both promoters and inhibitors of angiogenesis, all of our validated hits inhibit angiogenesis. It is possible that the NOTCH pathway may be unique in causing excessive sprouting. Interestingly, inhibition of ALK1 has also been shown to lead to excessive angiogenic sprouting, which was attributed to cooperation of ALK1 with the NOTCH pathway (Kerr et al., 2015). An additional screen of a more broad-based library similarly showed that only NOTCH inhibitors resulted in excessive angiogenic sprouting (data not shown). Screening of this second library showed that our assay is sensitive to phenotypic changes that were measurable beyond just increases or decreases in the number of FLK-1^+^ sprouts (i.e., retinoids had no major effect on sprout number but caused morphological changes in vessel shape). This suggests that the complete landscape of target space that can be explored with this assay is still to be determined.

We identified RSK and TTK as angiogenic modulators. We showed that treatment of EBs or HUVECS with BI-D1870 and BIX-RSK2, the selective RSK inhibitors, or with AZ3146, the selective TTK inhibitor, inhibited angiogenic sprouts in EBs and network formation in HUVECs, and disrupted the preformed HUVEC tubes and the preformed EB angiogenic sprouts. It is important to note that these inhibitors disrupted network formation in HUVECs induced by bFGF without VEGF supplementation, suggesting that they are downstream of multiple proangiogenic pathways. Western blot analysis of EBs showed that TTK and RSK inhibitors resulted in a significant decrease in phosphorylation of the downstream targets, SMAD2 and LKB1, respectively, in association with the observed decrease in angiogenesis. A previous report has suggested the involvement of RSK in angiogenesis, although no direct evidence was provided (Hayashi et al., 2005). Our study provides direct proof that RSK and TTK regulate angiogenesis.

Future studies using genetic approaches involving the generation of ESC lines with inducible gene knockout of RSK and TTK need to be performed to further validate our hits.

To determine whether the inhibition of RSK and TTK inhibits neovascularization in vivo, we gave mice LL/2 tumor grafts. Both BI-D1870 and AZ3146, used at doses determined to be non-toxic to animals, significantly improved survival, inhibited tumor growth, and decreased vascular density. In contrast, SU5416 had no effect on survival. In agreement with a recent study (Ogawara et al., 2014), SU5416 did not affect LL/2 tumor graft growth nor significantly decreased vessel density. In contrast to Ogawara's and our studies, there is a previous report showing that SU5416 significantly inhibited tumor angiogenesis and metastasis of an LL/2 model (Cuneo et al., 2007). This discrepancy may be due to the fact that Cuneo et al. (2007) began treatment with SU5416 either immediately or 1 hr after injecting LL/2 cells, whereas in our study and that of Ogawara et al. (2014), we began treatment once tumors reached a minimal volume of 100 mm^3^. Therefore, in the LL/2 model SU5416 may be effective in inhibiting host vessels from infiltrating the tumor, but ineffective once the tumor is well vascularized. Ogawara et al. (2014) showed that SU5416 significantly reduced tumor growth in B16 and C-26 with no effect on LL/2 tumor grafts. This was attributed to high levels of VEGF within B16 and C-26 tumors, compared with LL/2 tumors, suggesting that VEGF does not play a major role in the angiogenesis of LL/2 tumors; instead, other proangiogenic factors, such as bFGF, are responsible for angiogenesis/tumor growth in LL/2. Other studies showed that inhibition of VEGF in tumors can lead to upregulation of bFGF and other proangiogenic factors (Lu and Bergers, 2013) to overcome VEGF inhibition. Interestingly, RSK is a downstream target of bFGF (Czaplinska et al., 2014) and VEGF (Seko et al., 1998), which constitute two major proangiogenic pathways involved in tumor growth (Lu and Bergers, 2013).

Although many of the signaling events involved in developmental angiogenesis are also involved in tumor angiogenesis, there are distinct differences between these two processes, which lead to dysfunctionality of the tumor vasculature. In the case of tumors, tissue disorganization, high enzymatic activity, overproduction of growth factors and extracellular matrix components, and changes in pH and oxygen in the tumor environment lead to detachment of pericytes, leakiness of vessels, and loss of vascular integrity (Jin and Jakobsson, 2012). This could explain why AZ3146 and BI-D1870 resulted in a significant decrease in vessel density in the tumor but had no effect on the vessel density in normal host tissue. This, along with the lack of change in body weight and behavior, indicates that these drugs at the doses used were not toxic.

We also investigated the effect of AZ3146 and BI-D1870 on the downstream phosphorylation of targets in tumors. AZ3146 significantly decreased SMAD2 phosphorylation in tumors. SMAD2 can positively regulate VEGF release in various tumor cell lines (Seystahl et al., 2015) and plays a role in angiogenesis (Assis et al., 2015, Pen et al., 2008). BI-D1870 had no effect on LKB1 phosphorylation in tumors, unlike EBs. This finding is not surprising, as LKB1 has been shown to promote physiological angiogenesis (Londesborough et al., 2008), whereas in cancer cells it acts as a tumor suppressor and inhibits angiogenesis (Zhuang et al., 2006). However, treatment with BI-D1870 significantly decreased RPS6 phosphorylation in tumors. Interestingly, decreased phosphorylation of RPS6 has been correlated with decreased tumor angiogenesis (Hayashi et al., 2005).

In summary, we have developed and validated a robust vascular differentiation assay from ESCs that can be used to screen for modulators of angiogenesis. This in vitro assay identified RSK and TTK as components of vascular signaling pathways. Inhibition of these pathways in vivo in an LL/2 tumor mouse model increased survival, inhibited tumor growth, and decreased angiogenesis associated with decreased RPS6 and SMAD2 phosphorylation. Extension of this screening approach to a broader spectrum of molecular targets may provide new insights into the regulation of vascular development and uncover potential new targets for the therapeutic modulation of angiogenesis.

## Experimental Procedures

Reagents were purchased from Invitrogen unless otherwise specified.

### Cell Lines and Culture

*Flk1-eGFP* mouse ESCs (Ema et al., 2006) were cultured on mitotically inactivated mouse embryonic fibroblasts (MEFs) in ESC media (ES-DMEM) consisting of high-glucose DMEM, 2 mM GlutaMax, 0.15 mM 1-thioglycerol (Sigma-Aldrich), 0.1 mM nonessential amino acids, 1 mM sodium pyruvate, 1,000 U/mL LIF (Chemicon), 50 U/mL penicillin-streptomycin, and 15% ESC-qualified fetal bovine serum (FBS).

HUVECs were cultured in Medium 200PRF supplemented with the LSGS Kit.

LL/2 cells (ATCC) were maintained in high-glucose DMEM (ATCC) supplemented with 10% FBS.

### Optimized Vascular Differentiation Assay and Kinome Inhibitor Screen

MEF-depleted *Flk1-eGFP* cells (10^4^ cells/mL) were aggregated in suspension (20-μL hanging drops) for 4 days to form EBs in differentiation media consisting of Iscove's medium (IMDM, Sigma-Aldrich), 1.6 mM GlutaMax, 0.081 mM nonessential amino acids, 0.081 mM 2-mercaptoethanol (Sigma-Aldrich), 10% FBS, 50 U/mL penicillin-streptomycin, and 50 μg/mL ascorbic acid (Sigma-Aldrich). On day 4, EBs were embedded in 2 mg/mL rat tail collagen type I (BD Biosciences) gels in the individual wells of a 96-well plate and incubated at 37°C. The following day differentiation media alone or containing DMSO or VEGF (50 ng/mL, R&D Systems), or VEGF (50 ng/mL) in the presence of other growth factors (100 ng/mL bFGF [R&D]; 10 ng/mL IL-6; and/or 2 U/mL EPO [provided by the Keller laboratory]), or 50 ng/mL VEGF in the presence of 5 μM L685,458 (EMD Biosciences) or 4 μM SU5416 (Sigma-Aldrich) or 2 μM kinase library inhibitors (provided by Ontario Institute for Cancer Research, see Table S3 for the compound list) were added and the media replenished on day 3. The EBs were fixed in 4% formaldehyde (Polysciences) on day 7 (unless otherwise noted) and the number of FLK-1^+^ sprouts and total fluorescent intensity were measured using the Cellomics VTI (Zeiss Axio Observer.Z1 microscope, ORCA-ER camera) (Thermo Fisher) platform with a modified neuronal profiling algorithm (see Supplemental Experimental Procedures).

Immunohistochemistry of EBs in collagen gels, dose-curve validation of hits, western blot analysis, HUVEC tube formation assay and disruption of preformed tubes, cytotoxicity assay, and in vivo exposure analysis are described in detail in the Supplemental Experimental Procedures.

### Mouse Tumor Grafts and In Vivo Drug Studies

All procedures involving animals were performed in agreement with the Canadian Council for Animal Care (CCAC) guidelines at the Toronto Center for Phenogenomics. Female C57BL/6NCrl mice (∼6–9 weeks old) were injected subcutaneously in the right flank with 1 × 10^6^ LL/2 cells in 0.2 mL of serum-free DMEM. Tumor volume was calculated using the formula: (length × width)^2^ × 0.5. Mice were randomized into one of four treatment groups where the average tumor volume ± SEM per group at the initiation of treatment was as follows: vehicle (129.8 ± 6.6 mm^3^, n = 24), BI-D1870 (122 ± 7.6 mm^3^, n = 20), AZ3146 (128.6 ± 10.5 mm^3^, n = 18), and SU5416 (124.1 ± 6.8 mm^3^, n = 10). Treatments were administered via intraperitoneal injection daily for 14 days or until animal endpoint. BI-D1870 and AZ3146 were administered at 50 mg/kg, and SU5416 at 25 mg/kg. Tumors were calipered daily for volume assessment. The experiment was staggered to allow proper handling/monitoring of mice. Mice were euthanized if tumors reached endpoint as outlined by the CCAC (ulcerated tumor, tumor volume ≥1.7 cm^3^, or tumor mass = 5% of body weight), or if they displayed poor health. Otherwise mice were euthanized after the last drug dose on day 14. Tumors were excised, and a portion was fixed in 10% formalin and embedded in paraffin. The remainder was cut into pieces and snap-frozen.

### Immunohistochemistry and Microvascular Density Quantification of Tumors and Adjacent Normal Tissue

Tumor sections (5 μm), two sections per tumor, were stained with anti-PECAM-1 (M-20) (Santa Cruz Biotechnology). Bound antibody was detected with ImmPRESS (Peroxidase) Polymer anti-rabbit immunoglobulin reagent (Vector Laboratories) and visualized using ImmPACT DAB peroxidase (HRP) substrate (Vector Labs). Mayer's hematoxylin (Sigma-Aldrich) was used as a counterstain. Slides were digitized using an Aperio ScanScope XT scanner (Leica), and computer-aided image analysis was performed and manually checked for quality assurance. Regions of interest were identified with an algorithm that distinguishes tumor from stroma and (peri-)necrotic regions. The vessel density within the tumor region, as well as in the adjacent normal tissue, was quantified using the Definiens Tissue Studio software platform (Definiens). Quantification was done while blinded to the treatment groups.

### Statistical Analysis

Statistical analysis was performed using GraphPad Prism, via unpaired Student's t tests, or one-way ANOVA followed by Newman-Keuls post tests. Data are presented as mean ± SEM. Survival was evaluated by the Kaplan-Meier method. n represents the number of independent experiments, unless otherwise noted. p < 0.05 was considered statistically significant.

## Figures and Tables

**Table 1:** Hits Identified in Vascular Screen

Categories	Drug Target	Number of Inhibitors that Hit Target/Total Number of Inhibitors of that Target in Library	Number of Dose Curves (Validation)	References (Angiogenesis)
Cell surface receptors	ALK	3/4		Di Paolo et al., 2011, Safina et al., 2007
	ALK5	8/10	1	Rossant and Howard, 2002
	c-MET	13/20	1	Lu and Bergers, 2013
	FGFR	11/13	2	Bono et al., 2013
	FLK1	40/44	2	Ferrara et al., 2005, Shalaby et al., 1995
	FLT1/FLT4/KIT	6/6	1	Sleijfer et al., 2009
	FLT3/FLK2, SYK	22/24, 4/4	2	Kazerounian et al., 2011
	IGFR	4/7	3	Bid et al., 2012
	PDGFRβ	26/36		Zhang et al., 2009
	TIE2	5/5	1	Partanen et al., 1996
MAPK pathway	ERK2	4/6	1	Srinivasan et al., 2009
	ERK5	1/2	1	Hayashi et al., 2005, Pi et al., 2005
	FAK	3/4	1	Tavora et al., 2010
	MEK1/2	12/15	2	Giroux et al., 1999
	PDK1	3/5	1	Tawaramoto et al., 2012
	RAF	11/17	2	Wimmer et al., 2012
	SRC/FYN/ABL/LCK	5/7	4	Schenone et al., 2007
	TAK1 (MAP3K7)	3/3		Jadrich et al., 2006
	p90 ribosomal S6K (RSK)	2/2	2	
JAK/STAT pathway	JAK	13/20	1	Xin et al., 2011
Cell-cycle regulators	AURORA	19/21		Romain et al., 2014
	CDC7	1/1		Shi et al., 2012
	CDK2/CYCLIN A	13/20		Chen et al., 2000
	MPS1 (TTK)	1/1	1	
	CHK1/2	8/11	2	
	PLK1	6/8	2	Gomes et al., 2013
	SPK1	2/3	1	Duan et al., 2007

See also Figure S3.

## Supplemental Information

Document S1. Supplemental Experimental Procedures, Figures S1–S4, and Tables S1 and S2 Document S1. Supplemental Experimental Procedures, Figures S1–S4, and Tables S1 and S2

Table S3. List of Library Compounds and Their Targets, Related to Figure 2 Table S3. List of Library Compounds and Their Targets, Related to Figure 2

Document S2. Article plus Supplemental Information Document S2. Article plus Supplemental Information
