# Supplementary material for: Identification of RSK and TTK as Modulators of Blood Vessel Morphogenesis Using an Embryonic Stem Cell-Based Vascular Differentiation Assay
Source: Stem Cell Reports. 2016 Sep 8;7(4):787–801. doi: 10.1016/j.stemcr.2016.08.004 (PMC5063585; doi:10.1016/j.stemcr.2016.08.004)
Supplement: Document S1. Supplemental Experimental Procedures, Figures S1–S4, and Tables S1 and S2 [file mmc1.pdf]

Stem Cell Reports, Volume *7*

Supplemental Information

Identification of RSK and TTK as Modulators of Blood Vessel Morphogenesis Using an Embryonic Stem Cell-Based Vascular Differentiation Assay

Lamis Hammoud, Jessica R. Adams, Amanda J. Loch, Richard C. Marcellus, David E. Uehling, Ahmed Aman, Christopher Fladd, Trevor D. McKee, Christine E.B. Jo, Rima Al-Awar, Sean E. Egan, and Janet Rossant

Supplemental Figures

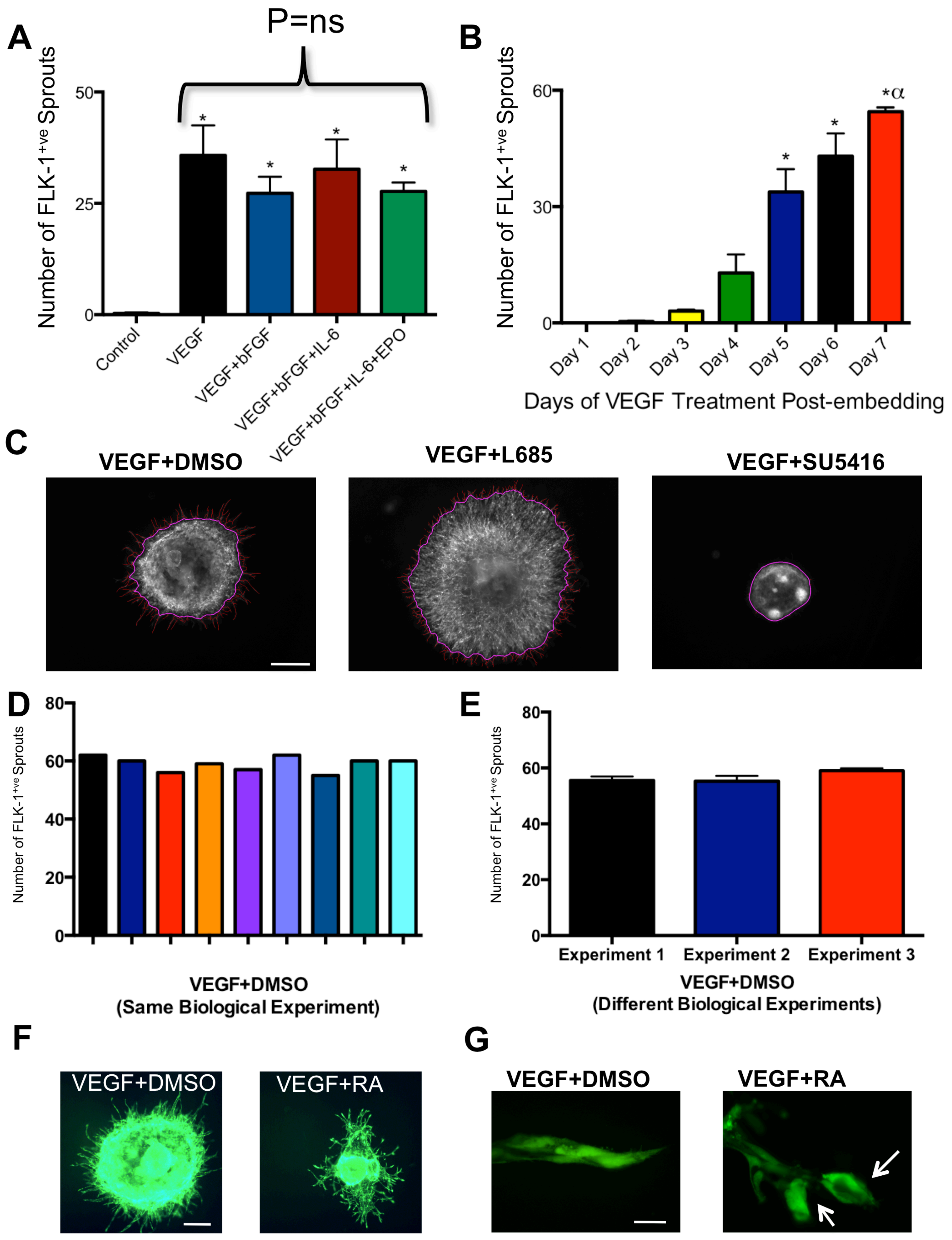

Figure S1

Hits Analysis

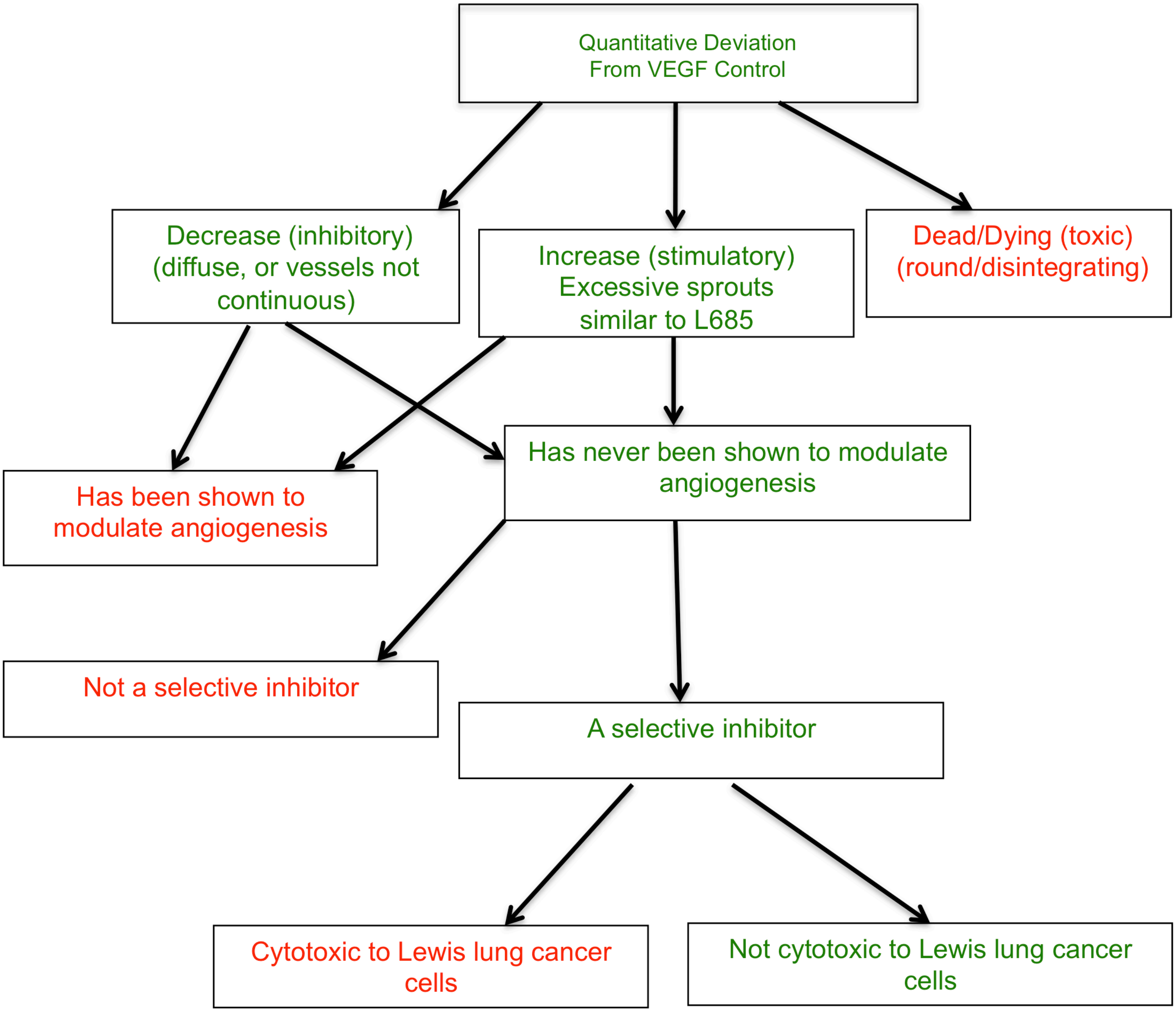

Figure S2

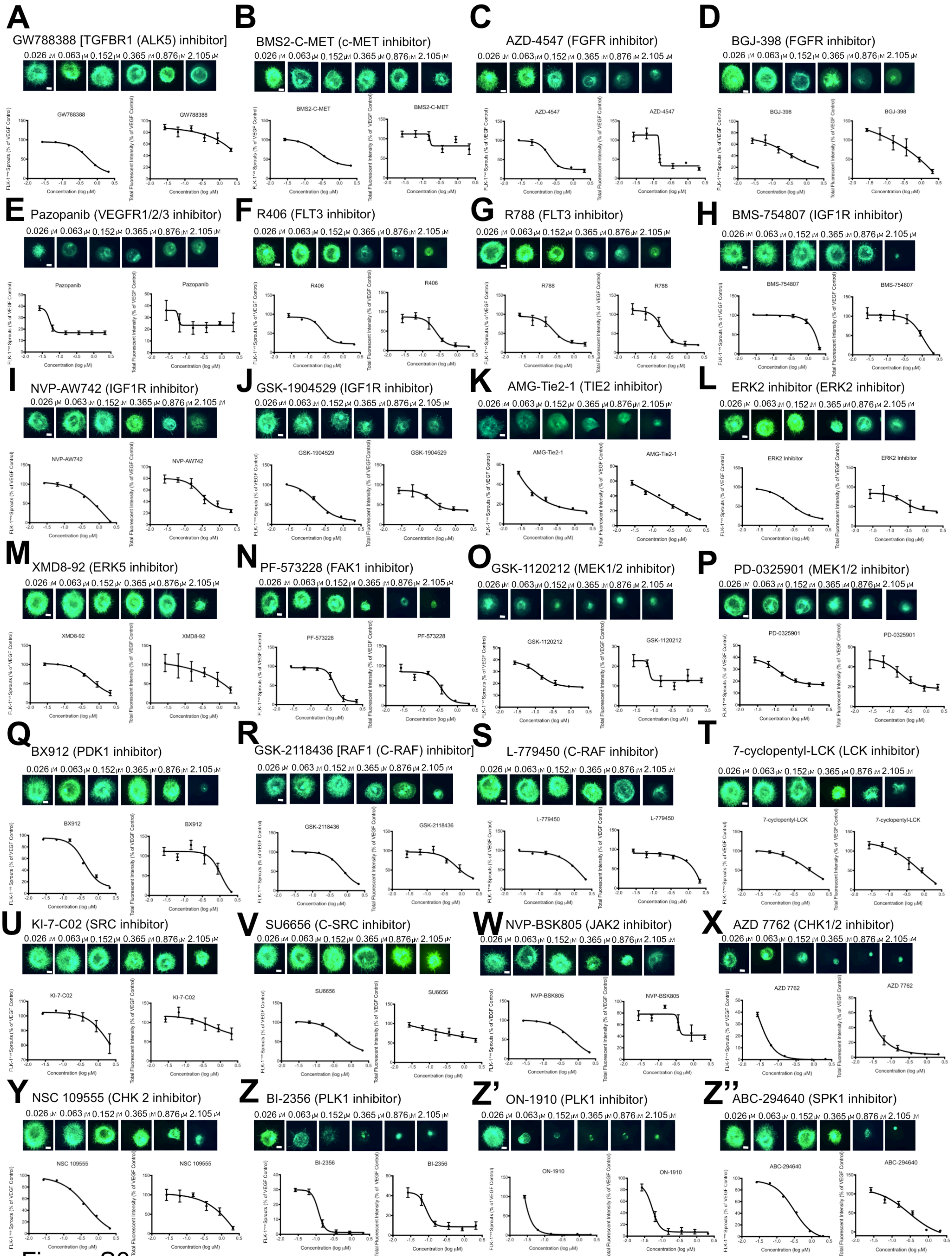
A GW788388 [TGFBR1 (ALK5) inhibitor]
B BMS2-C-MET (c-MET inhibitor)
C AZD-4547 (FGFR inhibitor)
D BGJ-398 (FGFR inhibitor)
E Pazopanib (VEGFR1/2/3 inhibitor)
F R406 (FLT3 inhibitor)
G R788 (FLT3 inhibitor)
H BMS-754807 (IGF1R inhibitor)
I NVP-AW742 (IGF1R inhibitor)
J GSK-1904529 (IGF1R inhibitor)
K AMG-Tie2-1 (TIE2 inhibitor)
L ERK2 inhibitor (ERK2 inhibitor)
M XMD8-92 (ERK5 inhibitor)
N PF-573228 (FAK1 inhibitor)
O GSK-1120212 (MEK1/2 inhibitor)
P PD-0325901 (MEK1/2 inhibitor)
Q BX912 (PDK1 inhibitor)
R GSK-2118436 [RAF1 (C-RAF) inhibitor]
S L-779450 (C-RAF inhibitor)
T 7-cyclopentyl-LCK (LCK inhibitor)
U KI-7-C02 (SRC inhibitor)
V SU6656 (C-SRC inhibitor)
W NVP-BSK805 (JAK2 inhibitor)
X AZD 7762 (CHK1/2 inhibitor)
Y NSC 109555 (CHK 2 inhibitor)
Z BI-2356 (PLK1 inhibitor)
Z' ON-1910 (PLK1 inhibitor)
Z'' ABC-294640 (SPK1 inhibitor)
0.026 μM 0.063 μM 0.152 μM 0.365 μM 0.876 μM 2.105 μM
FLK-1+ve Sprouts (% of VEGF Control)
Total Fluorescent Intensity (% of VEGF Control)
Concentration (log μM)

Figure S3

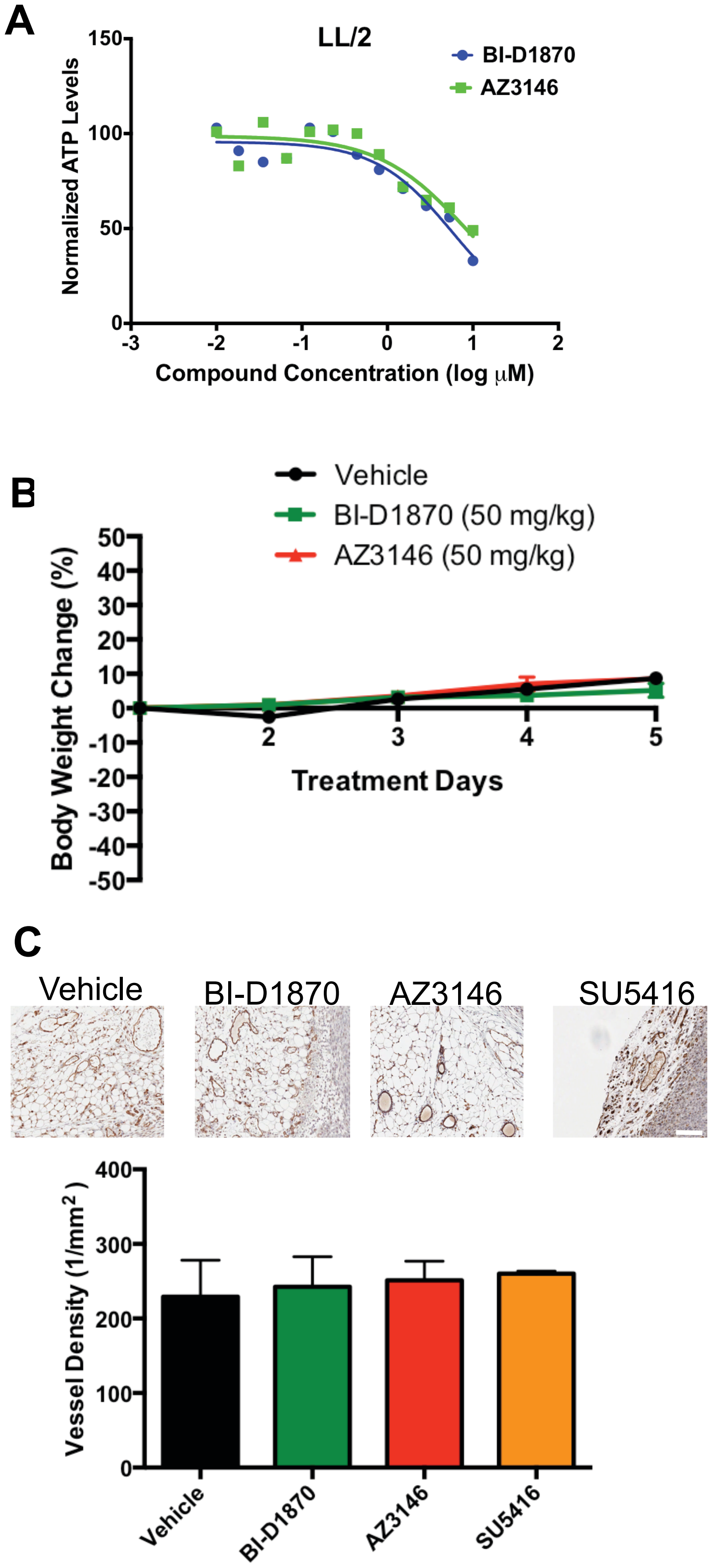
A
LL/2
BI-D1870
AZ3146
Normalized ATP Levels
Compound Concentration (log µM)
B
Vehicle
BI-D1870 (50 mg/kg)
AZ3146 (50 mg/kg)
Body Weight Change (%)
Treatment Days
C
Vehicle
BI-D1870
AZ3146
SU5416
Vessel Density (1/mm²)
Vehicle
BI-D1870
AZ3146
SU5416

Figure S4

Supplemental Figure Legends

Figure S1 (related to Figure 1). Effect of growth factor treatment on EB sprouting following embedding in collagen type I gels, determination of the optimal time point for sprout quantification, automated quantification using Cellomics Neuronal Profiling BioApplication, reproducibility of the VEGF+DMSO control in the same and different biological experiments, and sensitivity of our assay for detection of morphological changes in vessel shape. *Flk1-eGFP* ESCs were aggregated in suspension for 4 days as hanging drops and then embedded in a collagen type I matrix. (A) EBs were grown in differentiation media alone (control) or containing VEGF (50 ng/mL), or containing VEGF (50 ng/mL) in the presence of other growth factors (100 ng/mL bFGF; 10 ng/mL IL-6; 2 U/mL EPO). The number of FLK-1 positive sprouts were quantified on day 6 post-embedding. Data are mean ± SEM, N≥3 $P<0.0001$. Star (*) denotes statistical significance compared to control. P=ns for VEGF vs other growth factors. (B) EBs were grown in media containing VEGF (50 ng/ml) and FLK-1 positive sprouts were quantified each day for 7 days post-embedding. N=3, data are mean ± SEM. Star (*) denotes statistical significance compared to day 1,2,3 and 4, and α denotes statistical significance compared to day 5, and 6. (C) Representative images acquired using the Cellomics VTI platform and quantified using the Neuronal Profiling BioApplication. Red denotes FLK-1 positive sprouts quantified by the algorithm. (D-E) EBs were grown in media containing VEGF (50 ng/ml)+DMSO and FLK-1 positive sprouts were quantified on day 7 post-embedding. (D) Reproducibility of the VEGF+DMSO control between technical replicates. (E) Reproducibility of the VEGF+DMSO control between independent experiments (biological replicates). Data are mean ± SEM, N=3, P=ns. (F-G) Treatment with 2 μM all-trans retinoic acid (RA) resulted in ballooning of the vascular sprouts. Scale bars were 300 μm and 25 μm for C and D respectively. White arrows point to ballooned structures.

Figure S2 (related to Figure 2). Hit analysis chart. For a hit to be worth pursing, the compound should be selective and have established potency (<50 nM) against the kinase target of interest, as well as have novelty with respect to angiogenesis as an indicated use. Additionally, the compound must not have been reported to be cytotoxic to Lewis lung carcinoma cell line (our tumor model of choice). Green indicates worth pursuing further, red indicates not worth pursing further.

Figure S3 (related to Figure 2 and Table 1). Dose curve analysis of ALK5, c-MET, FGFR, VEGFR1/2/3, FLT3, IGF1R, TIE2, ERK2, ERK5, FAK1, MEK1/2, PDK1, RAF1/c-RAF, LCK, SRC, JAK2, CHK1/2, PLK1 and SPK1 inhibition. *Flk1-eGFP* ESCs were aggregated in suspension for 4 days as hanging drops and then embedded in a collagen type I matrix, one EB per well of 96 well plate. The following day EBs were treated with DMSO, VEGF (50 ng/ml) in the presence of DMSO, or VEGF (50 ng/ml) in the presence of varying concentrations of compounds (0.026 μM-2.1 μM), and dosed twice over a 7 day period. On day 7 post-embedding, EBs were fixed, imaged and the number of FLK-1 positive sprouts and total fluorescent intensity were quantified. Values were normalized to VEGF control. Drug doses were Log transformed. Data are mean ± SEM, N=4 technical replicates. For A-Z", scale bar, 300 μm. (A) Dose curve analysis of GW788388 (ALK5 inhibitor). (B) Dose curve analysis of BMS2-C-MET (c-MET inhibitor). (C) Dose curve analysis of AZD-4547 (FGFR inhibitor). (D) Dose curve analysis of BGJ-398 (FGFR inhibitor). (E) Dose curve analysis of Pazopanib (VEGFR1/2/3 inhibitor). (F) Dose curve analysis of R406 (FLT3 inhibitor). (G) Dose curve analysis of R788 (FLT3 inhibitor). (H) Dose curve analysis of BMS-754807 (IGF1R inhibitor). (I) Dose curve analysis of NVP-AW742 (IGF1R inhibitor). (J) Dose curve analysis of GSK-1904529 (IGF1R inhibitor). (K) Dose curve analysis of AMG-Tie2-1 (TIE2 inhibitor). (L) Dose curve analysis of ERK2 inhibitor (ERK2 inhibitor). (M) Dose curve analysis of XMD8-92 (ERK5 inhibitor). (N) Dose curve analysis of PF-573228 (FAK1 inhibitor). (O) Dose curve analysis of GSK-1120212 (MEK1/2 inhibitor). (P) Dose curve analysis of PD-0325901 (MEK1/2 inhibitor). (Q) Dose curve analysis of BX912 (PDK1 inhibitor). (R) Dose curve analysis of GSK-2118436 (RAF1/c-RAF inhibitor). (S) Dose curve analysis of L-779450 (c-RAF inhibitor). (T) Dose curve analysis of 7-cyclopentyl-LCK (LCK inhibitor). (U) Dose curve analysis of KI-7-C02 (SRC inhibitor). (V) Dose curve analysis of SU6656 (c-SRC inhibitor). (W) Dose curve analysis of NVP-BSK805 (JAK2 inhibitor). (X) Dose curve analysis of AZD 7762 (CHK1/2 inhibitor). (Y) Dose curve analysis of NSC 109555 (CHK2 inhibitor). (Z) Dose curve

analysis of BI-2356 (PLK1 inhibitor). (Z') Dose curve analysis of ON-1910 (PLK1 inhibitor). (Z") Dose curve analysis of ABC-294640 (SPK1 inhibitor).

Figure S4 (related to Figure 6 and Table S2). Determination of whether BI-D1870 or AZ3146 have cytotoxic effects on Lewis lung tumor cells *in vitro* and whether these drugs have toxic effects *in vivo.* Lewis lung cells were plated in 96 well plates and treated with increasing doses (0-10 μM) of BI-D1870 or AZ3146, 24 hours post-plating. ATP quantification, measured as chemiluminescence was performed 72 hours post-plating and data were normalized to DMSO controls. Drug doses were Log transformed. (B) During *in vivo* exposure analysis, mice were treated with either vehicle, 50 mg/kg BI-D1870 or 50 mg/kg AZ3146 via intraperitoneal (i.p) injection for 5 days, no significant change in body weight was observed between vehicle and drug treated mice. Data are mean± SEM, N=3 mice/group, *P*=ns. (C) PECAM-1 staining and quantification of the vessel density in the normal tissue, adjacent to the tumors, showed no significant difference between vehicle treated and drug treated groups. Scale bar, 100 μm. Data are mean ± SEM, N=6 (vehicle), N=10 (BI-D1870), N=8 (AZ3146), N=2 (SU5416), *P*=ns.

Supplemental Tables

Table S1 (related to Figure 2). Inhibitors with off-target effects.

Compound	Target	Reason considered off-target
TX-1918	eEF2K	These eF2K inhibitors are known to have off-target effects
NH125	eEF2K	
BIBU 1361 dihydrochloride	EGFR (ERBB1, HER1)	Only 6 out of 34 EGFR inhibitors showed angiogeneic inhibition. Inhibition observed with AEE 788 treatment is likely due to inhibition of Flk1 not EGFR. Similarly inhibition observed with Vandetanib treatment is likely due to targeting KDR/KIT/FLT1/ FLT4 and not EGFR.
WZ3146, WZ-3146	EGFR (ERBB1, HER1)	
BMS-599626, AC-480	EGFR (ERBB1, HER1), ERBB2 (TKR1, HER2, NEU), ERBB3 (HER3), ERBB4 (HER4)	
Dacomitinib hydrate, PF-00299804, PF-00299804-03, PF-299, PF-299804	EGFR (ERBB1, HER1), ERBB2 (TKR1, HER2, NEU), ERBB4 (HER4)	
AEE 788, AEE-788, NVP-AEE-788	EGFR (ERBB1, HER1), ERBB2 (TKR1, HER2, NEU), KDR (VEGFR2, VEGFR, FLK1)	
Vandetanib, Zactima, ZD6474, AZD-6474	EGFR (ERBB1, HER1), KDR (VEGFR2, VEGFR, FLK1), RET, ABL1 (ABL), KIT (c-KIT), FLT1 (VEGFR1), FLT4 (VEGFR3), TRKA (TRK)	
6-bromoindirubin-3'-oxime, BIO	GSK3	Only 3 out of 10 GSK3 inhibitors showed angiogenic inhibition. The more selective GSK inhibitors did not show this effect.
TWS-119	GSK3	
A 1070722, A-1070722	GSK3	
BIRB 796, Doramapimod	p38 MAPK	Only 2 out of 21 p38 inhibitors showed angiogenic inhibition. The more specific p38 inhibitors did not show this effect
LY2228820, LY-2228820	p38a, p38b	
RHO-15	ROCK1, ROCK2 (ROCKa)	Only 2 out of 10 ROCK inhibitors showed angiogenic inhibition. The more specific ROCK inhibitors did not show this effect
Rho Kinase Inhibitor V	ROCK1, ROCK2 (ROCKa)	
ML 9 hydrochloride	smMLCK (MLCK, myosin light chain kinase, MYLK)	This inhibitor is non-specific and only weakly active against MLCK
AT7867, AT-7867	AKT1 (PKBa), AKT2 (PKBb), AKT3 (PKBg), p70S6K (S6K, S6K1)	These few inhibitors resulted in angiogenic increases, however they are known to be non-specific, and the majority of the inhibitors targeting these kinases in the library did not cause this effect.
Enzastaurin, LY-317615	AKT1 (PKBa), PKCb, p70S6K (S6K, S6K1), GSK3B	
SB 216763	GSK3	
SU-11274, PKI-SU11274	MET (c-MET, HGFR)	
Bisindolylmaleimide I hydrochloride, GF 109203X HCl, Go 6850 HCl	PKC	
Bisindoylmaleimide X, HCl salt	PKC	

Compounds, their respective targets and reason considered off-target have been colour-coded

Table S2 (related to Figure 3, Figure 6, and Figure S4). Selectivity of BI-D1870 and AZ3146 for inhibition of EB angiogenic sprouting over having any effect on Lewis lung cells.

In Vivo Drug	*In Vivo* Plasma Concentrations		*In Vitro* Drug	*In Vitro* IC50s		
	60 min	120 min		IC_{50} EBs	IC_{50} (LL/2)	Fold Selectivity (IC_{50} LL2/IC_{50} of EBs)
BI-D1870 (50 mg/kg) i.p	2.81 μM	0.841 μM	BI-D1870	0.16 μM	5.32 μM	33.25
AZ3146 (50 mg/kg) i.p	3.12 μM	1.434 μM	AZ3146	0.72 μM	8.32 μM	11.55

50 mg/kg i.p of BI-D1870 and AZ3146 yielded plasma concentration levels well above the IC_{50} required to inhibit EB angiogenesis and well below the IC_{50} of having any effect on LL/2 cells.

Supplemental Experimental Procedures

Reagents were purchased from Invitrogen unless otherwise specified.

Conditions tested for optimization of vascular differentiation assay
MEF-depleted *Flk1-eGFP* cells were aggregated in suspension at the following concentrations (5,000; 10,000; 50,000; or 100,000 cells/ml). The resulting EBs were embedded in matrix at 3, 4, 5, 6 or 11 days post-aggregation. The matrices tested were growth factor reduced Matrigel (at a working dilution 1:3) or rat tail collagen type I gels (at working concentrations of 0.4 mg/ml, 1 mg/ml, 1.5 mg/ml or 2 mg/ml) both matrices were purchased from BD biosciences. VEGF concentrations tested were 30 ng/ml and 50 ng/ml.

Neuronal Profiling algorithm
The Neuronal Profiling BioApplication V4 from Thermo Fisher Scientific Inc was used. Detailed information regarding the software/algorithm can be obtained using the link below:
http://www.med.cam.ac.uk/wp-content/uploads/2016/02/NeuronalProfiling_V4_LC06190800.pdf
The software was optimized by adjusting thresholds to identify the core of the embryoid body (EB) as the neuron cell nucleus, the parameter of the EB as the neuron cell body and the neurite detection was optimized to detect fluorescent sprouts as the neurites. See Figure S1C for images from the analysis system.

Immunohistochemistry of EBs in collagen gels
Following fixation, EBs in collagen gels were blocked and permeabilized in 5% FBS/0.3% Triton-X100 in 1xPBS, followed by incubation with PECAM-1 (MEC 13.3) (BD Biosciences), anti-mouse DLL4 (R&D systems) or alpha-smooth muscle actin (α-SMA) (1A4) (Sigma-Aldrich). Bound antibodies were detected by Alexa-Fluor 546 secondary antibodies and nuclei were stained with Hoechst 33342. Subsequently, EBs were transferred to glass slides and mounted in Dako fluorescence mounting medium (Agilent) and imaged using a DMI6000B microscope equipped with DFC345-FX camera and LAS-AF software (Leica).

Dose curve validation of hits
EBs were treated with DMSO, VEGF (50 ng/ml) in the presence of DMSO, or VEGF (50 ng/ml) in the presence of varying concentrations of compounds (0.026 μM-2.1 μM), dispensed using the HP D300 Digital Dispenser (Tecan). On day 7 post-embedding, EBs were fixed and the number of FLK-1^{+ve} sprouts and total fluorescent intensity were measured using the Cellomics VTI platform (Thermo Fischer) with an optimized neuronal profiling algorithm as described above.

Cell, tissue lysates and Western blots
Cell and tissue lysates were prepared with RIPA buffer containing protease/phosphatase inhibitors (Cell Signaling Technology). Lysates were resolved by SDS-PAGE, transferred to PVDF membranes, and probed with primary antibodies. The antibodies were: β-ACTIN (AC-15) (Sigma-Aldrich), RSK (32D7), p-RSK (Thr573), LKB1 (D60C5), p-LKB1 Ser428 (C67A3), RPS6 (54D2), p-RPS6 Ser235/236 (D57.2.2E), SMAD2 (D43B4) XP®, and p-SMAD2 (Ser465/467), all from Cell Signaling Technology. Densitometry was performed using Image Studio Lite (Li-COR Biosciences).

HUVEC tube formation assay and disruption of preformed tubes
As per Invitrogen's instructions, HUVECs (75,600 cells/well) were seeded on Geltrex™-coated plates. HUVECS were then either immediately treated with complete media containing DMSO, VEGF (30 ng/ml)+DMSO, BI-D1870 (2 μM), BIX-RSK2 (2 μM), AZ3146 (2 μM) or SU5416 (4 μM) to determine whether these drugs can disrupt tube formation, or treated with these drugs 13 hours post-seeding to determine whether these drugs can disrupt the networks after they had formed. At the end of the experiment, HUVECS were stained with 2 μg/ml Calcein-AM. Networks were imaged at 13, 21, and 23 hours post-seeding using a DMI6000B microscope equipped with DFC345-FX camera and LAS-AF software (Leica).

Cytotoxicity assay
LL/2 cells were seeded ($2x10^3$ cells/well) into 96-well plates and cultured in growth media. At 24 hours post-seeding, DMSO or varying concentrations of BI-D1870 or AZ3146 (0-10 μM) were dispensed using the HP D300 Digital Dispenser (Tecan) and cells were incubated for an additional 72 hours. The cytotoxic

effects of these compounds were evaluated using the ATPlite 1step detection assay kit (PerkinElmer) as per manufacturer's instructions, and the luminescence signal was read on an Envision plate reader (PerkinElmer).

***In vivo* exposure analysis of BI-D1870 and AZ3146**

BI-D1870 and AZ3146 were administered to three mice at 50 mg/kg via intraperitoneal (i.p) injection for 5 days. On day 5, blood samples were collected 60 min and 120 min after the last injection. Plasma was separated from blood through centrifugation and plasma concentration of compounds was determined by liquid chromatography–mass spectrometry. Mouse health and body weight were monitored daily (See Figure S4B).
