# Supplementary material for: Identification of RSK and TTK as Modulators of Blood Vessel Morphogenesis Using an Embryonic Stem Cell-Based Vascular Differentiation Assay
Source: Stem Cell Reports. 2016 Sep 8;7(4):787–801. doi: 10.1016/j.stemcr.2016.08.004 (PMC5063585; doi:10.1016/j.stemcr.2016.08.004)
Supplement: Document S2. Article plus Supplemental Information [file mmc3.pdf]

Stem Cell Reports
Resource
OPEN ACCESS

Identification of RSK and TTK as Modulators of Blood Vessel Morphogenesis Using an Embryonic Stem Cell-Based Vascular Differentiation Assay

Lamis Hammoud,[1] Jessica R. Adams,[1] Amanda J. Loch,[1] Richard C. Marcellus,[3] David E. Uehling,[3] Ahmed Aman,[3] Christopher Fladd,[4] Trevor D. McKee,[5] Christine E.B. Jo,[1] Rima Al-Awar,[3] Sean E. Egan,[1,2] and Janet Rossant[1,2,*]
[1]Program in Developmental and Stem Cell Biology, Peter Gilgan Centre for Research and Learning, The Hospital for Sick Children, 686 Bay Street, Toronto, ON M5G 0A4, Canada
[2]Department of Molecular Genetics, University of Toronto, Toronto, ON M5S 1A8, Canada
[3]Drug Discovery Department, Ontario Institute for Cancer Research, Toronto, ON M5G 0A3, Canada
[4]SPARC BioCentre, The Hospital for Sick Children, Toronto, ON M5G 0A4, Canada
[5]Radiation Medicine Program, STTARR Innovation Centre, Princess Margaret Cancer Centre, Toronto, ON M5G 1L7, Canada
*Correspondence: janet.rossant@sickkids.ca
http://dx.doi.org/10.1016/j.stemcr.2016.08.004

SUMMARY

Blood vessels are formed through vasculogenesis, followed by remodeling of the endothelial network through angiogenesis. Many events that occur during embryonic vascular development are recapitulated during adult neoangiogenesis, which is critical to tumor growth and metastasis. Current antiangiogenic tumor therapies, based largely on targeting the vascular endothelial growth factor pathway, show limited clinical benefits, thus necessitating the discovery of alternative targets. Here we report the development of a robust embryonic stem cell-based vascular differentiation assay amenable to small-molecule screens to identify novel modulators of angiogenesis. In this context, RSK and TTK were identified as angiogenic modulators. Inhibition of these pathways inhibited angiogenesis in embryoid bodies and human umbilical vein endothelial cells. Furthermore, inhibition of RSK and TTK reduced tumor growth, vascular density, and improved survival in an in vivo Lewis lung carcinoma mouse model. Our study suggests that RSK and TTK are potential targets for anti-angiogenic therapy, and provides an assay system for further pathway screens.

INTRODUCTION

Pluripotent embryonic stem cells (ESCs) provide essential tools for understanding mammalian developmental processes, as they can differentiate in vitro into many tissues in a normal developmental manner (Keller, 2005; Solter, 2006). These cells are amenable to high-throughput screens using RNAi or small-molecule libraries to dissect molecular pathways (Ding and Buchholz, 2006; Xu et al., 2008). Early vascular and hematopoietic differentiation of ESCs has been extensively studied (Keller, 2005), making these pathways particularly attractive for large-scale screens.

Blood vessels are first formed through vasculogenesis, whereby angioblasts (endothelial precursors) aggregate in the developing embryo to form a primitive network of endothelial tubes. This network is later remodeled through a complex process termed angiogenesis, which includes sprouting of new blood vessels, to form the mature circulatory network (Rossant and Howard, 2002). Major breakthroughs in our understanding of vascular development and remodeling have arisen from characterization of vascular mutant phenotypes in mice. Vascular endothelial growth factor (VEGF), acting through the FLK-1/VEGF receptor 2 (VEGFR2), is crucial for blood vessel formation and development (Carmeliet et al., 1996; Shalaby et al., 1995). NOTCH/DLL4 signaling plays a critical role in branching/sprouting morphogenesis, whereby loss of NOTCH signaling leads to excess tip cell formation and non-productive vessel development (Hellstrom et al., 2007). Impaired vascular development was also reported for mutations in ANG/TIE, platelet-derived growth factor (PDGF), transforming growth factor β (TGF-β), EFN, HH, and PLXN/SEMA signaling pathways (reviewed by Rossant and Howard, 2002).

Many signaling pathways required during embryonic vascular development are also essential during adult neoangiogenesis (Carmeliet, 2003). Adult neovascularization occurs in many physiological and pathological settings, such as wound healing (Ruiter et al., 1993), recovery from myocardial infarction (Chung et al., 2002), tumor growth, and metastasis (Ruiter et al., 1993). There is increasing interest in using modulators of angiogenesis to treat cancer (Ferrara, 2004). Currently antiangiogenic therapy has two opposing target pathways, the VEGF/FLK-1 and DLL4/NOTCH pathways (Kuhnert et al., 2011). The new generation of antiangiogenic drugs that have arisen from an understanding of vascular developmental biology, such as bevacizumab (anti-VEGF) (Ferrara et al., 2005), have demonstrated some efficacy in cancer patients, but cause serious side effects and frequent relapses (Kerbel, 2008). Similar results have been obtained from inhibition of the NOTCH/DLL4 pathway (Andersson and Lendahl, 2014), thus necessitating the discovery of alternative therapeutic targets.

© 2016 The Author(s).
This is an open access article under the CC BY license (http://creativecommons.org/licenses/by/4.0/).

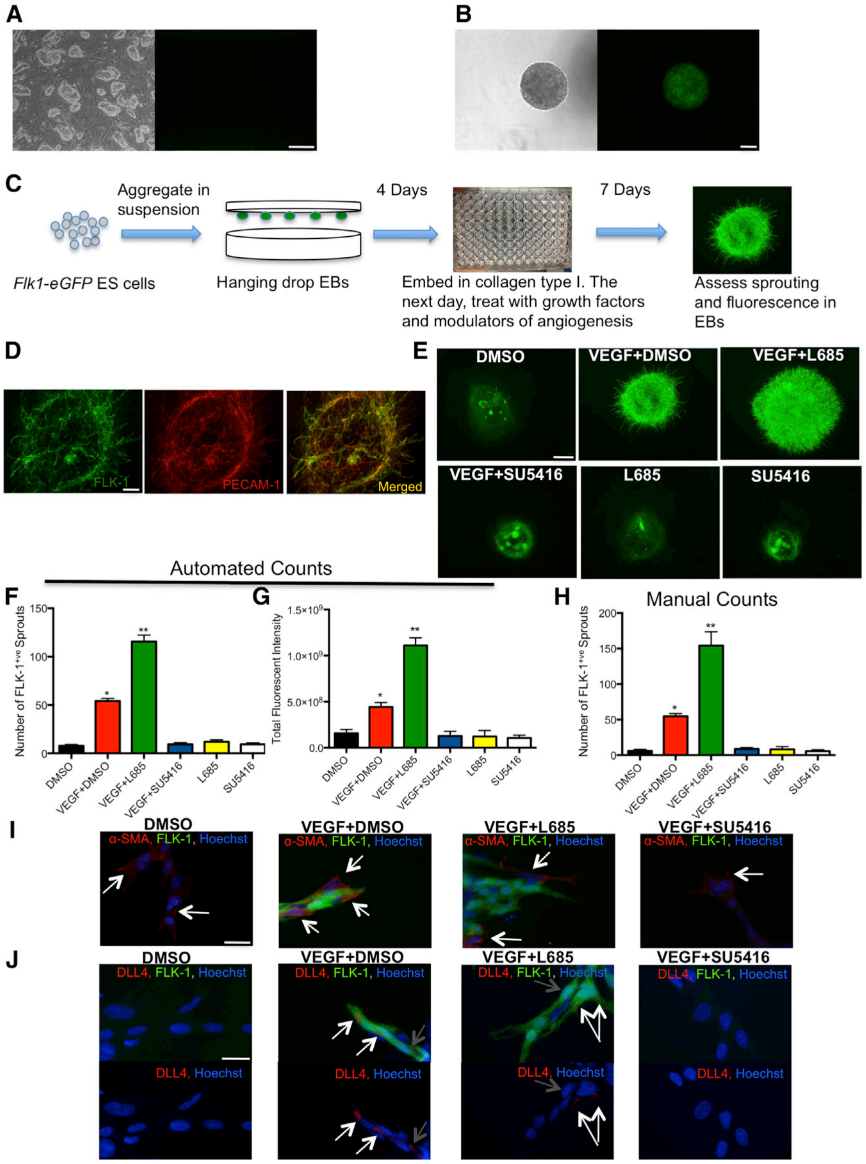

Figure 1. Development and Validation of ESC-Based Vascular Differentiation Assay

(A) Undifferentiated *Flk1-eGFP* ESCs under phase contrast (left panel) and GFP filter (right panel). Scale bar, 100 μm.

(B) *Flk1-eGFP* ESCs were aggregated in suspension in hanging drops for 4 days and observed under phase contrast (left panel) and GFP filter (right panel). Scale bar, 100 μm.

(C) Schematic representation of vascular differentiation assay method.

(D) PECAM-1 staining of *Flk1-eGFP*-derived EBs embedded in collagen type I gel and treated with VEGF (50 ng/mL), bFGF (100 ng/mL), IL-6 (10 ng/mL), and EPO (2 U/mL). Scale bar, 500 μm.

(E) Validation of the assay using two small-molecule inhibitors, a NOTCH inhibitor (L685, 5 μM), and an FLK-1 inhibitor (SU5416, 4 μM). Scale bar, 500 μm.

(F and G) Cellomics was used to quantify FLK-1^{+} sprouts (F) and FLK-1 expression (measured as total fluorescent intensity) (G).

(H) Quantification of FLK-1^{+} sprouts obtained by manual counts. Data are mean ± SEM, n ≥ 3. *$p < 0.0001$, statistically significant compared with DMSO control; **$p < 0.0001$, statistically significant compared with DMSO and VEGF + DMSO controls.

(I and J) Characterization of vascular sprouts of *Flk1-eGFP*-derived EBs treated as indicated. (I) Anti-α-SMA staining (white arrows point to mural cells). Scale bar, 25 μm. (J) Anti-Dll4 staining (white arrows point to tip cells, gray arrows point to stalk cells). Scale bar, 25 μm.

See also Figure S1.

To this end we have developed a robust, highly reproducible, mouse ESC-based vascular differentiation assay that is sensitive to both inhibition and promotion of vascular sprouting as well as to changes in vessel morphology. Using our embryoid body (EB)-based assay, we undertook a kinase inhibitor screen to identify small molecules that could block or enhance blood vessel sprouting morphogenesis. The screen yielded numerous hits, which we validated in vitro and subsequently tested for in vivo antiangiogenic activity in a Lewis lung (LL/2) carcinoma model. We have identified RSK and TTK as potential targets for antiangiogenic tumor therapy, and provide an assay system for further pathway screens.

RESULTS

Development of a Robust, and Reproducible Vascular Differentiation Assay Using ESCs

We have previously reported the generation of ESCs whereby EGFP was inserted into the *Flk-1* locus, and showed that this reporter faithfully recapitulates all areas of FLK-1 expression (Ema et al., 2006). As predicted, no EGFP was observed in the undifferentiated ESCs (Figure 1A), and high levels of EGFP were observed when ESCs were differentiated into EBs (Figure 1B). To optimize the vascular differentiation assay (Figure 1C), we aggregated *Flk1-eGFP* ESCs in suspension as hanging drops to form EBs. Different cell concentrations, types of matrices, and different days for

embedding of EBs were tested (see Supplemental Experimental Procedures). We determined that EBs generated from 200 cells and embedded in collagen type I gels at day 4 gave the most consistent and reproducible results. There was no significant difference in the number of FLK-1 positive (FLK-1$^+$) sprouts between EBs treated with VEGF only and EBs treated with VEGF in the presence of one or more of the previously established angiogenic growth factors (basic fibroblast growth factor [bFGF], interleukin-6 [IL-6], and erythropoietin [EPO]) (Feraud et al., 2001) (Figure S1A), suggesting that VEGF alone accounts for the majority of the angiogenic response and is the only factor required in our assay. PECAM-1 staining showed nearly complete overlap with the *Flk1-eGFP* reporter both in the primary vascular plexus formed in the EBs and the angiogenic sprouts extending from the EBs (Figure 1D). We also determined that the optimal point for quantification of FLK-1$^+$ sprouts was day 7 after embedding, as abundant sprouting was observed and both increases and decreases in angiogenesis would be readily measurable (Figures S1B, S1C, and 1E–1H). Using the Cellomics ArrayScan platform, we optimized the neuronal profiling algorithm to objectively quantify the number of FLK-1$^+$ sprouts and the total expression of FLK-1 (measured as total fluorescent intensity). This algorithm demonstrated that our assay can detect both increases and decreases in angiogenesis in response to signaling pathway inhibitors (Figures 1F, 1G, and S1C). Visual inspection confirmed automated counts (Figure 1H). The reproducibility of VEGF + DMSO control, as measured by FLK-1$^+$ sprout quantification, is shown in Figures S1D and S1E. Treatment with γ-secretase inhibitor (L685,458; referred to hereafter as L685) in the presence of VEGF significantly increased angiogenic sprouting by ≥113.4% (Figures 1E, 1F, and 1H) and total FLK-1 expression by 150.6% compared with VEGF + DMSO control (Figures 1E and 1G), as expected for a NOTCH pathway inhibitor. Treatment with an FLK-1 inhibitor (SU5416) significantly decreased VEGF-induced angiogenic sprouting by ≥82.9% (Figures 1E, 1F, and 1H) and FLK-1 expression by 71% (Figures 1E and 1G). Treatment with L685 or SU5416 in the absence of VEGF resulted in a similar number of FLK-1$^+$ sprouts and expression as DMSO controls (p not significant), suggesting that very little angiogenesis occurs in the absence of VEGF (Figures 1E–1H). Given these findings, all inhibitors were added in the presence of VEGF. To characterize the sprouts, we stained them with α-smooth muscle actin (α-SMA) or DLL4, markers for mural cells and tip cells, respectively. VEGF + DMSO-treated EBs showed mural cells surrounding the sprouts (Figure 1I) and high levels of DLL4 in tip cells, whereas DLL4 staining was reduced or absent in the stalk cells (Figure 1J). VEGF + L685-treated EBs showed reduced mural cells (Figure 1I) and decreased DLL4 staining in tip cells (Figure 1J), consistent with previous studies on the effect of NOTCH inhibition on mural cell differentiation and DLL4 expression (Arima et al., 2011). Some mural cells were observed in DMSO- and VEGF + SU5416-treated EBs (Figure 1I), but DLL4 was absent (Figure 1J).

We also demonstrate that our assay, in addition to being sensitive to increases and decreases in vessel sprouting, can also detect morphological changes in vessel shape (Figures S1F and S1G).

Small-Molecule Kinome Screen for Modulators of Angiogenesis

To identify novel modulators of angiogenesis, we used our assay to screen a kinase small-molecule inhibitor library (Figures 2A and 2B) consisting of 480 compounds. Hits were registered as quantitative deviations from VEGF control cultures (Figure S2), since VEGF was also added to every well containing the inhibitors. Automated quantification successfully distinguished neutral events and increases, although distinction between inhibitory and toxic hits had to be confirmed by visual inspection. Representative images of the hits and reproducibility of the phenotypes between replicates are shown in Figures 2C–2N. Graphical representation of the hits is shown in Figure 2O. A hit was considered real/specific if the majority of compounds that were known to inhibit that particular target in the library showed activity. Table 1 lists hits that met these criteria. A few compounds resulted in a NOTCH loss-of-function-like phenotype. For example, a PKC inhibitor resulted in excessive sprouting; however, since the majority of the library compounds targeting PKC showed no effect on angiogenesis, the phenotype was deemed to be an off-target effect and not pursued further (Table S1). Our library screen detected 40 of the 44 compounds that target FLK-1, thus further validating our vascular assay and screening methodology. The majority of hits were validated with dose curves (Figure S3). Our screen identified many kinases with well-established roles in angiogenesis such as FLK-1(Shalaby et al., 1995), TIE2 (Partanen et al., 1996), PDGFRβ (PDGF receptor β) (Rossant and Howard, 2002; Zhang et al., 2009), ALK (anaplastic lymphoma kinase) (Di Paolo et al., 2011), ALK5 (TGFBR1) (Rossant and Howard, 2002), BMK1 (ERK5) (Hayashi et al., 2005; Pi et al., 2005), FGFR (FGF receptor) (Bono et al., 2013), IGFR (insulin-like growth factor receptor) (Bid et al., 2012), MEK1/2 (Giroux et al., 1999), and ERK1/2 (Srinivasan et al., 2009), among others (Table 1). Importantly, we also identified RSK and TTK in the screen; kinases that have not been previously shown to regulate angiogenesis. Our inhibitory hits all fall into one of six signaling pathways (Figure 2P). Criteria for pursuing hits are summarized in Figure S2.

Compound and Target Hit Validation

To validate RSK as a hit, we performed a dose-response curve using the inhibitors identified by our screen,

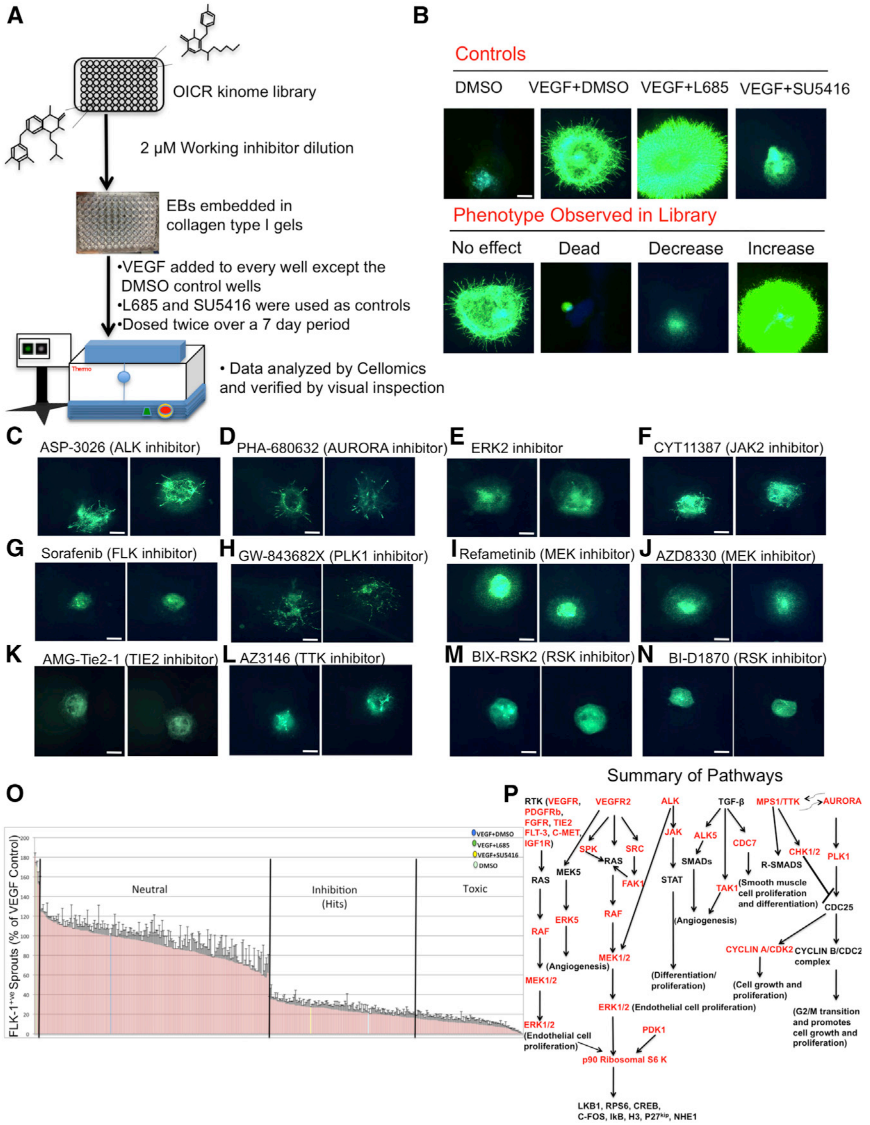

Figure 2. Kinome Small-Molecule Library Screen

(A) Schematic of the methodology for screening the small-molecule library. The screen was done blinded.

(B) Controls and phenotypes observed in library. Scale bar, 300 μm.

(C–N) Representative examples of hits and reproducibility of the phenotype between replicates. Scale bar, 300 μm.

(O) Graphical summary of hits. n = 4 technical replicates; data are normalized to VEGF + DMSO controls and expressed as mean ± SEM.

(P) All hits in the screen fall into one of the six outlined pathways. Red indicates targets identified in the screen.

See also Figures S2 and S3; Tables S1 and S3.

BIX-RSK2 (referred to as compound 15 in the study by Fryer et al., 2012) (Figure 3A) and BI-D1870 (Figure 3B). These inhibitors have previously been shown to be selective (Fryer et al., 2012; Sapkota et al., 2007). It is important to note that BI-D1870 at high concentrations (10 μM or above) can have off-target effects, although this inhibitor was shown to be selective when used at 2.5 μM and lower (Roffe et al., 2015; Sapkota et al., 2007). RSKs (RSK1–4) are a family of serine/threonine kinases that share 75%–80% amino acid identity and are activated by the MAPK pathway through a series of phosphorylation events (Anjum and Blenis, 2008). Interestingly, RSK protein levels are elevated in several tumor types (Clark et al., 2005; Smith et al., 2005). Downstream substrates of RSKs include CREB, c-FOS, IκB, LKB1, and RPS6 (Anjum and Blenis, 2008; Romeo et al., 2012). Notably, LKB1 is required for vascular development (Londesborough et al., 2008). We confirmed, through western blot analysis, that BI-D1870 and BIX-RSK2 were targeting the RSK/LKB1 pathway, which is downstream of MEK/ERK. Treatment with VEGF in the presence of 2 μM of either the MEK1/2 inhibitor (GSK-1120212), ERK1/2 inhibitor (ERK2), or RSK inhibitors (BI-D1870, BIX-RSK2) resulted in a significant decrease in p-RSK levels (≥46.6% decrease, $p < 0.01$) and p-LKB1 (≥46.4% decrease, $p < 0.0001$) compared with the VEGF + DMSO control (Figure 3C). A schematic of the pathway is depicted in Figure 2P. VEGF + DMSO-treated EBs also showed slightly elevated total RSK and total LKB1 levels (Figure 3C), perhaps due to stabilization of these proteins in response to phosphorylation/activation.

Table 1. Hits Identified in Vascular Screen

Categories	Drug Target	Number of Inhibitors that Hit Target/Total Number of Inhibitors of that Target in Library	Number of Dose Curves (Validation)	References (Angiogenesis)
Cell surface receptors	ALK	3/4		Di Paolo et al., 2011; Safina et al., 2007
	ALK5	8/10	1	Rossant and Howard, 2002
	c-MET	13/20	1	Lu and Bergers, 2013
	FGFR	11/13	2	Bono et al., 2013
	FLK1	40/44	2	Ferrara et al., 2005; Shalaby et al., 1995
	FLT1/FLT4/KIT	6/6	1	Sleijfer et al., 2009
	FLT3/FLK2, SYK	22/24, 4/4	2	Kazerounian et al., 2011
	IGFR	4/7	3	Bid et al., 2012
	PDGFRβ	26/36		Zhang et al., 2009
	TIE2	5/5	1	Partanen et al., 1996
MAPK pathway	ERK2	4/6	1	Srinivasan et al., 2009
	ERK5	1/2	1	Hayashi et al., 2005; Pi et al., 2005
	FAK	3/4	1	Tavora et al., 2010
	MEK1/2	12/15	2	Giroux et al., 1999
	PDK1	3/5	1	Tawaramoto et al., 2012
	RAF	11/17	2	Wimmer et al., 2012
	SRC/FYN/ABL/LCK	5/7	4	Schenone et al., 2007
	TAK1 (MAP3K7)	3/3		Jadrich et al., 2006
	p90 ribosomal S6K (RSK)	2/2	2	
JAK/STAT pathway	JAK	13/20	1	Xin et al., 2011
Cell-cycle regulators	AURORA	19/21		Romain et al., 2014
	CDC7	1/1		Shi et al., 2012
	CDK2/CYCLIN A	13/20		Chen et al., 2000
	MPS1 (TTK)	1/1	1	
	CHK1/2	8/11	2	
	PLK1	6/8	2	Gomes et al., 2013
	SPK1	2/3	1	Duan et al., 2007

See also Figure S3.

To validate TTK (MPS1) as a bona fide hit, we performed dose-response analysis using AZ3146 (Figure 3D), a TTK inhibitor that was identified in our screen and has been shown to be selective (Hewitt et al., 2010). TTK, a dual-specificity kinase that phosphorylates serine, threonine, and tyrosine residues, is an essential component of the spindle assembly checkpoint and is required for chromosomal alignment during mitosis (Liu and Winey, 2012). TTK expression is elevated in multiple cancers (breast, lung, and gastric cancer) (Liu and Winey, 2012). Downstream targets of TTK include CHK2 (Liu and Winey, 2012) and SMAD2/3 (Zhu et al., 2007). Notably, SMAD2 has been implicated in angiogenesis (Assis et al., 2015; Pen et al., 2008). Our data showed that treatment with VEGF + AZ3146 resulted in a 73.5%

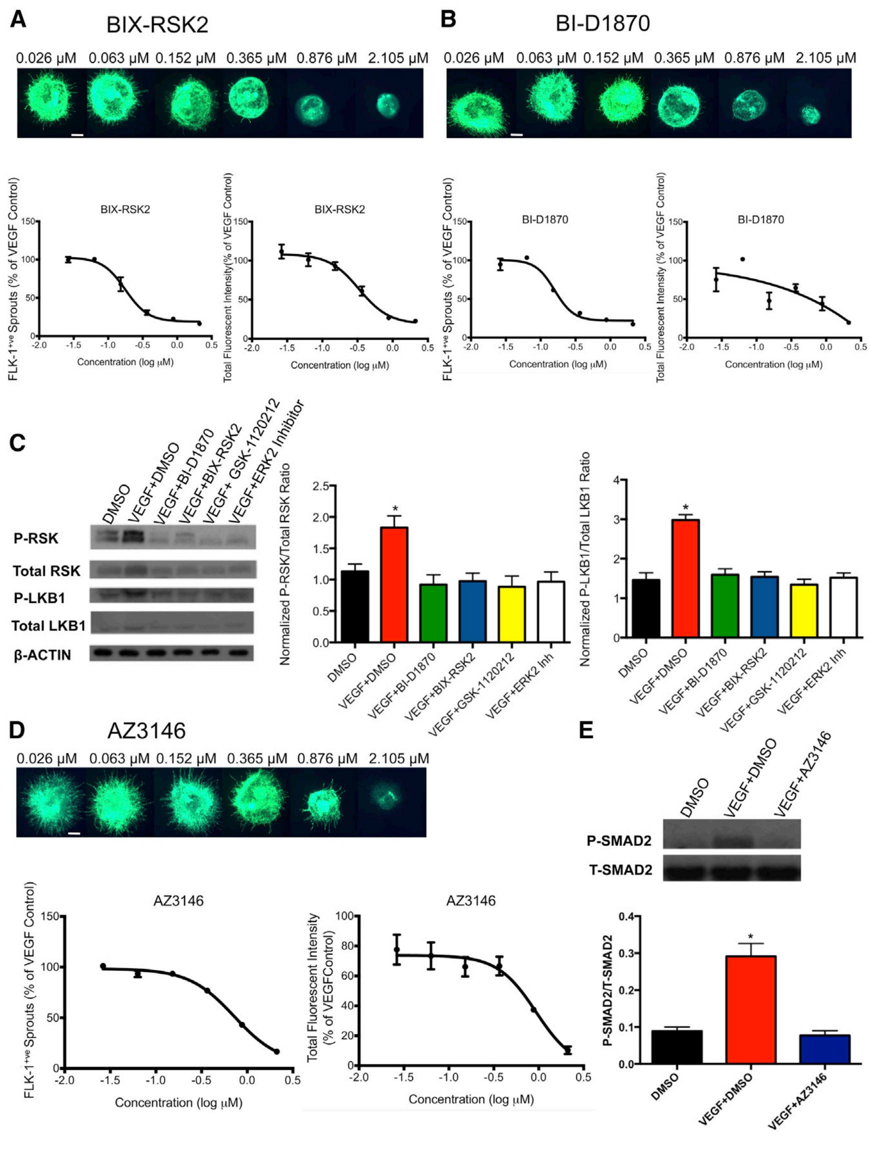

Figure 3. Validation of RSK and TTK Inhibitors in EBs

EBs embedded in collagen type I were treated with DMSO, VEGF (50 ng/mL) + DMSO, or VEGF (50 ng/mL) in the presence of inhibitors and dosed twice over a 7-day period. On day 7, EBs were either fixed, imaged and quantified for dose-curve analysis, or lysed in RIPA buffer for western blot analysis. Asterisk denotes statistical significance compared with DMSO control.

(A) Dose-curve analysis of BIX-RSK2.

(B) Dose-curve analysis of BI-D1870.

(C) Western blot analysis showing the effect of 2 μM of various inhibitors on phosphorylated and total levels of LKB1 and RSK. Data were normalized to β-ACTIN. Data are mean ± SEM, n = 5. $*p < 0.01$ for normalized P-RSK/total RSK, and $*p < 0.0001$ for normalized P-LKB1/total LKB1.

(D) Dose-curve analysis of AZ3146.

(E) Western blot analysis showing the effect of 2 μM AZ3146 on phosphorylated and total levels of SMAD2. Data are mean ± SEM, n = 6. $*p < 0.0001$.

For dose-curve analysis (A, B, and D), values were normalized to VEGF + DMSO controls. Drug doses were log transformed. Data are mean ± SEM, n = 4 technical replicates. Scale bars, 300 μm. See also Table S2.

decrease ($p < 0.0001$) in SMAD2 phosphorylation compared with VEGF + DMSO control (Figure 3E).

The Effect of RSK and TTK Inhibitors on Disruption of Angiogenic Sprouts

To determine whether BI-D1870, BIX-RSK2, and AZ3146 can disrupt preformed angiogenic sprouts, we treated EBs with VEGF for 6 or 7 days before addition of inhibitors, as abundant angiogenesis was observed at these time points (Figures S1B and 1E–1H). BI-D1870, BIX-RSK2, AZ3146, or the known FLK-1 inhibitor SU5416 (Fong et al., 1999) were then added in the presence of VEGF on days 6 or 7. Cultures were maintained for three additional days and fixed. BI-D1870 (Figure 4B), BIX-RSK2 (Figure 4C), and SU5416 (Figure 4E) resulted in significantly reduced FLK-1^{+} sprouts (≥57.2%, $p < 0.0001$) compared with the VEGF + DMSO controls (Figure 4A). AZ3146 resulted in a significant decrease (21.3%) in FLK-1^{+} sprouts when added on day 6 but not on day 7 (Figure 4D). All four drugs significantly decreased angiogenesis by ≥78.1% ($p < 0.0001$) when added in the presence of VEGF on day 1 post embedding (Figures 4B–4E).

The Effect of RSK and TTK Inhibitors on HUVEC Tube Formation and Disruption of Preformed Tubes

We sought to validate our top hits in a secondary human-relevant cell-based assay, using human umbilical vein endothelial cells (HUVECs). This also allowed us to determine whether inhibition of RSK and TTK, which are expressed in multiple cell types, had a direct effect on endothelial cells.

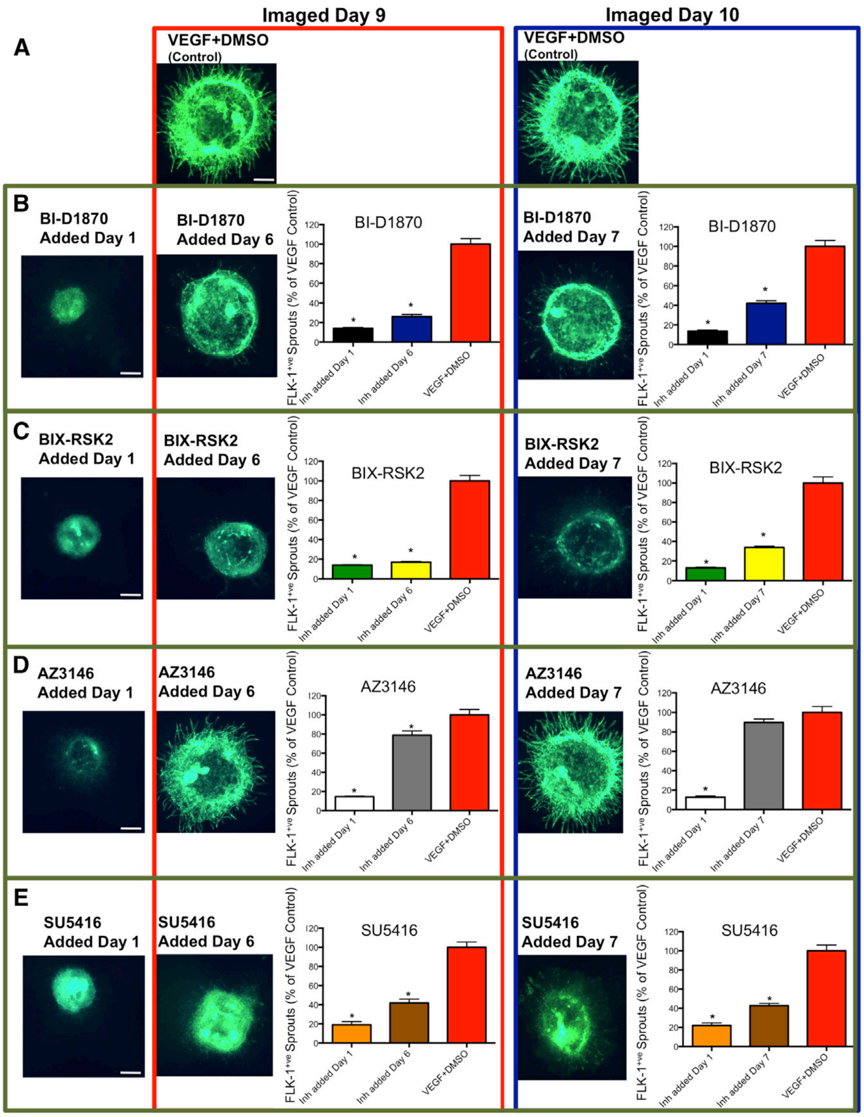

Figure 4. Effect of RSK and TTK Inhibitors on Preformed EB Angiogenic Sprouts

RSK and TTK inhibitors (2 μM) or SU5416 (4 μM) were added either at day 1 post embedding, in the presence of VEGF (50 ng/mL), or added on days 6 or 7 post VEGF treatment, also in the presence of 50 ng/mL VEGF, and imaged on days 9 or 10 post embedding, respectively, and the number of FLK-1^{+} sprouts quantified.

(A) VEGF + DMSO controls.

(B) Effect of BI-D1870.

(C) Effect of BIX-RSK2.

(D) Effect of AZ3146.

(E) Effect of SU5416.

Inh, Inhibitors. Data were normalized to VEGF + DMSO controls. Data are mean ± SEM, n $\geq$ 3. *p < 0.0001 compared with VEGF + DMSO controls. Scale bars, 300 μm. See also Figure S1B.

No difference was observed in network morphology between DMSO and VEGF controls, suggesting that complete medium which was not supplemented with VEGF but contained bFGF was sufficient to promote network formation (Figures 5A and 5B). BI-D1870, BIX-RSK2, AZ3146, and SU5416 resulted in disruption of the networks compared with the DMSO or VEGF controls (Figure 5A). Furthermore, when these drugs were added 13 hr post plating, after the networks were already established, they were also able to disrupt the preformed HUVEC tubes (Figure 5B).

The Effect of RSK and TTK Inhibitors on LL/2 Cells In Vitro and Analysis of In Vivo Exposure to These Drugs

The LL/2 model was chosen to examine the effect of our hits on tumor angiogenesis, as it is a widely used model for studying angiogenesis (Eklund et al., 2013) and is effective in predicting clinical benefit (Chow and Eckhardt, 2007). As BIX-RSK2 is not commercially available, we focused on BI-D1870 and AZ3146 for the remainder of our study. BI-D1870 and AZ3146 had no cytotoxic effects on the LL/2 cells in vitro except at very high doses (Figure S4A). The IC_{50} values showed that BI-D1870 displayed 33.25-fold selectivity, and AZ3146 showed 11.55-fold selectivity for inhibition of EB angiogenic sprouting over LL/2 cell growth inhibition (Table S2). In vivo exposure analysis showed that a dose of 50 mg/kg for BI-D1870 and AZ3146 via intraperitoneal injection resulted in plasma concentrations well above the IC_{50} for inhibiting angiogenesis of the EBs and well below the IC_{50} for having any effect on the LL/2 cells (Table S2). Furthermore, these doses were well tolerated by the mice, with

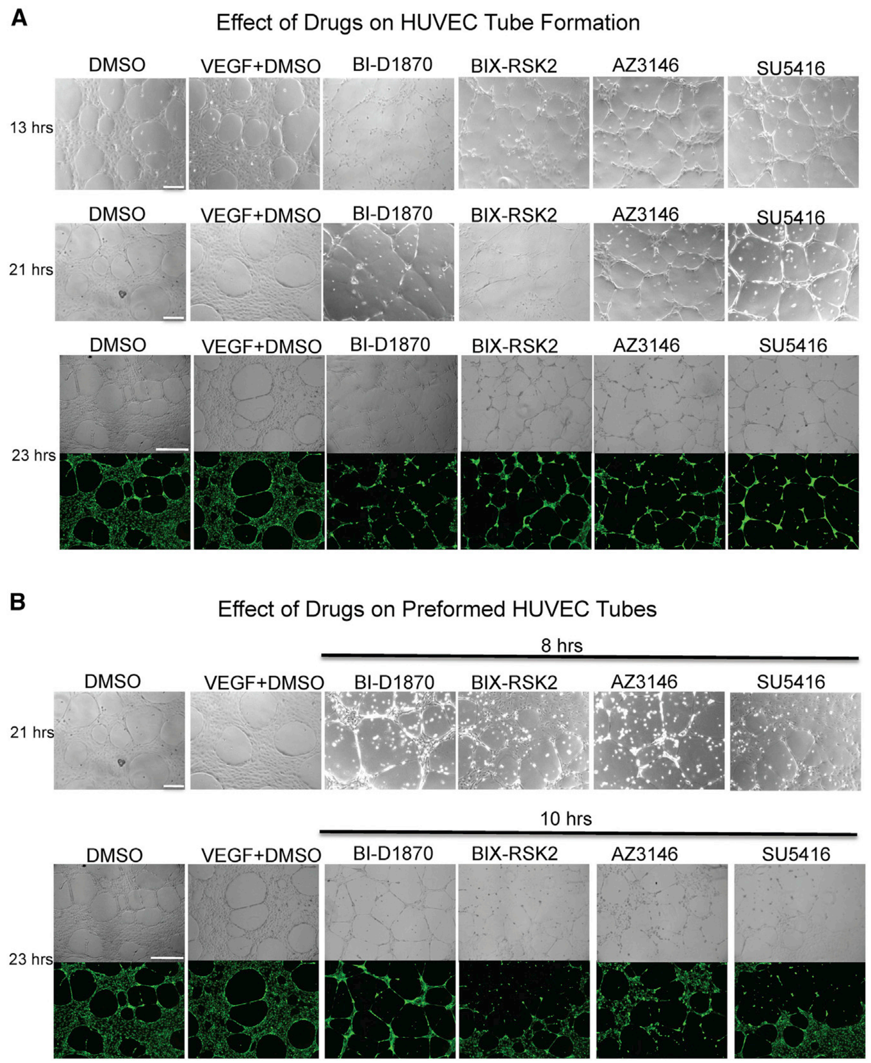

Figure 5. Effect of RSK and TTK Inhibitors on HUVEC Tube Formation and on Preformed HUVEC Tube Networks

(A and B) represent two parts of the same experiment and share DMSO and VEGF + DMSO controls. HUVECs were plated on Geltrex, and either (A) immediately treated with complete HUVEC medium containing DMSO, or VEGF (30 ng/mL) + DMSO, or 2 μM BI-D1870, BIX-RSK2, or AZ3146, or 4 μM SU5416, and imaged at 13 hr (scale bar, 200 μm), 21 hr (scale bar, 200 μm) and 23 hr (scale bar, 500 μm) post seeding; or (B) treated 13 hr post plating with 2 μM BI-D1870, BIX-RSK2, or AZ3146, or 4 μM SU5416, and imaged at 21 hr (8 hr drug treatment; scale bar, 200 μm) and 23 hr (10 hr drug treatment; scale bar, 500 μm) post seeding. Calcein-AM was added at the end of the experiment and is shown as green fluorescence in the bottom panels of (A) and (B).

no significant changes in weight or behavior observed (Figure S4B).

The Effect of the RSK and TTK Inhibitors on Survival, Tumor Growth, and Angiogenesis In Vivo

To test for the efficacy of these compounds on tumor growth and angiogenesis in vivo, we treated LL/2 tumor-bearing mice with vehicle, BI-D1870, AZ3146, or SU5416 via intraperitoneal injections daily for 14 days (Figure 6A). BI-D1870 and AZ3146 significantly improved survival (Figure 6B) and significantly decreased tumor volume from days 8 and 7 onward, respectively, with a ~37% and ~32% decrease in tumor volume by day 14 post treatment (Figure 6C, $p < 0.05$). PECAM-1 staining of tumors excised at day 14 post treatment showed that BI-D1870 and AZ3146 significantly decreased vessel density (~38% and ~35%, respectively, $p < 0.01$) (Figure 6D). Conversely, the well-established antiangiogenic FLK-1 inhibitor SU5416 had no effect on survival (Figure 6B) or tumor volume (Figure 6C), and did not significantly decrease vessel density (Figure 6D). The dose of SU5416 we used has been reported to be the maximum effective tolerated dose for that compound (Fong et al., 1999). Quantification of vessel density in normal host tissue showed no significant difference between vehicle-treated and drug-treated groups (Figure S4C).

To determine the effect of these compounds on signaling, we performed western blot analysis on tumors. BI-D1870 and SU5416 had no effect on RSK (Figure 6E) or LKB1 phosphorylation (Figure 6F) but resulted in a significant decrease (≥64.2%, $p < 0.001$) in phosphorylation of RPS6 (Figure 6G), a target of RSK (Anjum and Blenis, 2008; Romeo et al., 2012) and the VEGF pathway (Jeong et al., 2014). AZ3146 significantly decreased (35.8%, $p < 0.05$) phosphorylation of SMAD2, a target of TTK (Zhu et al., 2007) (Figure 6H). Both RPS6 (Hayashi et al., 2005)

and SMAD2 (Assis et al., 2015; Pen et al., 2008) have previously been associated with angiogenesis.

DISCUSSION

We have developed an unbiased, robust, and reproducible three-dimensional EB-based vascular differentiation assay that is amenable to screening for modulators of angiogenesis. The EB-based vascular differentiation assay in collagen matrix offers advantages over the widely used HUVEC/Matrigel assay as well other in vitro angiogenic models in that it uniquely allows the study of both vasculogenesis and angiogenesis (Feraud et al., 2001). The EB assay, unlike the HUVEC assay, models the complex in vivo interactions between endothelial cells and their support cells, which is essential for recapitulating normal vessel formation (Feraud et al., 2001). The assay is sensitive to both increases and decreases in vessel sprouting as well as reading out morphological changes in vessel shape, as exemplified by an additional screen that showed treatment with all-*trans* retinoic acid resulted in the ballooning of vascular sprouts (Figures S1F and S1G).

Previous reports have described ESC-based differentiation in collagen gels to study the developmental events of vasculogenesis and angiogenesis (Feraud et al., 2001; Hermant et al., 2007). However, these were not optimized for the assessment of more fundamental aspects of vessel induction, patterning, and remodeling, nor were these assays standardized into a 96-well plate format suitable for screening. In some previous reports, EB size was not controlled (Feraud et al., 2001; Hermant et al., 2007), which is essential for obtaining the consistency and reproducibility required in a screen. Additionally in these assays, multiple growth factors for vascular induction were used (Feraud et al., 2001; Hermant et al., 2007), or high concentrations of cells were used for EB formation (Jakobsson et al., 2006), which increases variability. Building on the foundation of these studies, we have standardized and simplified the culture system and employed a fluorescent reporter to allow easy monitoring of morphogenesis, thus producing a more robust assay suitable for drug screens in the mouse system. Future studies using human pluripotent cell lines, aided by the advances in genome-editing technologies, will allow the use of more robust reporter lines for endothelial differentiation in the human system.

Our assay was validated using NOTCH and FLK-1 inhibitors, since disruption of these pathways results in visible alterations in angiogenesis (Hellstrom et al., 2007; Shalaby et al., 1995). By screening a small-molecule kinome library we expected a large number of hits, given that the vasculature is very sensitive to signaling pathway disruption. We identified many kinase targets with well-established roles in angiogenesis, including RTKs (VEGFR, PDGFR, FGFR, TIE2, FLT-3, c-MET, and IGF1R) as well as their downstream effectors including RAF, MEK, and ERK, further validating our screen. JAK, ALK, ALK5, and AURORA were also hits and have well-established roles in regulating angiogenesis. It is important to note that despite the fact that our screen is designed to detect both promoters and inhibitors of angiogenesis, all of our validated hits inhibit angiogenesis. It is possible that the NOTCH pathway may be unique in causing excessive sprouting. Interestingly, inhibition of ALK1 has also been shown to lead to excessive angiogenic sprouting, which was attributed to cooperation of ALK1 with the NOTCH pathway (Kerr et al., 2015). An additional screen of a more broad-based library similarly showed that only NOTCH inhibitors resulted in excessive angiogenic sprouting (data not shown). Screening of this second library showed that our assay is sensitive to phenotypic changes that were measurable beyond just increases or decreases in the number of FLK-1^{+} sprouts (i.e., retinoids had no major effect on sprout number but caused morphological changes in vessel shape). This suggests that the complete landscape of target space that can be explored with this assay is still to be determined.

We identified RSK and TTK as angiogenic modulators. We showed that treatment of EBs or HUVECS with BI-D1870 and BIX-RSK2, the selective RSK inhibitors, or with AZ3146, the selective TTK inhibitor, inhibited angiogenic sprouts in EBs and network formation in HUVECs, and disrupted the preformed HUVEC tubes and the preformed EB angiogenic sprouts. It is important to note that these inhibitors disrupted network formation in HUVECs induced by bFGF without VEGF supplementation, suggesting that they are downstream of multiple proangiogenic pathways. Western blot analysis of EBs showed that TTK and RSK inhibitors resulted in a significant decrease in phosphorylation of the downstream targets, SMAD2 and LKB1, respectively, in association with the observed decrease in angiogenesis. A previous report has suggested the involvement of RSK in angiogenesis, although no direct evidence was provided (Hayashi et al., 2005). Our study provides direct proof that RSK and TTK regulate angiogenesis.

Future studies using genetic approaches involving the generation of ESC lines with inducible gene knockout of RSK and TTK need to be performed to further validate our hits.

To determine whether the inhibition of RSK and TTK inhibits neovascularization in vivo, we gave mice LL/2 tumor grafts. Both BI-D1870 and AZ3146, used at doses determined to be non-toxic to animals, significantly improved survival, inhibited tumor growth, and decreased vascular

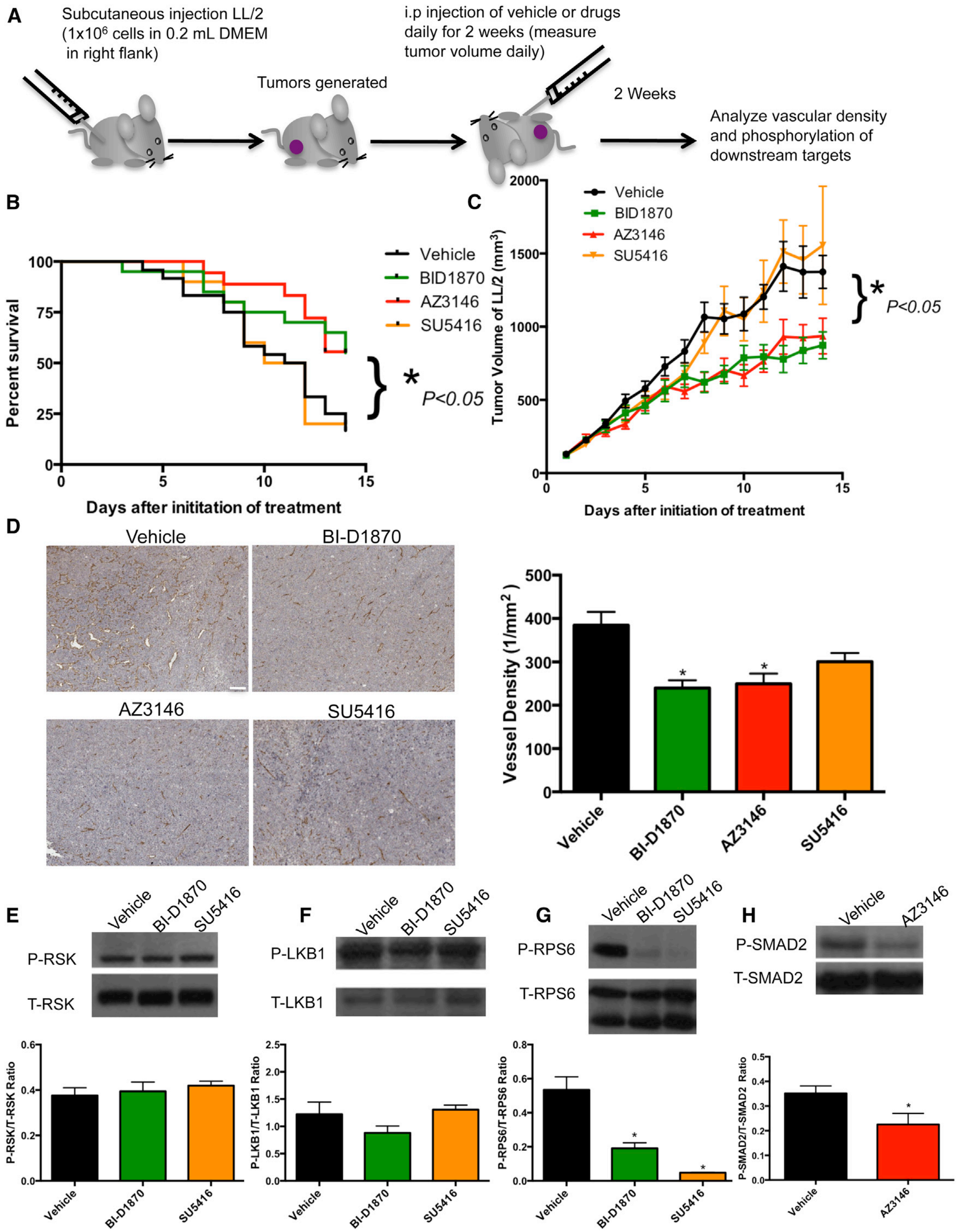

Figure 6. Determination of the Efficacy of BI-D1870 and AZ3146 on a Lewis Lung Tumor Model In Vivo

Lewis lung cells (1×10^6) were injected subcutaneously in the right flank of ~6- to 9-week-old C57Bl/6Crl mice. Mice were randomized into one of four treatment groups: vehicle (n = 24), BI-D1870 (n = 20), AZ3146 (n = 18), and SU5416 (n = 10). Treatments were administered via intraperitoneal (i.p) injection daily for 14 days. Asterisk denotes statistical significance compared with vehicle-treated controls.

(A) Schematic of the methodology for the in vivo study.

(B) Survival was evaluated using the Kaplan-Meier method. *$p < 0.05$.

(legend continued on next page)

density. In contrast, SU5416 had no effect on survival. In agreement with a recent study (Ogawara et al., 2014), SU5416 did not affect LL/2 tumor graft growth nor significantly decreased vessel density. In contrast to Ogawara's and our studies, there is a previous report showing that SU5416 significantly inhibited tumor angiogenesis and metastasis of an LL/2 model (Cuneo et al., 2007). This discrepancy may be due to the fact that Cuneo et al. (2007) began treatment with SU5416 either immediately or 1 hr after injecting LL/2 cells, whereas in our study and that of Ogawara et al. (2014), we began treatment once tumors reached a minimal volume of 100 mm^3. Therefore, in the LL/2 model SU5416 may be effective in inhibiting host vessels from infiltrating the tumor, but ineffective once the tumor is well vascularized. Ogawara et al. (2014) showed that SU5416 significantly reduced tumor growth in B16 and C-26 with no effect on LL/2 tumor grafts. This was attributed to high levels of VEGF within B16 and C-26 tumors, compared with LL/2 tumors, suggesting that VEGF does not play a major role in the angiogenesis of LL/2 tumors; instead, other proangiogenic factors, such as bFGF, are responsible for angiogenesis/tumor growth in LL/2. Other studies showed that inhibition of VEGF in tumors can lead to upregulation of bFGF and other proangiogenic factors (Lu and Bergers, 2013) to overcome VEGF inhibition. Interestingly, RSK is a downstream target of bFGF (Czaplinska et al., 2014) and VEGF (Seko et al., 1998), which constitute two major proangiogenic pathways involved in tumor growth (Lu and Bergers, 2013).

Although many of the signaling events involved in developmental angiogenesis are also involved in tumor angiogenesis, there are distinct differences between these two processes, which lead to dysfunctionality of the tumor vasculature. In the case of tumors, tissue disorganization, high enzymatic activity, overproduction of growth factors and extracellular matrix components, and changes in pH and oxygen in the tumor environment lead to detachment of pericytes, leakiness of vessels, and loss of vascular integrity (Jin and Jakobsson, 2012). This could explain why AZ3146 and BI-D1870 resulted in a significant decrease in vessel density in the tumor but had no effect on the vessel density in normal host tissue. This, along with the lack of change in body weight and behavior, indicates that these drugs at the doses used were not toxic.

We also investigated the effect of AZ3146 and BI-D1870 on the downstream phosphorylation of targets in tumors. AZ3146 significantly decreased SMAD2 phosphorylation in tumors. SMAD2 can positively regulate VEGF release in various tumor cell lines (Seystahl et al., 2015) and plays a role in angiogenesis (Assis et al., 2015; Pen et al., 2008). BI-D1870 had no effect on LKB1 phosphorylation in tumors, unlike EBs. This finding is not surprising, as LKB1 has been shown to promote physiological angiogenesis (Londesborough et al., 2008), whereas in cancer cells it acts as a tumor suppressor and inhibits angiogenesis (Zhuang et al., 2006). However, treatment with BI-D1870 significantly decreased RPS6 phosphorylation in tumors. Interestingly, decreased phosphorylation of RPS6 has been correlated with decreased tumor angiogenesis (Hayashi et al., 2005).

In summary, we have developed and validated a robust vascular differentiation assay from ESCs that can be used to screen for modulators of angiogenesis. This in vitro assay identified RSK and TTK as components of vascular signaling pathways. Inhibition of these pathways in vivo in an LL/2 tumor mouse model increased survival, inhibited tumor growth, and decreased angiogenesis associated with decreased RPS6 and SMAD2 phosphorylation. Extension of this screening approach to a broader spectrum of molecular targets may provide new insights into the regulation of vascular development and uncover potential new targets for the therapeutic modulation of angiogenesis.

EXPERIMENTAL PROCEDURES

Reagents were purchased from Invitrogen unless otherwise specified.

Cell Lines and Culture

Flk1-eGFP mouse ESCs (Ema et al., 2006) were cultured on mitotically inactivated mouse embryonic fibroblasts (MEFs) in

(C) Tumor volume was measured over 14 days. AZ3146 and BI-D1870 resulted in a significant decrease in tumor volume from day 7 and day 8 onward, respectively, compared with vehicle-treated controls. Data are mean ± SEM. $^*p < 0.05$.
(D) Tumors were excised on day 14, fixed, embedded, sectioned, and stained with PECAM-1. Representative images are shown in the left panel (scale bar, 100 μm). Vessel quantification was performed on the majority and in some cases all of the mice/group that survived to the end of the study (day 14 post treatment) and is shown in the graph on the right. Data are mean ± SEM, n = 6 (vehicle), n = 10 (BI-D1870), n = 8 (AZ3146), n = 2 (SU5416). $^*p < 0.01$.
(E–H) Western blot analysis was performed on tumors harvested on day 14 post treatment. (E) Levels of phosphorylated and total RSK. Data are mean ± SEM, p not significant. (F) Levels of phosphorylated and total LKB1. Data are mean ± SEM, p not significant. (G) Levels of phosphorylated and total RPS6. Data are mean ± SEM, $^*p < 0.001$. (H) Levels of phosphorylated and total SMAD2. Data are mean ± SEM, $^*p < 0.05$.
See also Figure S4 and Table S2.

ESC media (ES-DMEM) consisting of high-glucose DMEM, 2 mM GlutaMax, 0.15 mM 1-thioglycerol (Sigma-Aldrich), 0.1 mM nonessential amino acids, 1 mM sodium pyruvate, 1,000 U/mL LIF (Chemicon), 50 U/mL penicillin-streptomycin, and 15% ESC-qualified fetal bovine serum (FBS).

HUVECs were cultured in Medium 200PRF supplemented with the LSGS Kit.

LL/2 cells (ATCC) were maintained in high-glucose DMEM (ATCC) supplemented with 10% FBS.

Optimized Vascular Differentiation Assay and Kinome Inhibitor Screen

MEF-depleted *Flk1-eGFP* cells (10^4 cells/mL) were aggregated in suspension (20-μL hanging drops) for 4 days to form EBs in differentiation media consisting of Iscove's medium (IMDM, Sigma-Aldrich), 1.6 mM GlutaMax, 0.081 mM nonessential amino acids, 0.081 mM 2-mercaptoethanol (Sigma-Aldrich), 10% FBS, 50 U/mL penicillin-streptomycin, and 50 μg/mL ascorbic acid (Sigma-Aldrich). On day 4, EBs were embedded in 2 mg/mL rat tail collagen type I (BD Biosciences) gels in the individual wells of a 96-well plate and incubated at 37°C. The following day differentiation media alone or containing DMSO or VEGF (50 ng/mL, R&D Systems), or VEGF (50 ng/mL) in the presence of other growth factors (100 ng/mL bFGF [R&D]; 10 ng/mL IL-6; and/or 2 U/mL EPO [provided by the Keller laboratory]), or 50 ng/mL VEGF in the presence of 5 μM L685,458 (EMD Biosciences) or 4 μM SU5416 (Sigma-Aldrich) or 2 μM kinase library inhibitors (provided by Ontario Institute for Cancer Research, see Table S3 for the compound list) were added and the media replenished on day 3. The EBs were fixed in 4% formaldehyde (Polysciences) on day 7 (unless otherwise noted) and the number of FLK-1^+ sprouts and total fluorescent intensity were measured using the Cellomics VTI (Zeiss Axio Observer.Z1 microscope, ORCA-ER camera) (Thermo Fisher) platform with a modified neuronal profiling algorithm (see Supplemental Experimental Procedures).

Immunohistochemistry of EBs in collagen gels, dose-curve validation of hits, western blot analysis, HUVEC tube formation assay and disruption of preformed tubes, cytotoxicity assay, and in vivo exposure analysis are described in detail in the Supplemental Experimental Procedures.

Mouse Tumor Grafts and In Vivo Drug Studies

All procedures involving animals were performed in agreement with the Canadian Council for Animal Care (CCAC) guidelines at the Toronto Center for Phenogenomics. Female C57BL/6NCrl mice (~6–9 weeks old) were injected subcutaneously in the right flank with 1×10^6 LL/2 cells in 0.2 mL of serum-free DMEM. Tumor volume was calculated using the formula: $(\text{length} \times \text{width})^2 \times 0.5$. Mice were randomized into one of four treatment groups where the average tumor volume ± SEM per group at the initiation of treatment was as follows: vehicle (129.8 ± 6.6 mm^3, n = 24), BI-D1870 (122 ± 7.6 mm^3, n = 20), AZ3146 (128.6 ± 10.5 mm^3, n = 18), and SU5416 (124.1 ± 6.8 mm^3, n = 10). Treatments were administered via intraperitoneal injection daily for 14 days or until animal endpoint. BI-D1870 and AZ3146 were administered at 50 mg/kg, and SU5416 at 25 mg/kg. Tumors were calipered daily for volume assessment. The experiment was staggered to allow proper handling/monitoring of mice. Mice were euthanized if tumors reached endpoint as outlined by the CCAC (ulcerated tumor, tumor volume ≥1.7 cm^3, or tumor mass = 5% of body weight), or if they displayed poor health. Otherwise mice were euthanized after the last drug dose on day 14. Tumors were excised, and a portion was fixed in 10% formalin and embedded in paraffin. The remainder was cut into pieces and snap-frozen.

Immunohistochemistry and Microvascular Density Quantification of Tumors and Adjacent Normal Tissue

Tumor sections (5 μm), two sections per tumor, were stained with anti-PECAM-1 (M-20) (Santa Cruz Biotechnology). Bound antibody was detected with ImmPRESS (Peroxidase) Polymer anti-rabbit immunoglobulin reagent (Vector Laboratories) and visualized using ImmPACT DAB peroxidase (HRP) substrate (Vector Labs). Mayer's hematoxylin (Sigma-Aldrich) was used as a counterstain. Slides were digitized using an Aperio ScanScope XT scanner (Leica), and computer-aided image analysis was performed and manually checked for quality assurance. Regions of interest were identified with an algorithm that distinguishes tumor from stroma and (peri-)necrotic regions. The vessel density within the tumor region, as well as in the adjacent normal tissue, was quantified using the Definiens Tissue Studio software platform (Definiens). Quantification was done while blinded to the treatment groups.

Statistical Analysis

Statistical analysis was performed using GraphPad Prism, via unpaired Student's t tests, or one-way ANOVA followed by Newman-Keuls post tests. Data are presented as mean ± SEM. Survival was evaluated by the Kaplan-Meier method. n represents the number of independent experiments, unless otherwise noted. $p < 0.05$ was considered statistically significant.

SUPPLEMENTAL INFORMATION

Supplemental Information includes Supplemental Experimental Procedures, four figures, and three tables and can be found with this article online at http://dx.doi.org/10.1016/j.stemcr.2016.08.004.

ACKNOWLEDGMENTS

We thank Alex Manno, Kamal Garcha, Neil Adams, Lauren Beck, Divya Santhanam, Nayasta Kusdaya, Yeji An, Jennifer Du, Leanne Studley, Gessica Raponi, Jodi Garner, and Milan Ganguly for advice and/or technical assistance. We thank the Ontario Institute for Cancer Research and Dr. Gordon Keller for reagents. This study was supported by a Terry Fox New Frontiers Program Project grant from the Canadian Institutes for Health Research and by the Ontario Institute for Cancer Research. L.H. was supported by an Ontario Institute for Regenerative Medicine postdoctoral research award.

Received: March 22, 2016
Revised: August 4, 2016
Accepted: August 5, 2016
Published: September 8, 2016

REFERENCES

Andersson, E.R., and Lendahl, U. (2014). Therapeutic modulation of Notch signalling—are we there yet? Nat. Rev. Drug Discov. *13*, 357–378.

Anjum, R., and Blenis, J. (2008). The RSK family of kinases: emerging roles in cellular signalling. Nat. Rev. Mol. Cell Biol. *9*, 747–758.

Arima, S., Nishiyama, K., Ko, T., Arima, Y., Hakozaki, Y., Sugihara, K., Koseki, H., Uchijima, Y., Kurihara, Y., and Kurihara, H. (2011). Angiogenic morphogenesis driven by dynamic and heterogeneous collective endothelial cell movement. Development *138*, 4763–4776.

Assis, P.A., De Figueiredo-Pontes, L.L., Lima, A.S., Leao, V., Candido, L.A., Pintao, C.T., Garcia, A.B., Saggioro, F.P., Panepucci, R.A., Chahud, F., et al. (2015). Halofuginone inhibits phosphorylation of SMAD-2 reducing angiogenesis and leukemia burden in an acute promyelocytic leukemia mouse model. J. Exp. Clin. Cancer Res. *34*, 65.

Bid, H.K., Zhan, J., Phelps, D.A., Kurmasheva, R.T., and Houghton, P.J. (2012). Potent inhibition of angiogenesis by the IGF-1 receptor-targeting antibody SCH717454 is reversed by IGF-2. Mol. Cancer Ther. *11*, 649–659.

Bono, F., De Smet, F., Herbert, C., De Bock, K., Georgiadou, M., Fons, P., Tjwa, M., Alcouffe, C., Ny, A., Bianciotto, M., et al. (2013). Inhibition of tumor angiogenesis and growth by a small-molecule multi-FGF receptor blocker with allosteric properties. Cancer Cell *23*, 477–488.

Carmeliet, P. (2003). Angiogenesis in health and disease. Nat. Med. *9*, 653–660.

Carmeliet, P., Ferreira, V., Breier, G., Pollefeyt, S., Kieckens, L., Gertsenstein, M., Fahrig, M., Vandenhoeck, A., Harpal, K., Eberhardt, C., et al. (1996). Abnormal blood vessel development and lethality in embryos lacking a single VEGF allele. Nature *380*, 435–439.

Chen, D., Walsh, K., and Wang, J. (2000). Regulation of cdk2 activity in endothelial cells that are inhibited from growth by cell contact. Arterioscler. Thromb. Vasc. Biol. *20*, 629–635.

Chow, L.Q., and Eckhardt, S.G. (2007). Sunitinib: from rational design to clinical efficacy. J. Clin. Oncol. *25*, 884–896.

Chung, N.A., Lydakis, C., Belgore, F., Blann, A.D., and Lip, G.Y. (2002). Angiogenesis in myocardial infarction. An acute or chronic process? Eur. Heart J. *23*, 1604–1608.

Clark, D.E., Errington, T.M., Smith, J.A., Frierson, H.F., Jr., Weber, M.J., and Lannigan, D.A. (2005). The serine/threonine protein kinase, p90 ribosomal S6 kinase, is an important regulator of prostate cancer cell proliferation. Cancer Res. *65*, 3108–3116.

Cuneo, K.C., Fu, A., Osusky, K.L., and Geng, L. (2007). Effects of vascular endothelial growth factor receptor inhibitor SU5416 and prostacyclin on murine lung metastasis. Anticancer Drugs *18*, 349–355.

Czaplinska, D., Turczyk, L., Grudowska, A., Mieszkowska, M., Lipinska, A.D., Skladanowski, A.C., Zaczek, A.J., Romanska, H.M., and Sadej, R. (2014). Phosphorylation of RSK2 at Tyr529 by FGFR2-p38 enhances human mammary epithelial cells migration. Biochim. Biophys. Acta *1843*, 2461–2470.

Di Paolo, D., Ambrogio, C., Pastorino, F., Brignole, C., Martinengo, C., Carosio, R., Loi, M., Pagnan, G., Emionite, L., Cilli, M., et al. (2011). Selective therapeutic targeting of the anaplastic lymphoma kinase with liposomal siRNA induces apoptosis and inhibits angiogenesis in neuroblastoma. Mol. Ther. *19*, 2201–2212.

Ding, L., and Buchholz, F. (2006). RNAi in embryonic stem cells. Stem Cell Rev. *2*, 11–18.

Duan, H.F., Wang, H., Yi, J., Liu, H.J., Zhang, Q.W., Li, L.B., Zhang, T., Lu, Y., Wu, C.T., and Wang, L.S. (2007). Adenoviral gene transfer of sphingosine kinase 1 protects heart against ischemia/reperfusion-induced injury and attenuates its postischemic failure. Hum. Gene Ther. *18*, 1119–1128.

Eklund, L., Bry, M., and Alitalo, K. (2013). Mouse models for studying angiogenesis and lymphangiogenesis in cancer. Mol. Oncol. *7*, 259–282.

Ema, M., Takahashi, S., and Rossant, J. (2006). Deletion of the selection cassette, but not cis-acting elements, in targeted Flk1-lacZ allele reveals Flk1 expression in multipotent mesodermal progenitors. Blood *107*, 111–117.

Feraud, O., Cao, Y., and Vittet, D. (2001). Embryonic stem cell-derived embryoid bodies development in collagen gels recapitulates sprouting angiogenesis. Lab. Invest. *81*, 1669–1681.

Ferrara, N. (2004). Vascular endothelial growth factor as a target for anticancer therapy. Oncologist *9* (*Suppl 1*), 2–10.

Ferrara, N., Hillan, K.J., and Novotny, W. (2005). Bevacizumab (Avastin), a humanized anti-VEGF monoclonal antibody for cancer therapy. Biochem. Biophys. Res. Commun. *333*, 328–335.

Fong, T.A., Shawver, L.K., Sun, L., Tang, C., App, H., Powell, T.J., Kim, Y.H., Schreck, R., Wang, X., Risau, W., et al. (1999). SU5416 is a potent and selective inhibitor of the vascular endothelial growth factor receptor (Flk-1/KDR) that inhibits tyrosine kinase catalysis, tumor vascularization, and growth of multiple tumor types. Cancer Res. *59*, 99–106.

Fryer, R.M., Muthukumarana, A., Chen, R.R., Smith, J.D., Mazurek, S.N., Harrington, K.E., Dinallo, R.M., Burke, J., DiCapua, F.M., Guo, X., et al. (2012). Mitigation of off-target adrenergic binding and effects on cardiovascular function in the discovery of novel ribosomal S6 kinase 2 inhibitors. J. Pharmacol. Exp. Ther. *340*, 492–500.

Giroux, S., Tremblay, M., Bernard, D., Cardin-Girard, J.F., Aubry, S., Larouche, L., Rousseau, S., Huot, J., Landry, J., Jeannotte, L., et al. (1999). Embryonic death of Mek1-deficient mice reveals a role for this kinase in angiogenesis in the labyrinthine region of the placenta. Curr. Biol. *9*, 369–372.

Gomes, C.P., Gomes-da-Silva, L.C., Ramalho, J.S., de Lima, M.C., Simoes, S., and Moreira, J.N. (2013). Impact of PLK-1 silencing on endothelial cells and cancer cells of diverse histological origin. Curr. Gene Ther. *13*, 189–201.

Hayashi, M., Fearns, C., Eliceiri, B., Yang, Y., and Lee, J.D. (2005). Big mitogen-activated protein kinase 1/extracellular signal-regulated kinase 5 signaling pathway is essential for tumor-associated angiogenesis. Cancer Res. *65*, 7699–7706.

Hellstrom, M., Phng, L.K., Hofmann, J.J., Wallgard, E., Coultas, L., Lindblom, P., Alva, J., Nilsson, A.K., Karlsson, L., Gaiano, N., et al.

(2007). Dll4 signalling through Notch1 regulates formation of tip cells during angiogenesis. Nature *445*, 776–780.

Hermant, B., Desroches-Castan, A., Dubessay, M.L., Prandini, M.H., Huber, P., and Vittet, D. (2007). Development of a one-step embryonic stem cell-based assay for the screening of sprouting angiogenesis. BMC Biotechnol. *7*, 20.

Hewitt, L., Tighe, A., Santaguida, S., White, A.M., Jones, C.D., Musacchio, A., Green, S., and Taylor, S.S. (2010). Sustained Mps1 activity is required in mitosis to recruit O-Mad2 to the Mad1-C-Mad2 core complex. J. Cell Biol. *190*, 25–34.

Jadrich, J.L., O'Connor, M.B., and Coucouvanis, E. (2006). The TGF beta activated kinase TAK1 regulates vascular development in vivo. Development *133*, 1529–1541.

Jakobsson, L., Kreuger, J., Holmborn, K., Lundin, L., Eriksson, I., Kjellen, L., and Claesson-Welsh, L. (2006). Heparan sulfate in trans potentiates VEGFR-mediated angiogenesis. Dev. Cell *10*, 625–634.

Jeong, W., Kim, J., Bazer, F.W., and Song, G. (2014). Stimulatory effect of vascular endothelial growth factor on proliferation and migration of porcine trophectoderm cells and their regulation by the phosphatidylinositol-3-kinase-akt and mitogen-activated protein kinase cell signaling pathways. Biol. Reprod. *90*, 50.

Jin, Y., and Jakobsson, L. (2012). The dynamics of developmental and tumor angiogenesis-a comparison. Cancers (Basel) *4*, 400–419.

Kazerounian, S., Duquette, M., Reyes, M.A., Lawler, J.T., Song, K., Perruzzi, C., Primo, L., Khosravi-Far, R., Bussolino, F., Rabinovitz, I., et al. (2011). Priming of the vascular endothelial growth factor signaling pathway by thrombospondin-1, CD36, and spleen tyrosine kinase. Blood *117*, 4658–4666.

Keller, G. (2005). Embryonic stem cell differentiation: emergence of a new era in biology and medicine. Genes Dev. *19*, 1129–1155.

Kerbel, R.S. (2008). Tumor angiogenesis. N. Engl. J. Med. *358*, 2039–2049.

Kuhnert, F., Kirshner, J.R., and Thurston, G. (2011). Dll4-Notch signaling as a therapeutic target in tumor angiogenesis. Vasc. Cell *3*, 20.

Kerr, G., Sheldon, H., Chaikuad, A., Alfano, I., von Delft, F., Bullock, A.N., and Harris, A.L. (2015). A small molecule targeting ALK1 prevents Notch cooperativity and inhibits functional angiogenesis. Angiogenesis *18*, 209–217.

Liu, X., and Winey, M. (2012). The MPS1 family of protein kinases. Annu. Rev. Biochem. *81*, 561–585.

Londesborough, A., Vaahtomeri, K., Tiainen, M., Katajisto, P., Ekman, N., Vallenius, T., and Makela, T.P. (2008). LKB1 in endothelial cells is required for angiogenesis and TGFbeta-mediated vascular smooth muscle cell recruitment. Development *135*, 2331–2338.

Lu, K.V., and Bergers, G. (2013). Mechanisms of evasive resistance to anti-VEGF therapy in glioblastoma. CNS Oncol. *2*, 49–65.

Ogawara, K., Abe, S., Un, K., Yoshizawa, Y., Kimura, T., and Higaki, K. (2014). Determinants for in vivo antitumor effect of angiogenesis inhibitor SU5416 formulated in PEGylated emulsion. J. Pharm. Sci. *103*, 2464–2469.

Partanen, J., Puri, M.C., Schwartz, L., Fischer, K.D., Bernstein, A., and Rossant, J. (1996). Cell autonomous functions of the receptor tyrosine kinase TIE in a late phase of angiogenic capillary growth and endothelial cell survival during murine development. Development *122*, 3013–3021.

Pen, A., Moreno, M.J., Durocher, Y., Deb-Rinker, P., and Stanimirovic, D.B. (2008). Glioblastoma-secreted factors induce IGFBP7 and angiogenesis by modulating Smad-2-dependent TGF-beta signaling. Oncogene *27*, 6834–6844.

Pi, X., Garin, G., Xie, L., Zheng, Q., Wei, H., Abe, J., Yan, C., and Berk, B.C. (2005). BMK1/ERK5 is a novel regulator of angiogenesis by destabilizing hypoxia inducible factor 1alpha. Circ. Res. *96*, 1145–1151.

Roffe, M., Lupinacci, F.C., Soares, L.C., Hajj, G.N., and Martins, V.R. (2015). Two widely used RSK inhibitors, BI-D1870 and SL0101, alter mTORC1 signaling in a RSK-independent manner. Cell Signal. *27*, 1630–1642.

Romain, C., Paul, P., Kim, K.W., Lee, S., Qiao, J., and Chung, D.H. (2014). Targeting Aurora kinase-A downregulates cell proliferation and angiogenesis in neuroblastoma. J. Pediatr. Surg. *49*, 159–165.

Romeo, Y., Zhang, X., and Roux, P.P. (2012). Regulation and function of the RSK family of protein kinases. Biochem. J. *441*, 553–569.

Rossant, J., and Howard, L. (2002). Signaling pathways in vascular development. Annu. Rev. Cell Dev. Biol. *18*, 541–573.

Ruiter, D.J., Schlingemann, R.O., Westphal, J.R., Denijn, M., Rietveld, F.J., and De Waal, R.M. (1993). Angiogenesis in wound healing and tumor metastasis. Behring Inst. Mitt., 258–272.

Safina, A., Vandette, E., and Bakin, A.V. (2007). ALK5 promotes tumor angiogenesis by upregulating matrix metalloproteinase-9 in tumor cells. Oncogene *26*, 2407–2422.

Sapkota, G.P., Cummings, L., Newell, F.S., Armstrong, C., Bain, J., Frodin, M., Grauert, M., Hoffmann, M., Schnapp, G., Steegmaier, M., et al. (2007). BI-D1870 is a specific inhibitor of the p90 RSK (ribosomal S6 kinase) isoforms in vitro and in vivo. Biochem. J. *401*, 29–38.

Schenone, S., Manetti, F., and Botta, M. (2007). SRC inhibitors and angiogenesis. Curr. Pharm. Des. *13*, 2118–2128.

Seko, Y., Takahashi, N., Tobe, K., Ueki, K., Kadowaki, T., and Yazaki, Y. (1998). Vascular endothelial growth factor (VEGF) activates Raf-1, mitogen-activated protein (MAP) kinases, and S6 kinase (p90rsk) in cultured rat cardiac myocytes. J. Cell Physiol. *175*, 239–246.

Seystahl, K., Tritschler, I., Szabo, E., Tabatabai, G., and Weller, M. (2015). Differential regulation of TGF-beta-induced, ALK-5-mediated VEGF release by SMAD2/3 versus SMAD1/5/8 signaling in glioblastoma. Neuro Oncol. *17*, 254–265.

Shalaby, F., Rossant, J., Yamaguchi, T.P., Gertsenstein, M., Wu, X.F., Breitman, M.L., and Schuh, A.C. (1995). Failure of blood-island formation and vasculogenesis in Flk-1-deficient mice. Nature *376*, 62–66.

Shi, N., Xie, W.B., and Chen, S.Y. (2012). Cell division cycle 7 is a novel regulator of transforming growth factor-beta-induced smooth muscle cell differentiation. J. Biol. Chem. *287*, 6860–6867.

Sleijfer, S., Ray-Coquard, I., Papai, Z., Le Cesne, A., Scurr, M., Schoffski, P., Collin, F., Pandite, L., Marreaud, S., De Brauwer, A., et al. (2009). Pazopanib, a multikinase angiogenesis inhibitor, in

patients with relapsed or refractory advanced soft tissue sarcoma: a phase II study from the European organisation for research and treatment of cancer-soft tissue and bone sarcoma group (EORTC study 62043). J. Clin. Oncol. *27*, 3126–3132.

Smith, J.A., Poteet-Smith, C.E., Xu, Y., Errington, T.M., Hecht, S.M., and Lannigan, D.A. (2005). Identification of the first specific inhibitor of p90 ribosomal S6 kinase (RSK) reveals an unexpected role for RSK in cancer cell proliferation. Cancer Res. *65*, 1027–1034.

Solter, D. (2006). From teratocarcinomas to embryonic stem cells and beyond: a history of embryonic stem cell research. Nat. Rev. Genet. *7*, 319–327.

Srinivasan, R., Zabuawala, T., Huang, H., Zhang, J., Gulati, P., Fernandez, S., Karlo, J.C., Landreth, G.E., Leone, G., and Ostrowski, M.C. (2009). Erk1 and Erk2 regulate endothelial cell proliferation and migration during mouse embryonic angiogenesis. PLoS One *4*, e8283.

Tavora, B., Batista, S., Reynolds, L.E., Jadeja, S., Robinson, S., Kostourou, V., Hart, I., Fruttiger, M., Parsons, M., and Hodivala-Dilke, K.M. (2010). Endothelial FAK is required for tumour angiogenesis. EMBO Mol. Med. *2*, 516–528.

Tawaramoto, K., Kotani, K., Hashiramoto, M., Kanda, Y., Nagare, T., Sakaue, H., Ogawa, W., Emoto, N., Yanagisawa, M., Noda, T., et al. (2012). Ablation of 3-phosphoinositide-dependent protein kinase 1 (PDK1) in vascular endothelial cells enhances insulin sensitivity by reducing visceral fat and suppressing angiogenesis. Mol. Endocrinol. *26*, 95–109.

Wimmer, R., Cseh, B., Maier, B., Scherrer, K., and Baccarini, M. (2012). Angiogenic sprouting requires the fine tuning of endothelial cell cohesion by the Raf-1/Rok-alpha complex. Dev. Cell *22*, 158–171.

Xin, H., Herrmann, A., Reckamp, K., Zhang, W., Pal, S., Hedvat, M., Zhang, C., Liang, W., Scuto, A., Weng, S., et al. (2011). Antiangiogenic and antimetastatic activity of JAK inhibitor AZD1480. Cancer Res. *71*, 6601–6610.

Xu, Y., Shi, Y., and Ding, S. (2008). A chemical approach to stem-cell biology and regenerative medicine. Nature *453*, 338–344.

Zhang, J., Cao, R., Zhang, Y., Jia, T., Cao, Y., and Wahlberg, E. (2009). Differential roles of PDGFR-alpha and PDGFR-beta in angiogenesis and vessel stability. FASEB J. *23*, 153–163.

Zhu, S., Wang, W., Clarke, D.C., and Liu, X. (2007). Activation of Mps1 promotes transforming growth factor-beta-independent Smad signaling. J. Biol. Chem. *282*, 18327–18338.

Zhuang, Z.G., Di, G.H., Shen, Z.Z., Ding, J., and Shao, Z.M. (2006). Enhanced expression of LKB1 in breast cancer cells attenuates angiogenesis, invasion, and metastatic potential. Mol. Cancer Res. *4*, 843–849.

Stem Cell Reports, Volume 7

Supplemental Information

Identification of RSK and TTK as Modulators of Blood Vessel Morphogenesis Using an Embryonic Stem Cell-Based Vascular Differentiation Assay

Lamis Hammoud, Jessica R. Adams, Amanda J. Loch, Richard C. Marcellus, David E. Uehling, Ahmed Aman, Christopher Fladd, Trevor D. McKee, Christine E.B. Jo, Rima Al-Awar, Sean E. Egan, and Janet Rossant

Supplemental Figures

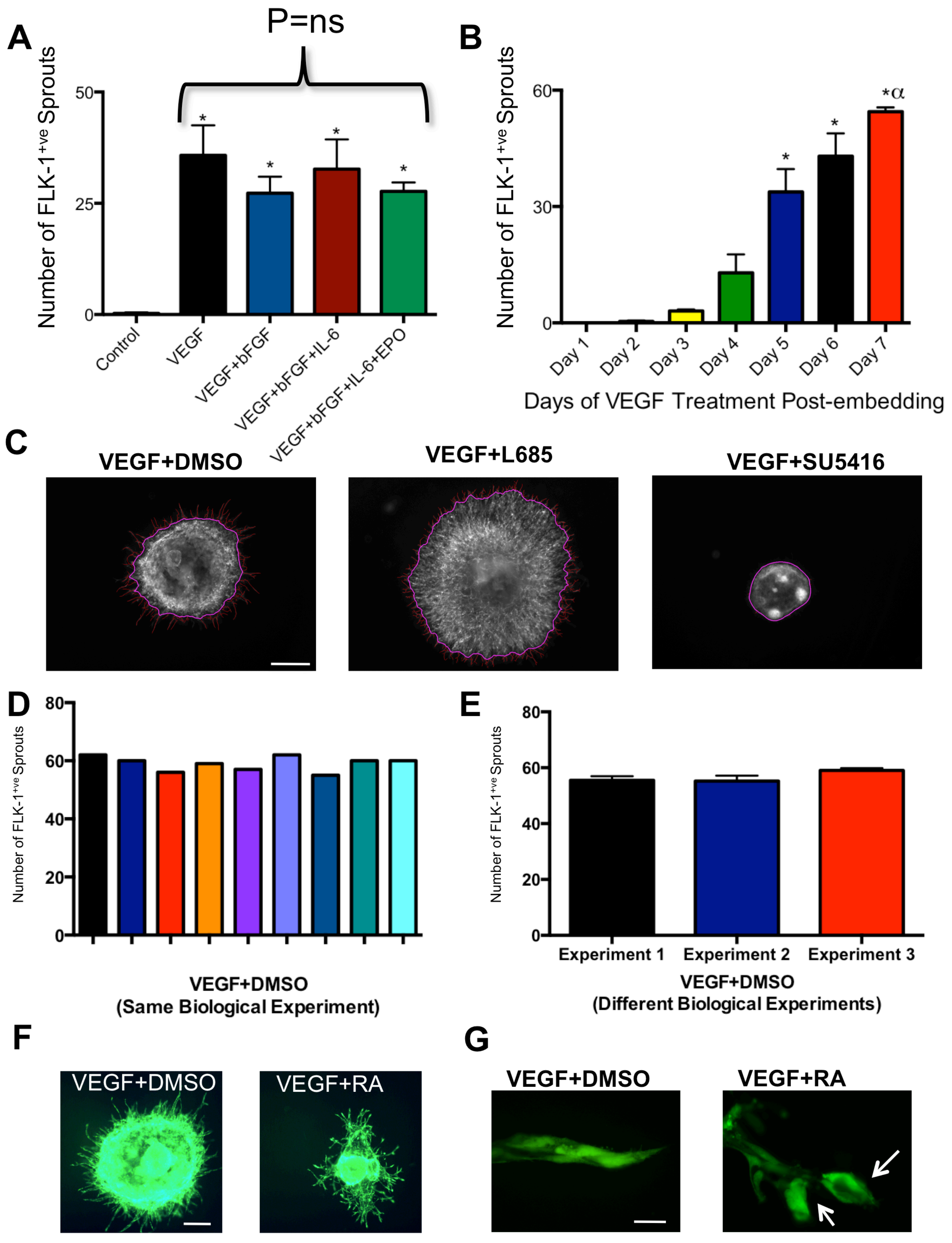

Figure S1

Hits Analysis

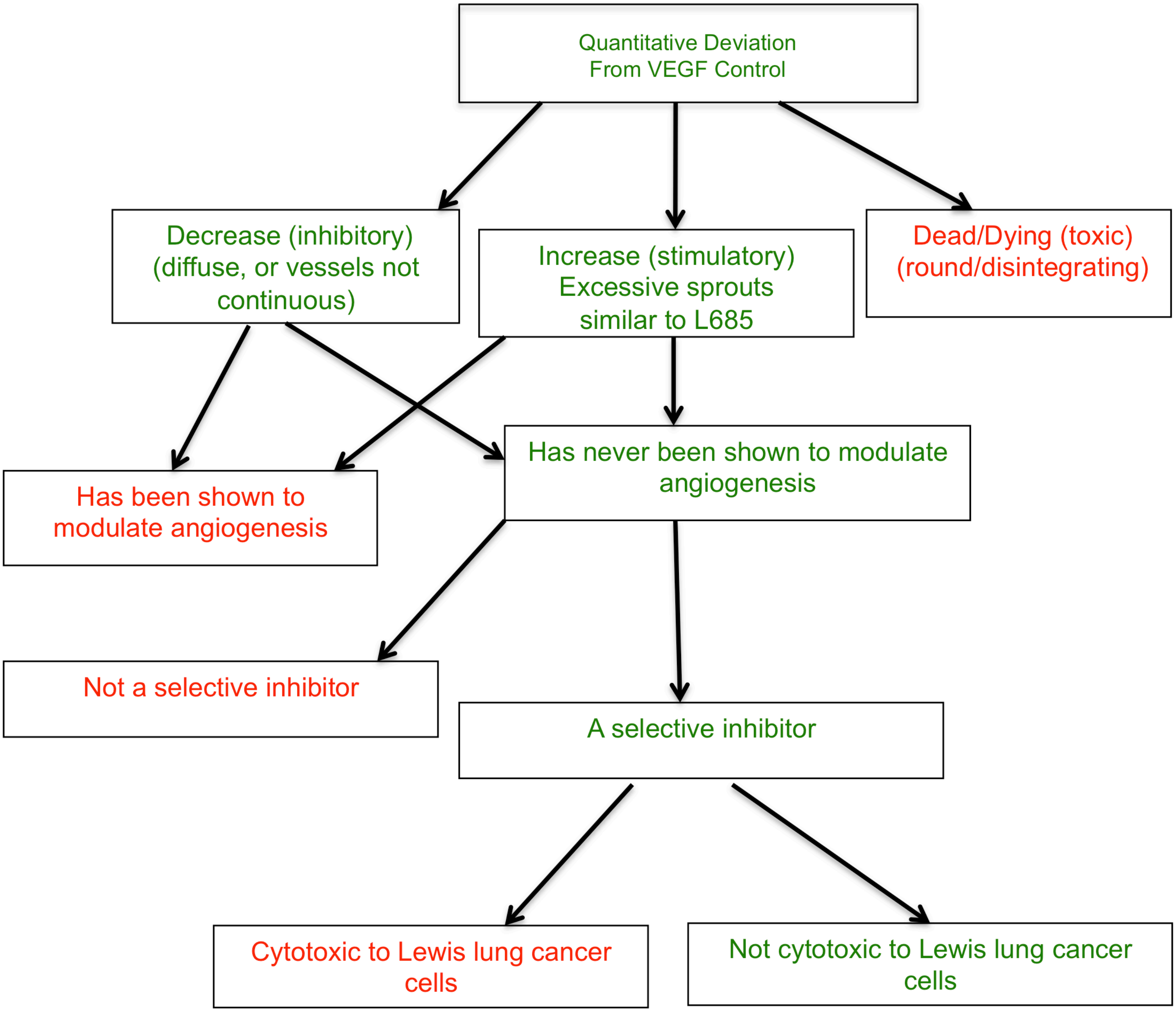

Figure S2

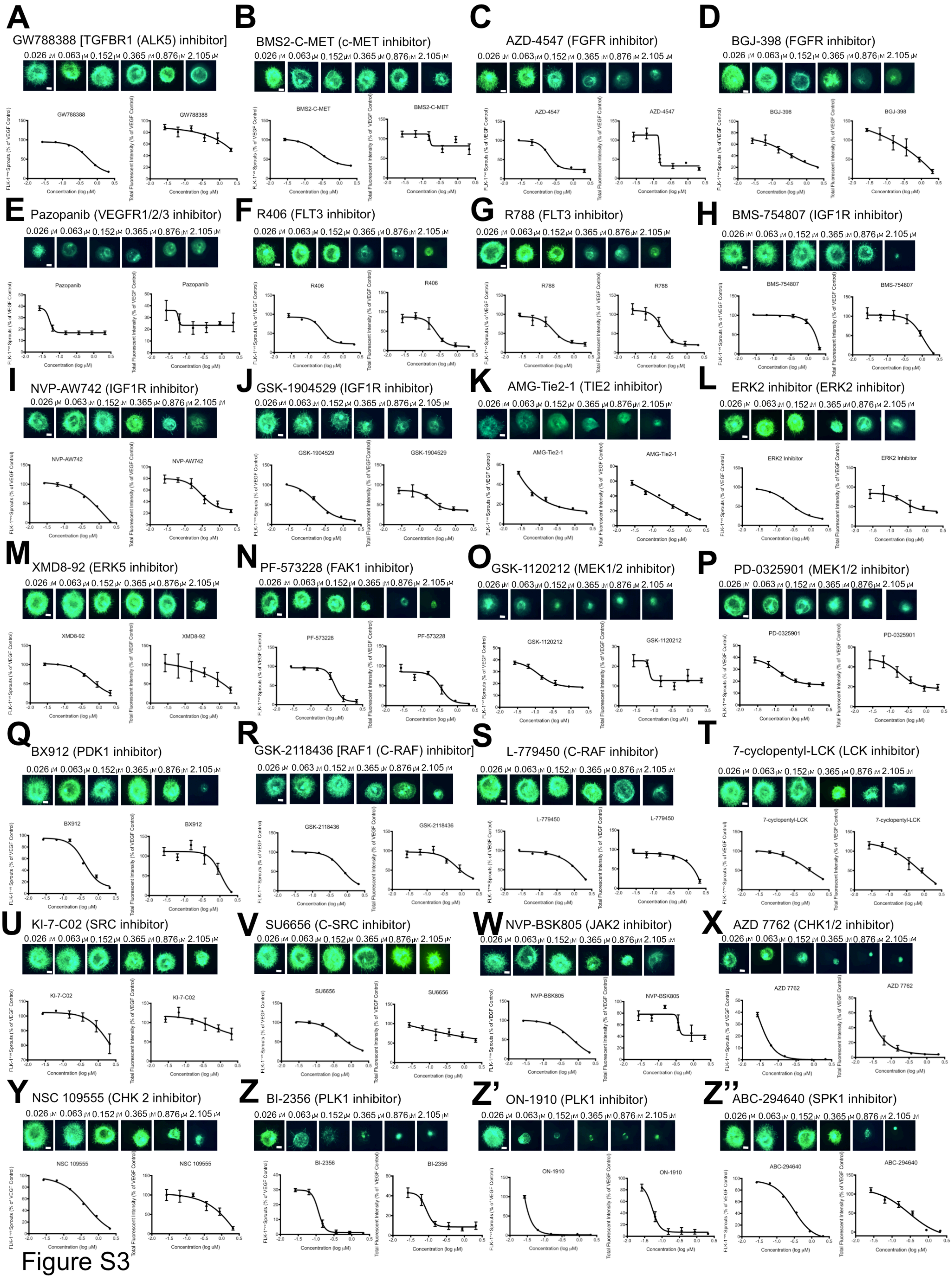
A GW788388 [TGFBR1 (ALK5) inhibitor]
B BMS2-C-MET (c-MET inhibitor)
C AZD-4547 (FGFR inhibitor)
D BGJ-398 (FGFR inhibitor)
E Pazopanib (VEGFR1/2/3 inhibitor)
F R406 (FLT3 inhibitor)
G R788 (FLT3 inhibitor)
H BMS-754807 (IGF1R inhibitor)
I NVP-AW742 (IGF1R inhibitor)
J GSK-1904529 (IGF1R inhibitor)
K AMG-Tie2-1 (TIE2 inhibitor)
L ERK2 inhibitor (ERK2 inhibitor)
M XMD8-92 (ERK5 inhibitor)
N PF-573228 (FAK1 inhibitor)
O GSK-1120212 (MEK1/2 inhibitor)
P PD-0325901 (MEK1/2 inhibitor)
Q BX912 (PDK1 inhibitor)
R GSK-2118436 [RAF1 (C-RAF) inhibitor]
S L-779450 (C-RAF inhibitor)
T 7-cyclopentyl-LCK (LCK inhibitor)
U KI-7-C02 (SRC inhibitor)
V SU6656 (C-SRC inhibitor)
W NVP-BSK805 (JAK2 inhibitor)
X AZD 7762 (CHK1/2 inhibitor)
Y NSC 109555 (CHK 2 inhibitor)
Z BI-2356 (PLK1 inhibitor)
Z' ON-1910 (PLK1 inhibitor)
Z'' ABC-294640 (SPK1 inhibitor)
0.026 μM 0.063 μM 0.152 μM 0.365 μM 0.876 μM 2.105 μM
FLK-1+ve Sprouts (% of VEGF Control)
Total Fluorescent Intensity (% of VEGF Control)
Concentration (log μM)

Figure S3

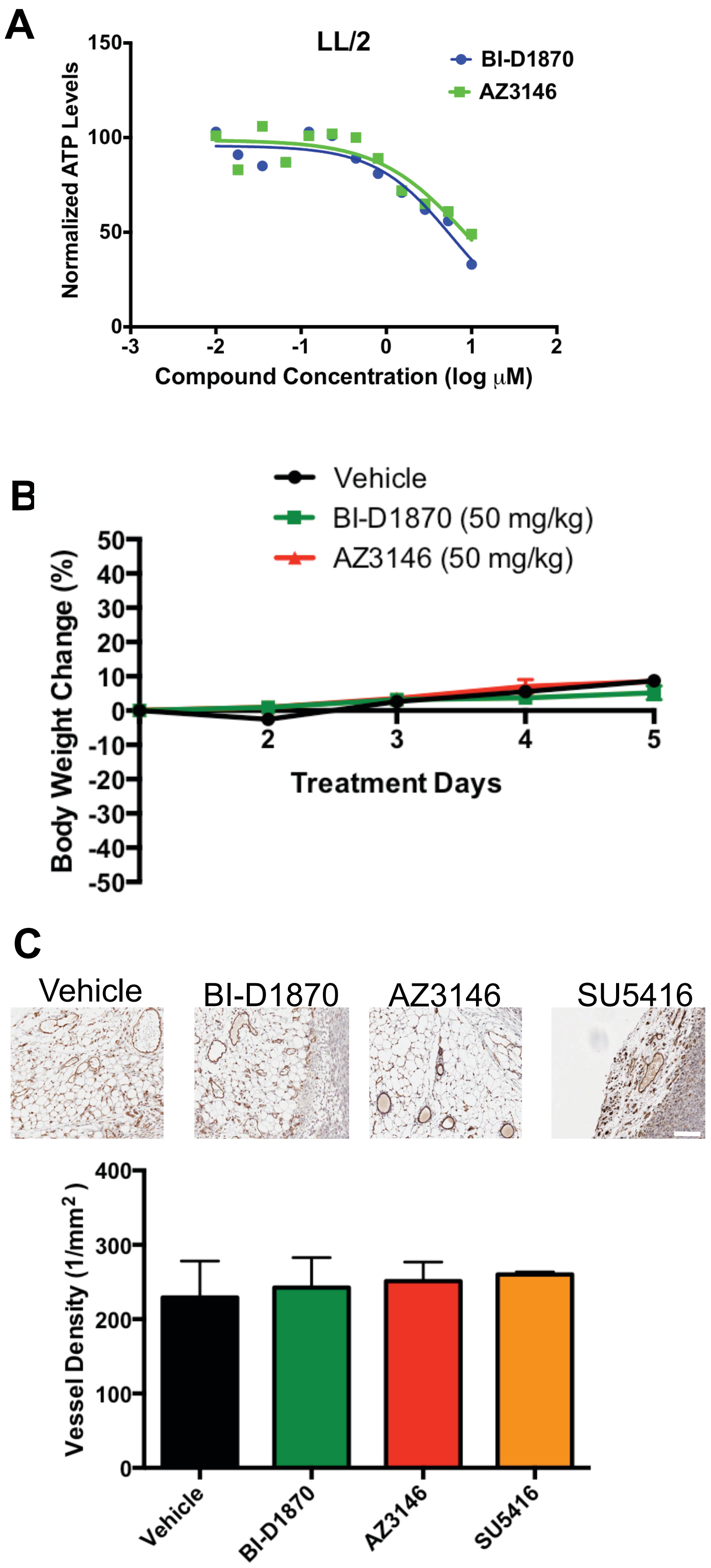

A
LL/2
BI-D1870
AZ3146
Normalized ATP Levels
150
100
50
0
-3
-2
-1
0
1
2
Compound Concentration (log µM)
B
Vehicle
BI-D1870 (50 mg/kg)
AZ3146 (50 mg/kg)
Body Weight Change (%)
50
40
30
20
10
0
-10
-20
-30
-40
-50
2
3
4
5
Treatment Days
C
Vehicle
BI-D1870
AZ3146
SU5416
Vessel Density (1/mm²)
400
300
200
100
0
Vehicle
BI-D1870
AZ3146
SU5416

Figure S4

Supplemental Figure Legends

Figure S1 (related to Figure 1). Effect of growth factor treatment on EB sprouting following embedding in collagen type I gels, determination of the optimal time point for sprout quantification, automated quantification using Cellomics Neuronal Profiling BioApplication, reproducibility of the VEGF+DMSO control in the same and different biological experiments, and sensitivity of our assay for detection of morphological changes in vessel shape. *Flk1-eGFP* ESCs were aggregated in suspension for 4 days as hanging drops and then embedded in a collagen type I matrix. (A) EBs were grown in differentiation media alone (control) or containing VEGF (50 ng/mL), or containing VEGF (50 ng/mL) in the presence of other growth factors (100 ng/mL bFGF; 10 ng/mL IL-6; 2 U/mL EPO). The number of FLK-1 positive sprouts were quantified on day 6 post-embedding. Data are mean ± SEM, N≥3 $P<0.0001$. Star (*) denotes statistical significance compared to control. P=ns for VEGF vs other growth factors. (B) EBs were grown in media containing VEGF (50 ng/ml) and FLK-1 positive sprouts were quantified each day for 7 days post-embedding. N=3, data are mean ± SEM. Star (*) denotes statistical significance compared to day 1,2,3 and 4, and α denotes statistical significance compared to day 5, and 6. (C) Representative images acquired using the Cellomics VTI platform and quantified using the Neuronal Profiling BioApplication. Red denotes FLK-1 positive sprouts quantified by the algorithm. (D-E) EBs were grown in media containing VEGF (50 ng/ml)+DMSO and FLK-1 positive sprouts were quantified on day 7 post-embedding. (D) Reproducibility of the VEGF+DMSO control between technical replicates. (E) Reproducibility of the VEGF+DMSO control between independent experiments (biological replicates). Data are mean ± SEM, N=3, P=ns. (F-G) Treatment with 2 μM all-trans retinoic acid (RA) resulted in ballooning of the vascular sprouts. Scale bars were 300 μm and 25 μm for C and D respectively. White arrows point to ballooned structures.

Figure S2 (related to Figure 2). Hit analysis chart. For a hit to be worth pursing, the compound should be selective and have established potency (<50 nM) against the kinase target of interest, as well as have novelty with respect to angiogenesis as an indicated use. Additionally, the compound must not have been reported to be cytotoxic to Lewis lung carcinoma cell line (our tumor model of choice). Green indicates worth pursuing further, red indicates not worth pursing further.

Figure S3 (related to Figure 2 and Table 1). Dose curve analysis of ALK5, c-MET, FGFR, VEGFR1/2/3, FLT3, IGF1R, TIE2, ERK2, ERK5, FAK1, MEK1/2, PDK1, RAF1/c-RAF, LCK, SRC, JAK2, CHK1/2, PLK1 and SPK1 inhibition. *Flk1-eGFP* ESCs were aggregated in suspension for 4 days as hanging drops and then embedded in a collagen type I matrix, one EB per well of 96 well plate. The following day EBs were treated with DMSO, VEGF (50 ng/ml) in the presence of DMSO, or VEGF (50 ng/ml) in the presence of varying concentrations of compounds (0.026 μM-2.1 μM), and dosed twice over a 7 day period. On day 7 post-embedding, EBs were fixed, imaged and the number of FLK-1 positive sprouts and total fluorescent intensity were quantified. Values were normalized to VEGF control. Drug doses were Log transformed. Data are mean ± SEM, N=4 technical replicates. For A-Z", scale bar, 300 μm. (A) Dose curve analysis of GW788388 (ALK5 inhibitor). (B) Dose curve analysis of BMS2-C-MET (c-MET inhibitor). (C) Dose curve analysis of AZD-4547 (FGFR inhibitor). (D) Dose curve analysis of BGJ-398 (FGFR inhibitor). (E) Dose curve analysis of Pazopanib (VEGFR1/2/3 inhibitor). (F) Dose curve analysis of R406 (FLT3 inhibitor). (G) Dose curve analysis of R788 (FLT3 inhibitor). (H) Dose curve analysis of BMS-754807 (IGF1R inhibitor). (I) Dose curve analysis of NVP-AW742 (IGF1R inhibitor). (J) Dose curve analysis of GSK-1904529 (IGF1R inhibitor). (K) Dose curve analysis of AMG-Tie2-1 (TIE2 inhibitor). (L) Dose curve analysis of ERK2 inhibitor (ERK2 inhibitor). (M) Dose curve analysis of XMD8-92 (ERK5 inhibitor). (N) Dose curve analysis of PF-573228 (FAK1 inhibitor). (O) Dose curve analysis of GSK-1120212 (MEK1/2 inhibitor). (P) Dose curve analysis of PD-0325901 (MEK1/2 inhibitor). (Q) Dose curve analysis of BX912 (PDK1 inhibitor). (R) Dose curve analysis of GSK-2118436 (RAF1/c-RAF inhibitor). (S) Dose curve analysis of L-779450 (c-RAF inhibitor). (T) Dose curve analysis of 7-cyclopentyl-LCK (LCK inhibitor). (U) Dose curve analysis of KI-7-C02 (SRC inhibitor). (V) Dose curve analysis of SU6656 (c-SRC inhibitor). (W) Dose curve analysis of NVP-BSK805 (JAK2 inhibitor). (X) Dose curve analysis of AZD 7762 (CHK1/2 inhibitor). (Y) Dose curve analysis of NSC 109555 (CHK2 inhibitor). (Z) Dose curve

analysis of BI-2356 (PLK1 inhibitor). (Z') Dose curve analysis of ON-1910 (PLK1 inhibitor). (Z") Dose curve analysis of ABC-294640 (SPK1 inhibitor).

Figure S4 (related to Figure 6 and Table S2). Determination of whether BI-D1870 or AZ3146 have cytotoxic effects on Lewis lung tumor cells *in vitro* and whether these drugs have toxic effects *in vivo.* Lewis lung cells were plated in 96 well plates and treated with increasing doses (0-10 μM) of BI-D1870 or AZ3146, 24 hours post-plating. ATP quantification, measured as chemiluminescence was performed 72 hours post-plating and data were normalized to DMSO controls. Drug doses were Log transformed. (B) During *in vivo* exposure analysis, mice were treated with either vehicle, 50 mg/kg BI-D1870 or 50 mg/kg AZ3146 via intraperitoneal (i.p) injection for 5 days, no significant change in body weight was observed between vehicle and drug treated mice. Data are mean± SEM, N=3 mice/group, *P*=ns. (C) PECAM-1 staining and quantification of the vessel density in the normal tissue, adjacent to the tumors, showed no significant difference between vehicle treated and drug treated groups. Scale bar, 100 μm. Data are mean ± SEM, N=6 (vehicle), N=10 (BI-D1870), N=8 (AZ3146), N=2 (SU5416), *P*=ns.

Supplemental Tables

Table S1 (related to Figure 2). Inhibitors with off-target effects.

Compound	Target	Reason considered off-target
TX-1918	eEF2K	These eF2K inhibitors are known to have off-target effects
NH125	eEF2K	
BIBU 1361 dihydrochloride	EGFR (ERBB1, HER1)	Only 6 out of 34 EGFR inhibitors showed angiogeneic inhibition. Inhibition observed with AEE 788 treatment is likely due to inhibition of Flk1 not EGFR. Similarly inhibition observed with Vandetanib treatment is likely due to targeting KDR/KIT/FLT1/ FLT4 and not EGFR.
WZ3146, WZ-3146	EGFR (ERBB1, HER1)	
BMS-599626, AC-480	EGFR (ERBB1, HER1), ERBB2 (TKR1, HER2, NEU), ERBB3 (HER3), ERBB4 (HER4)	
Dacomitinib hydrate, PF-00299804, PF-00299804-03, PF-299, PF-299804	EGFR (ERBB1, HER1), ERBB2 (TKR1, HER2, NEU), ERBB4 (HER4)	
AEE 788, AEE-788, NVP-AEE-788	EGFR (ERBB1, HER1), ERBB2 (TKR1, HER2, NEU), KDR (VEGFR2, VEGFR, FLK1)	
Vandetanib, Zactima, ZD6474, AZD-6474	EGFR (ERBB1, HER1), KDR (VEGFR2, VEGFR, FLK1), RET, ABL1 (ABL), KIT (c-KIT), FLT1 (VEGFR1), FLT4 (VEGFR3), TRKA (TRK)	
6-bromoindirubin-3'-oxime, BIO	GSK3	Only 3 out of 10 GSK3 inhibitors showed angiogenic inhibition. The more selective GSK inhibitors did not show this effect.
TWS-119	GSK3	
A 1070722, A-1070722	GSK3	
BIRB 796, Doramapimod	p38 MAPK	Only 2 out of 21 p38 inhibitors showed angiogenic inhibition. The more specific p38 inhibitors did not show this effect
LY2228820, LY-2228820	p38a, p38b	
RHO-15	ROCK1, ROCK2 (ROCKa)	Only 2 out of 10 ROCK inhibitors showed angiogenic inhibition. The more specific ROCK inhibitors did not show this effect
Rho Kinase Inhibitor V	ROCK1, ROCK2 (ROCKa)	
ML 9 hydrochloride	smMLCK (MLCK, myosin light chain kinase, MYLK)	This inhibitor is non-specific and only weakly active against MLCK
AT7867, AT-7867	AKT1 (PKBa), AKT2 (PKBb), AKT3 (PKBg), p70S6K (S6K, S6K1)	These few inhibitors resulted in angiogenic increases, however they are known to be non-specific, and the majority of the inhibitors targeting these kinases in the library did not cause this effect.
Enzastaurin, LY-317615	AKT1 (PKBa), PKCb, p70S6K (S6K, S6K1), GSK3B	
SB 216763	GSK3	
SU-11274, PKI-SU11274	MET (c-MET, HGFR)	
Bisindolylmaleimide I hydrochloride, GF 109203X HCl, Go 6850 HCl	PKC	
Bisindoylmaleimide X, HCl salt	PKC	

Compounds, their respective targets and reason considered off-target have been colour-coded

Table S2 (related to Figure 3, Figure 6, and Figure S4). Selectivity of BI-D1870 and AZ3146 for inhibition of EB angiogenic sprouting over having any effect on Lewis lung cells.

In Vivo Drug	*In Vivo* Plasma Concentrations: 60 min	*In Vivo* Plasma Concentrations: 120 min	*In Vitro* Drug	*In Vitro* IC50s: IC_{50} EBs	*In Vitro* IC50s: IC_{50} (LL/2)	Fold Selectivity(IC_{50} LL2/IC_{50} of EBs)
BI-D1870 (50 mg/kg) i.p	2.81 µM	0.841 µM	BI-D1870	0.16 µM	5.32 µM	33.25
AZ3146 (50 mg/kg) i.p	3.12 µM	1.434 µM	AZ3146	0.72 µM	8.32 µM	11.55

50 mg/kg i.p of BI-D1870 and AZ3146 yielded plasma concentration levels well above the IC_{50} required to inhibit EB angiogenesis and well below the IC_{50} of having any effect on LL/2 cells.

Supplemental Experimental Procedures

Reagents were purchased from Invitrogen unless otherwise specified.

Conditions tested for optimization of vascular differentiation assay
MEF-depleted *Flk1-eGFP* cells were aggregated in suspension at the following concentrations (5,000; 10,000; 50,000; or 100,000 cells/ml). The resulting EBs were embedded in matrix at 3, 4, 5, 6 or 11 days post-aggregation. The matrices tested were growth factor reduced Matrigel (at a working dilution 1:3) or rat tail collagen type I gels (at working concentrations of 0.4 mg/ml, 1 mg/ml, 1.5 mg/ml or 2 mg/ml) both matrices were purchased from BD biosciences. VEGF concentrations tested were 30 ng/ml and 50 ng/ml.

Neuronal Profiling algorithm
The Neuronal Profiling BioApplication V4 from Thermo Fisher Scientific Inc was used. Detailed information regarding the software/algorithm can be obtained using the link below:
http://www.med.cam.ac.uk/wp-content/uploads/2016/02/NeuronalProfiling_V4_LC06190800.pdf
The software was optimized by adjusting thresholds to identify the core of the embryoid body (EB) as the neuron cell nucleus, the parameter of the EB as the neuron cell body and the neurite detection was optimized to detect fluorescent sprouts as the neurites. See Figure S1C for images from the analysis system.

Immunohistochemistry of EBs in collagen gels
Following fixation, EBs in collagen gels were blocked and permeabilized in 5% FBS/0.3% Triton-X100 in 1xPBS, followed by incubation with PECAM-1 (MEC 13.3) (BD Biosciences), anti-mouse DLL4 (R&D systems) or alpha-smooth muscle actin (α-SMA) (1A4) (Sigma-Aldrich). Bound antibodies were detected by Alexa-Fluor 546 secondary antibodies and nuclei were stained with Hoechst 33342. Subsequently, EBs were transferred to glass slides and mounted in Dako fluorescence mounting medium (Agilent) and imaged using a DMI6000B microscope equipped with DFC345-FX camera and LAS-AF software (Leica).

Dose curve validation of hits
EBs were treated with DMSO, VEGF (50 ng/ml) in the presence of DMSO, or VEGF (50 ng/ml) in the presence of varying concentrations of compounds (0.026 μM-2.1 μM), dispensed using the HP D300 Digital Dispenser (Tecan). On day 7 post-embedding, EBs were fixed and the number of FLK-1^{+ve} sprouts and total fluorescent intensity were measured using the Cellomics VTI platform (Thermo Fischer) with an optimized neuronal profiling algorithm as described above.

Cell, tissue lysates and Western blots
Cell and tissue lysates were prepared with RIPA buffer containing protease/phosphatase inhibitors (Cell Signaling Technology). Lysates were resolved by SDS-PAGE, transferred to PVDF membranes, and probed with primary antibodies. The antibodies were: β-ACTIN (AC-15) (Sigma-Aldrich), RSK (32D7), p-RSK (Thr573), LKB1 (D60C5), p-LKB1 Ser428 (C67A3), RPS6 (54D2), p-RPS6 Ser235/236 (D57.2.2E), SMAD2 (D43B4) XP®, and p-SMAD2 (Ser465/467), all from Cell Signaling Technology. Densitometry was performed using Image Studio Lite (Li-COR Biosciences).

HUVEC tube formation assay and disruption of preformed tubes
As per Invitrogen's instructions, HUVECs (75,600 cells/well) were seeded on Geltrex™-coated plates. HUVECS were then either immediately treated with complete media containing DMSO, VEGF (30 ng/ml)+DMSO, BI-D1870 (2 μM), BIX-RSK2 (2 μM), AZ3146 (2 μM) or SU5416 (4 μM) to determine whether these drugs can disrupt tube formation, or treated with these drugs 13 hours post-seeding to determine whether these drugs can disrupt the networks after they had formed. At the end of the experiment, HUVECS were stained with 2 μg/ml Calcein-AM. Networks were imaged at 13, 21, and 23 hours post-seeding using a DMI6000B microscope equipped with DFC345-FX camera and LAS-AF software (Leica).

Cytotoxicity assay
LL/2 cells were seeded ($2x10^3$ cells/well) into 96-well plates and cultured in growth media. At 24 hours post-seeding, DMSO or varying concentrations of BI-D1870 or AZ3146 (0-10 μM) were dispensed using the HP D300 Digital Dispenser (Tecan) and cells were incubated for an additional 72 hours. The cytotoxic

effects of these compounds were evaluated using the ATPlite 1step detection assay kit (PerkinElmer) as per manufacturer's instructions, and the luminescence signal was read on an Envision plate reader (PerkinElmer).

***In vivo* exposure analysis of BI-D1870 and AZ3146**

BI-D1870 and AZ3146 were administered to three mice at 50 mg/kg via intraperitoneal (i.p) injection for 5 days. On day 5, blood samples were collected 60 min and 120 min after the last injection. Plasma was separated from blood through centrifugation and plasma concentration of compounds was determined by liquid chromatography–mass spectrometry. Mouse health and body weight were monitored daily (See Figure S4B).
